# State of health and inequalities among Italian regions from 2000 to 2021: a systematic analysis based on the Global Burden of Disease Study 2021

**DOI:** 10.1016/S2468-2667(25)00045-3

**Published:** 2025-03-31

**Authors:** Mohsen Naghavi, GBD 2021 Italy Subnational Burden of Disease Collaborators, Giulia Zamagni, Cristiana Abbafati, Benedetta Armocida, Antonella Agodi, Gianfranco Alicandro, Franca Barbic, Martina Barchitta, Matteo Bauckneht, Massimiliano Beghi, Raffaele Bugiardini, Angelo Capodici, Claudia Carletti, Giulia Carreras, Andrea Carugno, Maria Sofia Cattaruzza, Edina Cenko, Sonia Cerrai, Iolanda Cioffi, Sara Conti, Paolo Angelo Cortesi, Giovanni Damiani, Nicole Davis Weaver, Cristian Del Bo', Natalia Fabin, Luca Falzone, Folorunso Oludayo Fasina, Pietro Ferrara, Ottavia E Ferraro, Marco Fonzo, Carla Fornari, Daniela Fortuna, Matteo Foschi, Fabrizio Gemmi, Scott D Glenn, Davide Golinelli, Giuseppe Gorini, Giovanni Guarducci, Stefano Guicciardi, Mihajlo Jakovljevic, Carlo La Vecchia, Francesco Lanfranchi, Paolo Lauriola, Caterina Ledda, Matilde Leonardi, Giancarlo Logroscino, Alessandra Lugo, Lorenzo Giovanni Mantovani, Daniela Martini, Giada Minelli, Antonio Mirijello, Ali H Mokdad, Sabrina Molinaro, Lorenzo Muccioli, Luigi Naldi, Luciano Nieddu, Raffaele Palladino, Paola Pani, Maja Pasovic, Roberto Passera, Paolo Pedersini, Umberto Pensato, Norberto Perico, Daniela Pierannunzio, Alberto Raggi, Giuseppe Remuzzi, Marina Romozzi, Michele Russo, Simona Sacco, Domenico Trico, Mario Valenti, Francesco S Violante, Johan Månsson, Mohsen Naghavi, Luca Ronfani, Lorenzo Monasta

## Abstract

**Background:**

Over the past two decades, the Italian National Health Service has been gradually decentralised, with Italy's 21 regional governments now responsible for managing their health services. This change, coupled with austerity measures and a steadily ageing population, has adversely affected universal health coverage and equity, exacerbating inequalities and regional disparities. This study aimed to analyse time trends and subnational differences in the burden of disease from 2000 to 2019, and from 2019 to 2021 to capture the effects of the COVID-19 pandemic.

**Methods:**

This study uses estimates for Italy from the Global Burden of Diseases, Injuries, and Risk Factors Study 2021. We analyse trends and geographical differences in disease burden from 2000 to 2021. Metrics include life expectancy, health-adjusted life expectancy (HALE), years lived with disability (YLDs), years of life lost (YLLs), and disability-adjusted life-years (DALYs) observed at national, macroregional, and subnational levels. Percent changes in rates, with both all-age and age-standardised rates, and 95% uncertainty intervals (95% UIs) are reported.

**Findings:**

Life expectancy at birth in Italy increased from 79·6 years in 2000 to 83·4 years in 2019, dropped to 82·2 years in 2020 due to COVID-19, and recovered slightly to 82·7 years in 2021. HALE was 70·9 years (95% UI 67·4–73·8) in 2021. Substantial regional disparities were observed: in general, despite higher YLD rates, northern regions had better health outcomes, with higher life expectancy and HALE and lower YLL rates compared with southern regions. Overall, the top causes of YLDs were low back pain (1556·5 [1098·5–2080·2]), falls (926·2 [638·8–1253·8]), and headache disorders (858·0 [173·7–1808·2]). Anxiety and depressive disorders both had substantial increases in the period from 2019 to 2021 (19·8% and 17·3%, respectively). YLDs for Alzheimer's disease and diabetes increased substantially from 2000 to 2019 and 2019 to 2021 (70·6% and 3·0% for Alzheimer's disease and 46·8% and 7·9%, respectively for each timepoint). YLL rates declined for ischaemic heart disease from 2000 (–29·9% in 2019), but increased for Alzheimer's disease and other dementias (54·5%). DALY rates decreased overall from 2000 to 2019, but rose again in 2021 due to the COVID-19 pandemic.

**Interpretation:**

The study highlights considerable regional disparities in Italy's health outcomes, driven by demography, heterogeneous health service quality, and economic inequalities. Addressing the increasing burden of Alzheimer's disease, diabetes, and mental health disorders, as well as regional disparities, requires strengthened preventive measures, equitable health service access, and socioeconomic policies, both at the national and regional levels.

**Funding:**

Bill & Melinda Gates Foundation.

## Introduction

Over the past two decades, the Italian National Health Service (NHS) has gone through a process of regionalisation, leading to increased autonomy in the management of health services by Italy's 21 regional governments (19 regions and two autonomous provinces [APs]). Consequently, regional governments are empowered to determine health plans, governance models, and budget allocations, which are primarily financed by the national government through taxation.^1^ Such a configuration, whose origin can be traced to 2001 with the Title V reform of the Italian Constitution, was followed by a period characterised by austerity policies enacted in response to the 2008 economic crisis, cost-containment measures aimed at reducing the preexisting fiscal deficit, and a reorganisation of health services.^2^ Furthermore, influenced by issues of sustainability,^3^, ^4^, ^5^ in an era of neoliberal economic consensus,^6^ public health services have lacked a timely response, which has in turn led to families responding either through out-of-pocket expenditures or by foregoing treatments. Adequate standards of universal health coverage (UHC) are becoming difficult to maintain. This poses equity issues,^7^, ^8^ which couples with the long-standing economic gap between southern and northern regions. Between 2000 and 2019, families’ health expenditures grew from 26% to 35% of total health expenditures, an effect that was mostly concentrated in the wealthiest regions of the north.^9^ In terms of UHC, the Italian NHS, once considered among the best in the world, is no longer in the top ten according to several global rankings.^10^, ^11^ In 2019, with spending at US$2000 per person per year, and a UHC Index of 89, Italy ranked 20th globally.^12^, ^13^

**Research in context**

**Evidence before this study**

From a search of the literature and the collective expertise of co-authors we found that the Italian population is among the oldest globally, with a median age of 48 years and 24% of the population aged 65 years or older. The total fertility rate has been below 2·1—the threshold for ensuring generational replacement—since 1976. The effect of these factors on the Italian population structure and its epidemiological profile is substantial, with a gradual rise of the fatal and non-fatal burden of chronic non-communicable diseases. Italy is also affected by an unequal distribution of resources among regions, with northern regions being wealthier than southern ones. This is reflected in the uneven quality of health-care services and the different capacities of families to compensate for poor access to and varying quality of local health-care services. Instead of trying to reduce the gap among regions, over the past 20 years several governments have pushed towards increasing regional autonomy in the management of health-care services. Official reports have highlighted the underfunding of the public health service, the shortage of physicians, the increasingly long waiting lists, and the scarcity of local health services for primary care and prevention.

**Added value of this study**

Our Article is based on the Global Burden of Diseases, Injuries, and Risk Factors 2021 (GBD 2021) subnational estimates for Italy, which have been released to the public for the first time in this GBD round. This allowed us to use metrics such as years lived with disability (YLDs), years of life lost due to premature mortality (YLLs), and disability-adjusted life-years (DALYs), coherently linked to life expectancy at birth and health adjusted life expectancy (HALE), in all-age and age-standardised rates to evaluate the burden of ageing at the national and regional level. To the best of our knowledge, this is the most comprehensive evaluation of regional comparisons of health in Italy, with and without adjusting for different population structures in terms of fatal and non-fatal burden. The regional variations appear to have a substantial effect on population health. In 2021, the southern and island macroregions showed a higher fatal burden, both in terms of all-age and age-standardised rates. While the COVID-19 pandemic affected northern regions more than southern ones in 2020, the north recovered in 2021, whereas the South and Islands regions progressively worsened throughout 2020 and 2021. By comparison, northern regions were more highly affected by the non-fatal burden as a consequence of their older population structure. In general, northern regions consistently showed better health outcomes, characterised by higher life expectancy and HALE, which reflects the more advanced health-care systems and lower fatal burden compared with the southern and island regions. A similar pattern emerges when comparing females with males: females, with a 4·5 year higher life expectancy, have a higher non-fatal burden than males. Our findings offer a unified framework for interpreting the complex burden of disease across regions, and they provide valuable confirmation of long-standing questions debated within the Italian epidemiological field, such as the interplay between regional socioeconomic inequalities, health-care system performance, and demographic factors.

**Implications of all the available evidence**

The economic divide among regions in Italy affects the quality and capacity of access to health services, which translates into different profiles of burden across the country. People live longer, but the stagnation of the non-fatal burden is disproportionately affecting regions with older populations and females. Females show a substantially higher life expectancy than males, but accumulate a higher non-fatal burden due to chronic conditions such as Alzheimer's disease and diabetes, which both have risen in the top ten causes of DALYs. The burden of anxiety disorders has increased in the past 20 years and even more in 2020 and 2021, while depressive disorders, whose trend was in a slight reduction, showed a strong increase in 2020, and has not reached pre-pandemic levels in 2021. Social policies in support of families are needed to reduce economic and geographical inequalities and increase trust and social wellbeing. Health services should be more focused on the promotion of healthy ageing and the prevention of behavioural and metabolic risk factors.

Population ageing also constitutes a concern. In 2021, the total fertility rate (TFR) in Italy was 1·24,^14^ considerably below the recognised minimum threshold of 2·1 required to guarantee generational replacement.^15^ With more than 24% of the population aged 65 years and older and a median age of 48 years, Italy is the second oldest country in the world after Japan.^16^ This implies further increases in the burden of disease and complexity of illnesses, with a projected rise in the number of people living with long-term comorbidities.

Further concerns come from increasing privatisation, especially for secondary, tertiary, and long-term care,^17^ alongside stagnating economic growth,^18^ rising income inequality,^19^ and unhealthy lifestyles.^20^

In this context, the first-ever release of the Global Burden of Diseases, Injuries, and Risk Factors (GBD) estimation for the Italian regions provides an excellent opportunity for an in-depth examination of the regional burden of diseases and the utility of modelled data within the GBD study 2021 (GBD 2021). GBD 2021 subnational estimates for Italy are available from 1990 to 2021. However, our analyses focus on the period from 2000 to 2021 to assess the effect of the 2001 constitutional reform and the subsequent cost-containment measures and reorganisation of health services. This study aims to analyse temporal trends and geographical differences in the burden of disease at the subnational level from 2000 to 2019 to capture long-term trends, and from 2019 to 2021 to provide a focused analysis of the COVID-19 pandemic years.

The manuscript was prepared following the GBD Protocol and within the framework of the GBD Collaborator Network.

## Methods

### Study design and unit of analysis

Italy is the fifth-largest data contributor to the GBD Study, with 3409 sources including essential registration systems, national surveys, registries, and administrative data. All sources used to generate GBD estimates are listed in the Global Health Data Exchange repository.^21^

GBD analyses adhere to the GATHER standards.^22^ A detailed description of methods used in GBD 2021 is provided in the capstone papers.^23^, ^24^, ^25^

### Data analysis

We used five metrics to evaluate the burden of disease in the present analyses: life expectancy at birth, health-adjusted life expectancy (HALE), years lived with disability (YLDs), years of life lost (YLLs), and disability-adjusted life-years (DALYs). YLDs, YLLs, and DALYs were reported as all-age and age-standardised rates per 100 000 people, accompanied by 95% uncertainty intervals (UIs). UIs were obtained for each metric using the 25th and 75th ordered values from a 1000-draw posterior distribution. Age-standardised rates were calculated using the GBD standard population structure.

Estimates for Italy were presented at the national level, for five macroregions (ie, Northwest, Northeast, Centre, South, and Islands), and 21 subnational locations (ie, 19 regions and two autonomous provinces), following the Nomenclature des Unités Territoriales Statistiques (NUTS-1 and NUTS-2).^26^ The Northwest macroregion included Piemonte, Valle d’Aosta, Liguria, and Lombardia (Lombardy); the Northeast included the AP of Bolzano, the AP of Trento, Veneto, Friuli-Venezia Giulia, and Emilia-Romagna; the Centre included Toscana (Tuscany), Marche, Umbria, and Lazio; the South included Abruzzo, Molise, Campania, Puglia, Basilicata, and Calabria; and the Islands comprised Sicilia (Sicily) and Sardegna (Sardinia).

Trends of life expectancy, HALE, all-cause YLD, and all-cause YLL rates between 2000 and 2021 were reported separately for males and females. Health trends were analysed from 2000 up to 2019 to provide an unaltered view of the pre-pandemic burden of disease. Changes from 2019 to 2021 were calculated to capture what occurred during the COVID-19 outbreak.

The 20 leading third-level causes of YLDs, YLLs, and DALYs were ranked from 2000 to 2019 and from 2019 to 2021 to evaluate changes in the national burden before and after the COVID-19 pandemic and to assess the impact of COVID-19. Of these 20 causes, the ten leading causes in 2019 and their burdens were also reported by macroregion and region to facilitate the identification of subnational patterns. Percent changes in rates were calculated from 2000 to 2019 and then from 2019 to 2021. As a complement to the analysis of GBD 2021 estimates, we considered the percentage of family health expenditures from 2000 to 2019 over the total health expenditure, from the Health for All database. These data are relevant to show, in a presumably universal health-care system, if and how families are compensating for shortages in the system itself, and whether the compensation rate changed over time.

### Statistical analysis

The overall analysis followed two paths: first, the assessment of the actual burden of disease through all-age rates, which allowed for the assessment of the burden of disease without taking into account the effect of different population structures in different regions; and second, the comparison of health status across years, regions, and macroregions using age-standardised rates, which allowed comparisons to be drawn across all regions as though they all had the same age structure.

### Role of the funding source

The funder of this study had no role in study design, data collection, data analysis, data interpretation, the writing of the report, or the decision to submit the manuscript for publication.

## Results

### Life expectancy and health-adjusted life expectancy

**Figure 1:**
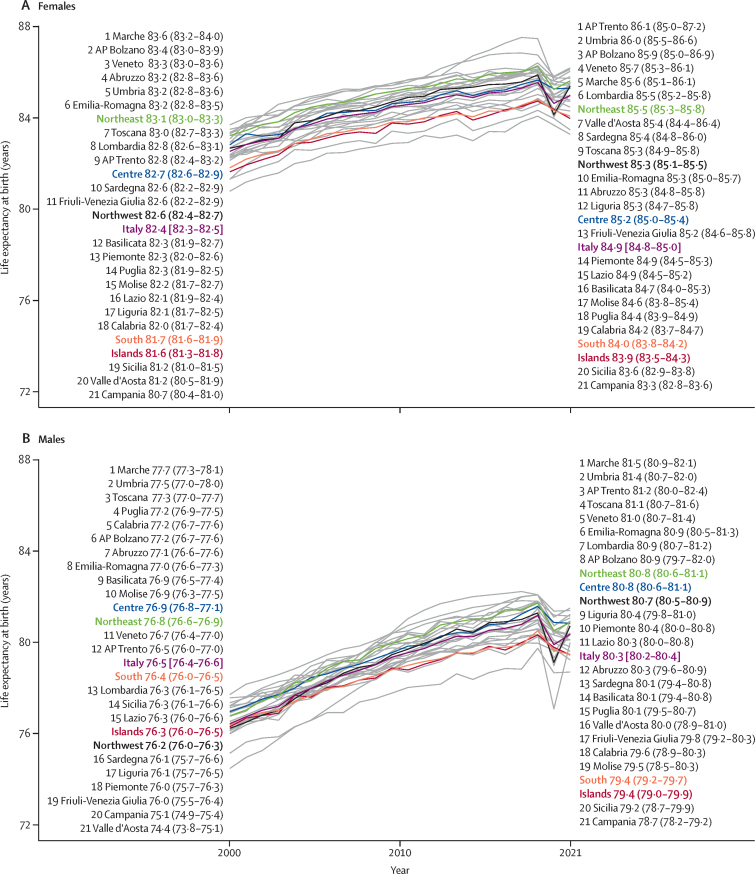
Trends of LE by region and macroregions in Italy for females (A) and males (B) between 2000 and 2021 Estimates are reported with 95% UIs. AP=Autonomous Province. UI=uncertainty interval.

**Figure 2:**
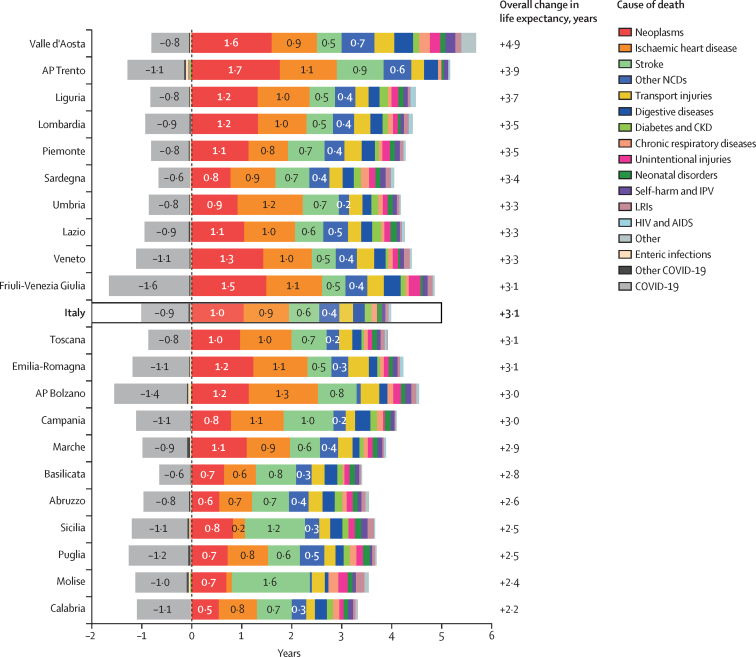
Percentage change in life expectancy at birth by cause of death and region between 2000 and 2021 AP=Autonomous Province. NCDs=non-communicable diseases. CKD=chronic kidney disease. IPV=interpersonal violence. LRIs=lower respiratory infections.

In Italy, life expectancy at birth increased from 79·6 years in 2000 to 83·4 years in 2019. It dropped to 82·2 years in 2020 and increased slightly in 2021 (82·7 years), but did not reach the pre-pandemic values. Females had a higher life expectancy than males at all timepoints. Between 2000 and 2021, life expectancy increased from 76·5 years to 80·3 years for males and from 82·4 years to 84·9 years for females (figure 1). The greatest improvements were due to reductions in the fatal burden of neoplasms (adding 1·0 year to life expectancy) and ischaemic heart disease ([IHD] adding 0·9 years), while COVID-19 entailed a 0·9 year reduction in life expectancy (figure 2). The Northeast (a 4·4 year increase) had the greatest increases in life expectancy, followed by the Northwest (a 4·3 year increase).

Overall, HALE increased from 68·5 years (95% UI 65·2–71·3) in 2000 to 70·9 years (67·4–73·8) in 2021 (appendix 2 p 2). In 2021, females and males could expect to live in good health for 71·4 years (67·5–74·8) and 70·2 years (67·3–72·7), respectively. However, the steepest gain between 2000 and 2021 was found in males (3·1 years *vs* 1·6 years for females), highlighting a trend towards the reduction in sex-based differences. Marked differences were found among geographical areas, with northern regions reaching higher HALE values than the South and island macroregions (appendix 2 p 2). In 2020, due to COVID-19, HALE fell to 70·7 years (67·3–73·6), leading to a loss of 1·0 year of life expectancy compared to 2019, with the Northwest having the largest decrease (1·7 years). In 2021, the Northwest, Northeast, and Centre macroregions all saw improvements towards pre-pandemic values, while HALE continued to decrease in the South.

### Years lived with disability

In Italy, all-age YLD rates slightly increased from 13 839·8 in 2000 to 14 508·0 in 2019, showing a steeper increase in 2020 and 2021 (14 892·8 and 15 056·0, respectively). In general, YLD rates were always higher for females when compared with males, and lower in the South and Island macroregions compared with the Northeast, Northwest, and Centre regions. In particular, Liguria, the region with the oldest population in the country, showed the highest all-age YLD rates over the whole period for both sexes (appendix 2 p 3).

**Figure 3:**
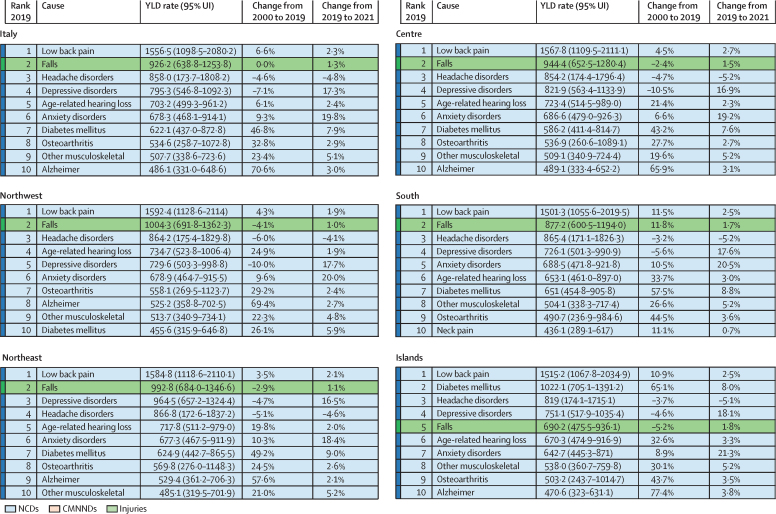
Ten leading causes of all-age YLDs in 2019 in Italy and the five macroregions between 2000 and 2021 Rates are expressed per 100 000 people. Changes indicate the YLD rate change in percent. Alzheimer=Alzheimer's disease and other dementias. CMNNDs=communicable, maternal, neonatal, and nutritional disorders. NCDs=non-communicable diseases. UI=uncertainty interval. YLD=years lived with disability.

Overall, the three leading causes of all-age YLD rates in 2019 were low back pain (1556·5 [95% UI 1098·5–2080·2]), falls (926·2 [638·8–1253·8]), and headache disorders (858·0 [173·7–1808·2]), with the same ranking in 2000 and 2019 (figure 3 and appendix 2 p 4).

Sex differences were observed in the ranking of non-fatal burden of disease causes (appendix 2 pp 5–6). Low back pain was the primary cause for both males and females in both 2000 and 2021. However, males had more falls and age-related hearing loss, while females were more affected by headaches and depressive disorders.

In general, a sharp peak in the non-fatal burden of mental disorders (depressive and anxiety disorders) was found in all macroregions during the 2 years of the COVID-19 pandemic; overall there was an increase of 17·3% for depressive disorders and 19·8% for anxiety disorders from 2019 to 2021 (figure 3).

When age standardisation was applied, less inter-regional variability emerged in terms of all-cause YLDs, with the highest burden faced by the Northeast (appendix 2 p 7). Although the top three causes of age-standardised YLDs were the same over time (ie, low back pain, headache disorders, and depressive disorders), their burden slightly decreased in 2019 compared to 2000 (appendix 2 p 8). Stratifying by sex (appendix 2 pp 9–10), in females, anxiety disorders rose from the fifth position in 2000 to the third position in 2019. Overall, standardisation further highlighted the increasing burden of mental disorders in all macroregions (appendix 2 p 11). Results by region are reported in appendix 2 (pp 12–27).

### Years of life lost

For the all-age all-cause YLL rates per 100 000 people in the 21 Italian regions and autonomous provinces from 2000 to 2021 (appendix 2 p 28), Liguria, the region with the oldest population, showed the highest fatal burden of YLLs for both males and females.

**Figure 4:**
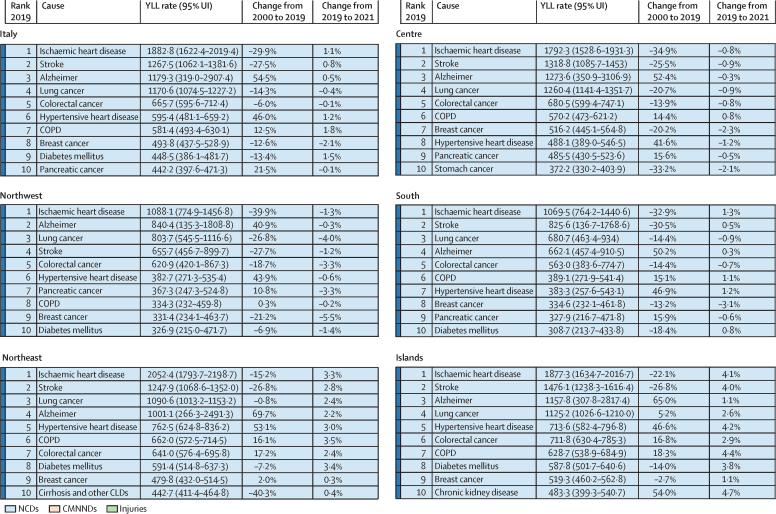
Ten leading causes of all-age YLLs in 2019 in Italy and the five macroregions between 2000 and 2021 Rates are expressed per 100 000 people. Changes indicate the YLL rate change in percent. Alzheimer=Alzheimer's disease and other dementias. COPD=chronic obstructive pulmonary disease. CLDs=chronic liver diseases. CMNNDs=communicable, maternal, neonatal, and nutritional disorders. NCDs=non-communicable diseases. UI=uncertainty interval. YLL=years of life lost.

IHD caused the largest overall YLL rate in Italy in both 2000 and 2019, but with a declining trend over time (–29·9% in 2019; figure 4, appendix 2 p 29). The highest increase was found for Alzheimer's disease and other dementias (54·5%), rising from 763·1 (95% UI 195·7–1957·0) in 2000 to 1179·3 (319·0–2907·4) in 2019.

In 2019, the top three causes of YLLs for males were IHD (2307·6 [95% UI 2120·9–2409·8]), lung cancer (1662·8 [1554·4–1740·2]), and stroke (1202·5 [1081·4–1271·4]); these three leading causes remained unchanged from 2000, despite their decreasing burden (appendix 2 p 30). The three leading causes of YLLs for females in 2019 were Alzheimer's disease and other dementias (1606·6 [450·5–3807·2]), IHD (1480·4 [1155·7–1657·9]), and stroke (1329·1 [1038·4–1493·4]; appendix 2 p 31). In 2000, Alzheimer's disease was the fourth leading cause of YLL for females, with IHD first and stroke second. Breast cancer moved from the third to the fourth position, with a decrease of 12·6%.

In the five macroregions, YLL rankings were dominated by cardiovascular diseases, neurological disorders, and cancers, while diabetes appeared among the first ten causes in all macroregions except the Northwest (figure 4).

**Figure 5:**
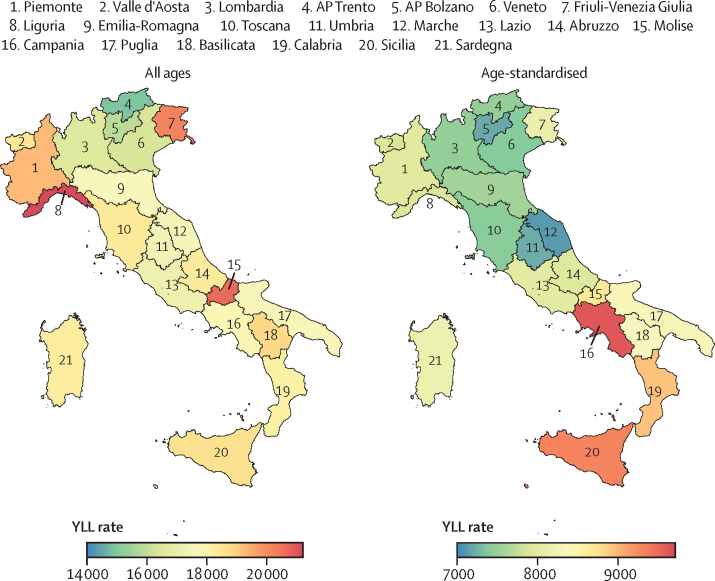
All-age versus age-standardised YLL rates in Italy by region, 2021 Rates are expressed per 100 000 people. Regions and APs are numbered following the classification provided by the National Institute of Statistics. AP=autonomous province. YLL=years of life lost.

Comparing all-age with age-standardised YLL rates (figure 5), the effect of all-age YLLs is greater in the regions with an older population (Liguria, Molise, and Friuli-Venezia Giulia), while southern regions appear to be more affected by premature mortality when YLLs are standardised by age.

Age-standardised YLL rates show that the highest fatal burden is faced by the South and Island macroregions (appendix 2 p 32), with IHD as the primary cause of YLLs in both 2000 and 2019 (1446·2 [95% UI 1330·0–1504·2] and 729·9 [652·6–771·2], respectively; appendix 2 p 33). With age standardisation, the three leading causes of fatal burden for males remained unchanged, while breast cancer emerged as the primary cause of YLLs for females in 2019 (appendix 2 pp 34–35).

In all macroregions, age-standardised YLL rates for the ten leading causes declined, with most having considerable decreases from 2000 to 2019, including during the COVID-19 pandemic (appendix 2 p 36). Results by region are reported in appendix 2 (pp 37–52).

### Disability-adjusted life-years

From 2000 to 2019, all-age DALY rates decreased in most regions, with the largest drop in Valle d’Aosta (–12·3% [–14·7% for males and –9·0% for females]). However, regions in the South and Islands showed slightly increasing or stable DALY rates (appendix 2 p 53). Most regions showed a descending trend from 2000 to 2010, followed by a flattening of the curves, more pronounced in the female population and in the South and Islands. The trend further worsed around 2015. The overall decrease in DALYs was mainly attributable to a reduction in the fatal burden of disease, as YLDs remained relatively constant over time. However, the COVID-19 pandemic led to increasing YLLs in 2021.

IHD was the highest cause of DALY burden in both 2000 and 2019, despite a declining trend (–29·0%; DALY rates of 2783·5 [95% UI 2542·2 to 2911·2] in 2000 *vs* 1975·7 [1717·0 to 2118·7] in 2019; appendix 2 pp 54–55). The highest increase was found for the burden of Alzheimer's disease and other dementias (58·9%), rising from the sixth cause of DALYs in 2000 (1048·1 [481·8 to 2231·5]) to the second cause in 2019 (1665·4 [789·0 to 3382·9]). Road injuries fell from the seventh position in 2000 (964·4 [888·0 to 1059·4]) to the 23rd position in 2019 (411·7 [365·4 to 466·6]), showing the highest decrease over time (–57·3%).

The first three causes for males were the same in both 2000 and 2019, with DALYs in 2019 of the top causes decreasing since 2000 (in 2019, IHD 2420·2 [95% UI 2233·5–2537·6], lung cancer 1684·7 [1572·4–1765·0]), and stroke 1383·5 [1262·6–1474·6]; appendix 2 p 56). Although the top three positions in the ranking remained unchanged, substantial improvements occurred over time. In 2019, the leading cause of DALYs for females was Alzheimer's disease (2265·5 [1084·1–4481·7]), rising from the fourth position in 2000 (57·1%). Low back pain was the second largest cause (1802·0 [1283·1–2398·5]), followed by IHD (1554·6 [1228·7–1734·8]; appendix 2 p 57).

After the COVID-19 pandemic Italy had trend reversals for some DALY rates (appendix 2 p 54). For example, the burden of depressive disorders declined by 7·1% between 2000 and 2019, but increased steeply in 2021 (17·3%; the largest reversal of the top ten causes of all-age DALYs). The burden of diabetes increased substantially over time, following different rates of change across the five macroregions. The Island and South macroregions had the highest DALY rates due to diabetes both in 2000 (1302·6 [95% UI 1111·2–1531·1] and 1050·5 [917·4–1215·1], respectively) and 2019 (1242·4 [1045·7–1596·9] and 1609·9 [1299·2–1981·8], respectively), with higher rates of increase from 2000 to 2019 when compared with Italy as a whole (23·6% for the Islands and 18·3% for the South *vs* 13·7% for Italy overall).

When age standardisation was applied, the progressive decline of the fatal burden and the stability of YLDs emerged most clearly, as YLLs constituted less than 50% of DALYs in all regions in both 2019 and 2021 (appendix 2 p 58). In terms of age-standardised DALY rates, the burden affected the South and Island macroregions more than the Northeast, Northwest, and Centre.

In 2019, the primary cause of age-standardised DALYs in Italy was low back pain (1074·1 [95% UI 765·3 to 1443·7]), followed by headache disorders (826·6 [135·7 to 1770·0]) and IHD (769·8 [692·7 to 816·5]; appendix 2 p 59). The sharpest decline between 2000 and 2019 was found for the burden of road injuries (–60·1%). In terms of age-standardised DALY rates, the burden of Alzheimer's disease and other dementias was largely stable over time. Age-standardised DALY rates for other musculoskeletal disorders increased the most (15·8%), from 345·7 (237·5 to 488·2) in 2000 to 400·2 (275·3 to 558·0) in 2019.

The 20 leading causes of age-standardised DALYs for males and females, respectively, in 2000 and 2019 are shown in appendix 2 (pp 60–61). Low back pain was the leading cause of age-standardised burden in all macroregions. The Islands had the lowest age-standardised burden of headache disorders, falls, and lung cancer, and the highest age-standardised burden due to diabetes and stroke (appendix 2 p 62). The South has the lowest age-standardised burden of stroke and the highest age-standardised burden of IHD. Depressive disorders were highest in the Northeast. Anxiety and depressive disorders moved up the overall ranking, with a very high peak during the COVID-19 pandemic. The Northwest was the only region without diabetes in the first ten causes of age-standardised DALYs, but had the highest age-standardised burden of headache disorders, together with the Northeast. Results by region are reported in appendix 2 (pp 63–78).

### Family health expenditure

The percentage of per capita family health expenditure rose from 2000 to 2019 for all regions and macroregions (appendix 2 p 79). The trends show a steeper increase between 2007 and 2009, with a peak in 2011 and a flattening trend since then. Wealthier macroregions, such as the Northeast and Northwest, show higher percentage expenditures than the South and Islands throughout the entire period.

## Discussion

The findings from the GBD 2021 study show substantial health heterogeneities in Italy and among Italian regions in life expectancy, HALE, YLDs, and YLLs.

### Life expectancy and HALE

From 2000 to 2019, Italy saw an overall increase in LE, which decreased in 2020 due to the COVID-19 pandemic, and only partly recovered in 2021. However, the country's demographic challenges stem primarily from an ageing population, further exacerbated by a persistently low TFR, which has remained well below the replacement level of 2·1 since 1976; and rising dependency ratios, rather than from fluctuations in life expectancy. The overall increase in life expectancy was mainly due to the reductions in YLL rates from neoplasms, IHD, stroke, and transport injuries. For neoplasms, reductions in YLL rates might be attributed to advancements in early detection and treatment strategies. Screening programmes could also have increased the likelihood of early detection and treatment of cancers, particularly breast, cervical, and colorectal cancers.^27^, ^28^ Additionally, a considerable positive effect on life expectancy came from public health interventions targeting cancer risk factors (eg, smoking cessation programmes, reduction in occupational and environmental carcinogens, and infection control [ie, human papillomavirus vaccination]).^27^, ^29^ For IHD and stroke, the reduction of the burden of behavioural and metabolic risk factors, and the improvement of preventive measures and pharmacological solutions, all had crucial roles. Transport injury-driven mortality was reduced due to a combination of improved road safety measures and vehicular safety devices, first aid interventions, and post-injury physiotherapy and rehabilitation, and alcohol-related policies, such as the enforcement of blood alcohol limits and public awareness initiatives, which had a crucial role in reducing alcohol-impaired driving. However, the positive increase in life expectancy is counterbalanced by the increase in the YLLs rate for Alzheimer's disease and other dementias as a consequence of population ageing.^16^ In addition, coverage of screening programs and quality of treatment is not homogeneous throughout the country, and is associated with higher levels of premature mortality in the less economically resourced southern regions.^30^

Regional analysis of life expectancy reveals that several southern regions are worse off than their northern counterparts. This geographical gradient, well reported in the literature,^31^ is also evident in HALE, indicating that not only do individuals in the Northern and Centre regions live longer, but they also enjoy more years in good health compared to those in the South and Islands. The sex gap in life expectancy has narrowed, yet a 4·5 year difference still exists in favour of women. HALE showed faster growth in males than in females, although a gap of 1·3 years was still present in 2021.

### Years lived with disability

YLD rates have steadily increased from 2000 to 2021, particularly post-pandemic, driven primarily by non-fatal conditions such as low back pain, falls, and headache disorders. Mental health disorders, including anxiety and depression, have surged in prevalence, particularly in the aftermath of COVID-19. Sex differences are prominent, with women experiencing higher YLD rates as a consequence of a greater burden of mental health conditions. Regional disparities are stark, with the highest YLD rates in Liguria and in the Northeast as a consequence of an older population structure. Additionally, behavioural and metabolic risk factors—such as smoking, high BMI, and hypertension—contribute to these disparities, as the risk factors are unevenly distributed across regions and influence both non-fatal and fatal disease burden.

### Years of life lost

The fatal burden of disease, as reflected in all-age YLL rates, has shown a general decline due to decreases in mortality from IHD disease and, in men, from lung cancer. However, Alzheimer's disease and other dementias have substantially increased across the entire country. Liguria is the region with the highest all-age rates for stroke, lung cancer, and Alzheimer's disease, while Molise has the highest rates due to IHD. Liguria and Molise have, on average, the oldest population in Italy. By contrast, the South and Islands macroregions, particularly Campania, Sicilia, and Calabria, showed higher rates of age-standardised YLLs. Campania has the highest age-standardised YLL rates for IHD, lung cancer, and Alzheimer's disease, and is joint with Sicilia for stroke. Campania and Sicilia are among the regions with the lowest median population age, and these high rates of age-standardised YLLs reveal how the burden can also be affected by the quality of health services in these southern regions with comparatively lower monetary resources. Austerity policies introduced in response to the 2008 economic crisis led to cost-containment measures and service reorganisation within the Italian NHS.^2^ Ten regions (Abruzzo, Calabria, Campania, Lazio, Liguria, Molise, Piemonte, Puglia, Sardegna, and Sicilia), seven of which are in the South and Islands, also faced additional repayment plans to restore financial stability, further reducing available expenditures. These measures, coupled with increasing privatisation, particularly in secondary, tertiary, and long-term care, exacerbated the disparities, leaving some regions struggling to deliver equitable health care.

### Disability-adjusted life-years

DALY rates have decreased in northern regions but remained stable or slightly increased in the South and Island regions. A flattening trend can also be noticed since 2010**.** The reduction in fatal burdens has been more pronounced than in non-fatal burdens, meaning that a longer life expectancy is not translating into healthy ageing. The COVID-19 pandemic reversed some of these gains, leading to an overall increase in DALY rates in 2021.

### Main findings

Our findings highlight substantial regional differences in health outcomes, driven by a combination of demographic factors, health-care service quality, and socioeconomic inequalities. Overall, northern regions consistently show better outcomes in terms of LE, HALE, and YLLs, which can be partially attributed to more advanced health-care systems and greater resource allocation. Conversely, southern regions face higher age-standardised YLLs and DALYs, reflecting systemic challenges in health care access and quality.

Our findings carry important implications for public health policy and practice in Italy, emphasising the persistent sex disparities in life expectancy and HALE and relevant geographical and sociodemographic differences. These disparities reflect the uneven distribution of socioeconomic resources, health care quality, and demographic patterns with varying degrees of population ageing among regions. Moreover, the long-standing divide between northern and southern regions could become even greater if the constitutional reform on differentiated autonomy is adopted. This reform, which would enhance regional financial autonomy, risks enabling wealthier regions to maintain adequate health services, while economically disadvantaged regions could face further challenges. In addition, the increasing privatisation of the Italian NHS, endorsed by the Italian Antitrust Authority, poses challenges to UHC. While promoting the growth of the private sector, this approach further hinders the equitable provision of high-quality health services and equal access to treatments, especially in regions already struggling with resource constraints.^32^, ^33^, ^34^

Efforts to promote healthy ageing, prevent chronic diseases, and improve access to health-care services should be prioritised to sustain the improvements in life expectancy and enhance overall wellbeing. Healthy ageing should be promoted by implementing active-ageing policies, such as geriatric care services, social inclusion, mental wellbeing policies, and providing support to caregivers.^35^

Additionally, the increasing burden of disability, particularly musculoskeletal disorders, calls for comprehensive strategies to promote physical activity and a healthy diet, prevent injuries, and provide adequate rehabilitation services. Strengthening primary care and community-based initiatives can facilitate early detection and management of chronic conditions, thereby reducing the effects of disability.

The cost of disability in a population should be reduced with strong, empirically-driven preventive health policies. Interventions aimed at reducing the burden of disease in the Italian population are needed, alongside an investment in human capital. The rising burden of mental health disorders, exacerbated by the pandemic, highlights the need for robust mental health services. Expanding access to mental health care, reducing stigma, and integrating mental health services into primary health care can address the growing mental health crisis.^36^ Moreover, the implementation of real-time surveillance systems is crucial to enable timely monitoring and intervention, providing actionable insights to guide public health strategies and improve mental health outcomes.

To reduce sex disparities, the specific health needs of males and females must be addressed through targeted interventions.^37^ The regional data made available by the GBD 21 study can have a relevant role, allowing the identification of causes of disease and risk factors stratified by sex.

To overcome the geographical gradient in outcomes, the quality of health care and access to social and health services should be guaranteed universally. The quality of health care services should be made uniform between the different Italian regions, and regions with lower gross domestic product and a higher proportion of families for whom economic costs are the main obstacle to accessing health services, such as in southern Italy, should adopt policies for reducing such barriers.

The difficulty in compensating for the insufficient health-care services with out-of-pocket expenditures is particularly evident in families living in the South and Island regions, which tend to give up medical treatment (in less wealthy areas of the country, more families cannot afford to compensate, and their lower percentage of expenditure indicates a difficulty in obtaining proper health-care services).^9^ Despite, on average, families in the north trying to compensate for inadequate access to health care, a substantial proportion cannot afford it, highlighting the importance in planning social policies to provide proper social support for families.^38^ The Instituto Nazionale di Statistica reported that in 2021, average family expenses for health were €1454 in the Northeast, €1452 in the Northwest, €1272 in the Centre, €1124 in the islands, and €1061 in the South.^39^

It is therefore imperative to move away from privatisation, prioritise the reinforcement of the public health-care system, ensure the provision of affordable services to reduce out-of-pocket expenditures, and effectively address disparities. Preventive health strategies should focus on further reducing behavioural risk factors—such as smoking and alcohol consumption, which are still major contributors to the disease burden. Metabolic risk factors—eg, high blood pressure and high BMI, which are often linked to lifestyle, require comprehensive public health campaigns and interventions aimed at promoting healthier behaviours. Improving early screening for these factors can help improve the detection and management of conditions before they become severe. Several of these preventive interventions are being promoted by the Italian National Prevention Plan 2020–25, which provides a common framework of objectives for many of the areas relevant to public health.^40^ In this context, the planned reorganisation of local health care services is paramount to effectively implement these preventive measures and improve overall community health outcomes.^41^ The COVID-19 pandemic has highlighted the importance of resilience and adaptability in health-care systems and public health interventions. The strengthening of health-care infrastructures, enhancement of surveillance and response capabilities, and the promotion of health equity will mitigate the effect of future health crises.

### Strengths and limitations

Our analysis has several limitations. Some are inherent to any estimation process, some of which are described for the GBD 2021.^23^, ^24^, ^25^ The use of disability weights, which represent the magnitude of health loss associated with specific health outcomes and are used to calculate YLD, might not fully capture cultural and contextual differences in disease burden. The quality of estimates depends strictly on the quality of data used to feed the models. The quality and quantity of data for Italy, at the national and subnational levels, are high, but still present a series of weaknesses. Conditions not requiring admission to hospital and outpatient hospital visits, such as headache disorders or musculoskeletal disorders, tend to be overlooked. Mental health is also often neglected, as these conditions are dealt with by local services which have a separate data system not yet accessible to the GBD. Hospital outpatient data are not codified per ICD and thus are very difficult to use. General practitioners and primary care paediatricians use separate data collection systems not integrated with other health-care systems, such as hospital discharge records and causes of death. The data from national health surveillance systems are not fully available to the GBD, therefore potentially missing valuable insights into health data. Data on cause of death require 2 years to be made official and published. These inevitable insufficiencies in up-to-date data are consequently reflected in wide UIs for certain conditions. Moreover, we acknowledge that the 2019–21 period might not fully reflect the impact of the COVID-19 pandemic. Hence, our findings should be interpreted as a preliminary assessment of the pandemic's effects on health outcomes. Finally, we realise the manuscript would have been enriched by a detailed analysis of the risk factors associated with the main causes of disease burden, which was not possible in one paper with health estimates. We are, however, in the process of filling this gap, and the paper is in preparation.

### Conclusion

This comprehensive analysis of life expectancy, HALE, YLDs, and YLLs in Italy highlights the crucial need for a multifaceted public health strategy to address regional health disparities and improve overall health outcomes. By prioritising preventive health measures, enhancing health care access, promoting healthy ageing, and addressing economic and social determinants of health, Italy can make substantial strides in reducing the burden of disease and improving the wellbeing of its population. Public health policies must be adaptable and responsive to the evolving health landscape, particularly in the post-pandemic era, to ensure sustainable health improvements for all regions and demographics.

#### GBD 2021 Italy Subnational Burden of Disease Collaborators

#### Affiliations

#### Contributors

#### Data sharing

To download the data used in these analyses, please visit the Global Health Data Exchange GBD 2021 website at http://ghdx.healthdata.org/gbd-2021/sources.

## Declaration of interests

FB declares the position of Secretary of International Commission on Occupational Health Scientific Committee on Cardiology in Occupational Health. MBe declares payment or honoraria from Lundbeck and Angelini. ML declares payment for attending meetings or travel from the European Academy of Neurology Society of Neurology and from the Italian Society of Neurology. GZ, LGM, LMo, and LR declare payments to their institutions from the Italian Ministry of Health in support of this work. RaP declares grants or contracts from the UK Multiple Sclerosis Society and payment or honoraria from Biogen, MSD, Sanofi, and BMS. RoP declares membership of a data safety monitoring board for Fondazione Italiana Linfomi–ETS; an unpaid leadership role at the European Society for Blood and Marrow Transplantation; and past membership of the IRB/IEC Comiato Etico AO SS. SS declares grants or contracts with Novartis and Uriach; consulting fees and payments or honoraria from Novartis, Allergan (AbbVie), Teva, Lilly, Lundbeck, Pfizer, Novo Nordisk, Abbott, and AstraZeneca; support for attending a meeting or travel costs from Lilly, Novartis, Teva, Lundbeck, and Pfizer; receipt of medical equipment, drugs, or materials from Allergan (AbbVie) and Novo Nordisk; and is President of the European Stroke Organisation and Editor-in-Chief of Cephalalgia. DT declares payments or honoraria from AstraZeneca, Eli Lilly, and Novo Nordisk; support for attending meetings or travel from AstraZeneca, participation on data safety monitoring boards at Amarin, Npejringer Ingelheim, and Novo Nordisk; receipt of medical equipment, drugs, or materials from Abbott and PharmaNutra; and leadership roles at the European Association for the Study of Diabetes Early Career Academy and Committee on Clinical Affairs.
