# Supplementary material for: State of health and inequalities among Italian regions from 2000 to 2021: a systematic analysis based on the Global Burden of Disease Study 2021
Source: Lancet Public Health. 2025 Mar 31;10(4):e309–20. doi: 10.1016/S2468-2667(25)00045-3 (PMC11962357; doi:10.1016/S2468-2667(25)00045-3)
Supplement: Supplementary appendix 1 [file mmc1.pdf]

THE LANCET
Public Health

Supplementary appendix 1

This appendix formed part of the original submission and has been peer reviewed. We post it as supplied by the authors.

Supplement to: GBD 2021 Italy Subnational Burden of Disease Collaborators. State of health and inequalities among Italian regions from 2000 to 2021: a systematic analysis based on the Global Burden of Disease Study 2021. *Lancet Public Health* 2025; **10:** e309–20.

Appendix 2: Authorship appendix to “State of health and inequalities among Italian regions from 2000 to 2021: a systematic analysis based on the Global Burden of Disease Study 2021”

This appendix provides further authorship detail for “State of health and inequalities among Italian regions from 2000 to 2021: a systematic analysis based on the Global Burden of Disease Study 2021”

Table of Contents GBD 2021 Europe Life Expectancy

Collaborators **2**
Affiliations **3**
Authors’ Contributions **5**
- Providing data or critical feedback on data sources 5
- Developing methods or computational machinery 5
- Providing critical feedback on methods or results 5
- Drafting the work or revising it critically for important intellectual content 5
- Managing the estimation or publications process 5

GBD 2021 Italy Subnational Burden of Disease Collaborators

Full Name

Giulia Zamagni,* Cristiana Abbafati,† Benedetta Armocida,‡ Antonella Agodi, Gianfranco Alicandro, Franca Barbic, Martina Barchitta, Matteo Bauckneht, Massimiliano Beghi, Raffaele Bugiardini, Angelo Capodici, Claudia Carletti, Giulia Carreras, Andrea Carugno, Maria Sofia Cattaruzza, Edina Cenko, Sonia Cerrai, Iolanda Cioffi, Sara Conti, Paolo Angelo Cortesi, Giovanni Damiani, Nicole Davis Weaver, Cristian Del Bo', Natalia Fabin, Luca Falzone, Folorunso Oludayo Fasina, Pietro Ferrara, Ottavia E Ferraro, Marco Fonzo, Carla Fornari, Daniela Fortuna, Matteo Foschi, Fabrizio Gemmi, Scott D Glenn, Davide Golinelli, Giuseppe Gorini, Giovanni Guarducci, Stefano Guicciardi, Mihajlo Jakovljevic, Carlo La Vecchia, Francesco Lanfranchi, Paolo Lauriola, Caterina Ledda, Matilde Leonardi, Giancarlo Logroscino, Alessandra Lugo, Lorenzo Giovanni Mantovani, Daniela Martini, Giada Minelli, Antonio Mirijello, Ali H Mokdad, Sabrina Molinaro, Lorenzo Muccioli, Luigi Naldi, Luciano Nieddu, Raffaele Palladino, Paola Pani, Maja Pasovic, Roberto Passera, Paolo Pedersini, Umberto Pensato, Norberto Perico, Daniela Pierannunzio, Alberto Raggi, Giuseppe Remuzzi, Marina Romozzi, Michele Russo, Simona Sacco, Domenico Trico, Mario Valenti, Francesco S Violante, Johan Månsson, Mohsen Naghavi, Luca Ronfani, Lorenzo Monasta.§

* First author
† Second author
‡ Third author
§ Senior author

Affiliations

Clinical Epidemiology and Public Health Research Unit (G Zamagni MSc, C Carletti MSc, P Pani PhD, L Ronfani PhD, L Monasta DSc), Burlo Garofolo Institute for Maternal and Child Health, Trieste, Italy; Department of Legal and Economic Studies (Prof C Abbafati PhD), Department of Public Health and Infectious Diseases (M S Cattaruzza PhD), La Sapienza University, Rome, Italy; Department of Cardiovascular, Endocrine-Metabolic Diseases and Aging (B Armocida MD), Istituto Superiore di Sanità (ISS), Rome, Italy; Department of Medical and Surgical Sciences and Advanced Technologies "GF Ingrassia" (Prof A Agodi PhD, M Barchitta PhD), Department of Biomedical and Biotechnological Sciences (L Falzone PhD), Department of Clinical and Experimental Medicine (Prof C Ledda PhD), University of Catania, Catania, Italy; Department of Pathophysiology and Transplantation (G Alicandro PhD), Department of Food, Environmental and Nutritional Sciences (DeFENS) (I Cioffi PhD, C Del Bo' PhD, Prof D Martini PhD), IRCCS Istituto Ortopedico Galeazzi (Galeazzi Orthopedic Institute IRCCS) (G Damiani MD), Department of Clinical Sciences and Community Health (Prof C La Vecchia MD), Università degli Studi di Milano (University of Milan), Milan, Italy; Cystic Fibrosis Center (G Alicandro PhD), Fondazione IRCCS Ospedale Maggiore Policlinico (IRCCS "Ca' Granda Maggiore Policlinico" Hospital Foundation), Milan, Italy; Department of Biomedical Sciences (F Barbic PhD, N Fabin MD, M Valenti MD), Humanitas University, Milan, Italy; Department of Epidemiology and Biostatistics (F Barbic PhD), Western University, London, ON, Canada; Department of Health Sciences (DISSAL) (M Bauckneht PhD), University of Genoa, Genova, Italy; Department of Nuclear Medicine (M Bauckneht PhD), IRCCS Ospedale Policlinico San Martino, Genova, Italy; Department of Mental Health (M Beghi MD), Health Services Research, Evaluation and Policy Unit (Prof D Golinelli MD), AUSL della Romagna, Ravenna, Italy; Department of Medical and Surgical Sciences (Prof R Bugiardini MD, E Cenko MD, Prof F S Violante MD), Department of Biomedical and Neuromotor Sciences (S Guicciardi MD, L Muccioli MD), University of Bologna, Bologna, Italy; Unit of Hygiene and Public Health (A Capodici MD), Romagna Local Health Authority, Forlì-Cesena, Italy; Interdisciplinary Research Center for Health Science (A Capodici MD), Sant'Anna School of Advanced Studies, Pisa, Italy; Oncological Network, Prevention and Research Institute (G Gorini MD), Institute for Cancer Research, Prevention and Clinical Network, Florence, Italy (G Carreras PhD); Dermatology Unit (A Carugno PhD), University of Insubria, Varese, Italy; Institute of Clinical Physiology (S Cerrai MSc), Italian National Council of Research, Pisa, Italy; General Administration (S Conti PhD), Research Center on Public Health (CESP), School of Medicine and Surgery (P Cortesi PhD), Center for Public Health Research (P Ferrara PhD), Department of Medicine and Surgery (C Fornari PhD), School of Medicine and Surgery (Prof L G Mantovani DSc), University of Milan Bicocca, Monza, Italy; Instituto Auxologico Italiano IRCCS (Italian Auxological Institute) (P Cortesi PhD), IRCCS, Milan, Italy; Department of Dermatology (G Damiani MD), Case Western Reserve University, Cleveland, OH, USA; Institute for Health Metrics and Evaluation (N Davis Weaver MPH, S D Glenn MSc, Prof A H Mokdad PhD, M Pasovic MEd, J Månsson MS, Prof M Naghavi PhD), Department of Health Metrics Sciences, School of Medicine (Prof A H Mokdad PhD, Prof M Naghavi PhD), University of Washington, Seattle, WA, USA; Dermatology Unit (M Valenti MD), IRCCS Humanitas Research Hospital, Milan, Italy (N Fabin MD); Epidemiology and Biostatistics Unit (L Falzone PhD), IRCCS Pascale, Naples, Italy; Department of Veterinary Tropical Diseases (Prof F O Fasina PhD), University of Pretoria, Pretoria, South Africa; Animal Production and Health Division (EMPRES) (Prof F O Fasina PhD), Food and Agriculture Organization of the United Nations, Rome, Italy; Laboratory of Public Health (P Ferrara PhD, Prof L G Mantovani DSc), Instituto Auxologico Italiano IRCCS (Italian Auxological Institute), Milan, Italy; Department of Public Health Experimental and Forensic Medicine (O E Ferraro PhD), University of Pavia, Pavia, Italy; Department of Cardiac, Thoracic, Vascular Sciences and Public Health (M Fonzo MD), University of Padova, Padova, Italy; Innovation in Healthcare and Social Services Department (D Fortuna MSc), Emilia-Romagna Region,

Bologna, Italy; Department of Neuroscience (M Foschi MD), Multiple Sclerosis Research Center, Ravenna, Italy; Department of Biotechnological and Applied Clinical Sciences (M Foschi MD), University of L'Aquila, L'Aquila, Italy; Quality and Equity Unit (F Gemmi MD), Regional Health Agency of Tuscany, Firenze, Italy; Postgraduate School in Hygiene and Preventive Medicine (F Gemmi MD), University of Florence, Florence, Italy; Department of Life Science, Health, and Health Professions (Prof D Golinelli MD), Università degli Studi Link (Link Campus University), Rome, Italy; Post Graduate School of Public Health (G Guarducci MD), University of Siena, Siena, Italy; Department of the Health Directorate (S Guicciardi MD), Local Health Authority of Bologna, Bologna, Italy; The World Academy of Sciences UNESCO, Trieste, Italy (Prof M Jakovljevic PhD); Shaanxi University of Technology, Hanzhong, China (Prof M Jakovljevic PhD); Department of Health Sciences (DISSAL) (F Lanfranchi MD), University of Genoa, Genoa, Italy; International Society of Doctors for the Environment, Arezzo, Italy (P Lauriola MD); SC Neurologia, Salute Pubblica e Disabilità (Neurology, Public Health, Disability Unit) (M Leonardi MD), UO Neurologia, Salute Pubblica e Disabilità (The Neurology, Public Health and Disability Unit) (A Raggi PhD), Fondazione IRCCS Istituto Neurologico Carlo Besta (IRCCS Foundation Carlo Besta Neurological Institute), Milan, Italy; Dipartimento di Biomedicina Traslazionale e Neuroscienze (DiBraiN). (Prof G Logroscino PhD), University of Bari Aldo Moro, Bari, Italy; Department of Clinical Research in Neurology (Prof G Logroscino PhD), Fondazione Cardinale Giovanni Panico Hospital, Tricase, Italy; Department of Medical Epidemiology (A Lugo PhD), Mario Negri Institute for Pharmacological Research, Milan, Italy; Unit of Statistics (G Minelli PhD), Istituto Superiore di Sanità, Rome, Italy; Department of Medical Sciences (A Mirijello MD), IRCCS Casa Sollievo della Sofferenza General Hospital, San Giovanni Rotondo, Italy; Institute of Clinical Physiology (S Molinaro PhD), National Research Council, Pisa, Italy; Department of Dermatology (Prof L Naldi MD), San Bortolo Hospital, Vicenza, Italy; GISED Study Center, Bergamo, Italy (Prof L Naldi MD); Department of Humanities and Social Science (L Nieddu PhD), UNINT, University for International Studies in Rome, Rome, Italy; Department of Public Health (R Palladino MD), University of Naples Federico II, Naples, Italy; Department of Primary Care and Public Health (R Palladino MD), Imperial College London, London, UK; Department of Medical Sciences (R Passera PhD), University of Torino, Torino, Italy; Department of Imaging (R Passera PhD), AOU Città della Salute e della Scienza di Torino, Torino, Italy; IRCCS Fondazione Don Carlo Gnocchi, Milan, Italy (P Pedersini MSc); Department of Clinical and Experimental Sciences (P Pedersini MSc), University of Brescia, Brescia, Italy; Department of Biomedical Sciences (U Pensato MD), Humanitas University, Pieve Emanuele (MI), Italy; Mario Negri Institute for Pharmacological Research, Bergamo, Italy (N Perico MD, Prof G Remuzzi MD); National Centre for Disease Prevention and Health Promotion (D Pierannunzio PhD), National Institute of Health, Roma, Italy; Fondazione Policlinico Universitario A. Gemelli (M Romozzi MD), Università Cattolica del Sacro Cuore (Catholic University of Sacred Heart), Rome, Italy; Department of Cardiology (M Russo PhD), S. Maria dei Battuti Hospital, Conegliano, Italy; Department of Neurology (Prof S Sacco MD), University of L'Aquila, L'Aquila, Italy; Department of Clinical and Experimental Medicine (D Trico MD), University of Pisa, Pisa, Italy; Occupational Medicine Unit (Prof F S Violante MD), Sant'Orsola Malpighi Hospital, Bologna, Italy

Authors' Contributions

Providing data or critical feedback on data sources

Cristiana Abbafati, Benedetta Armocida, Paolo Angelo Cortesi, Giovanni Damiani, Luca Falzone, Pietro Ferrara, Davide Golinelli, Mihajlo Jakovljevic, Caterina Ledda, Matilde Leonardi, Giancarlo Logroscino, Johan Månsson, Lorenzo Giovanni Mantovani, Ali H Mokdad, Lorenzo Monasta, Mohsen Naghavi, Luigi Naldi, Luciano Nieddu, Raffaele Palladino, Maja Pasovic, Paolo Pedersini, Norberto Perico, Daniela Pierannunzio, Alberto Raggi, Luca Ronfani, Michele Russo, and Domenico Trico.

Developing methods or computational machinery

Ali H Mokdad, Mohsen Naghavi, and Maja Pasovic.

Providing critical feedback on methods or results

Cristiana Abbafati, Antonella Agodi, Gianfranco Alicandro, Benedetta Armocida, Franca Barbic, Martina Barchitta, Matteo Bauckneht, Massimiliano Beghi, Raffaele Bugiardini, Angelo Capodici, Edina Cenko, Iolanda Cioffi, Sara Conti, Paolo Angelo Cortesi, Giovanni Damiani, Cristian Del Bo', Natalia Fabin, Luca Falzone, Folorunso Oludayo Fasina, Pietro Ferrara, Ottavia E Ferraro, Carla Fornari, Matteo Foschi, Davide Golinelli, Stefano Guicciardi, Mihajlo Jakovljevic, Carlo La Vecchia, Francesco Lanfranchi, Paolo Lauriola, Caterina Ledda, Matilde Leonardi, Giancarlo Logroscino, Lorenzo Giovanni Mantovani, Daniela Martini, Giada Minelli, Antonio Mirijello, Ali H Mokdad, Sabrina Molinaro, Lorenzo Monasta, Lorenzo Muccioli, Mohsen Naghavi, Luigi Naldi, Luciano Nieddu, Maja Pasovic, Roberto Passera, Paolo Pedersini, Daniela Pierannunzio, Marina Romozzi, Luca Ronfani, Michele Russo, Domenico Trico, Mario Valenti, Francesco S Violante, and Giulia Zamagni.

Drafting the work or revising it critically for important intellectual content

Cristiana Abbafati, Antonella Agodi, Gianfranco Alicandro, Benedetta Armocida, Martina Barchitta, Matteo Bauckneht, Massimiliano Beghi, Raffaele Bugiardini, Angelo Capodici, Claudia Carletti, Giulia Carreras, Andrea Carugno, Maria Sofia Cattaruzza, Edina Cenko, Sonia Cerrai, Sara Conti, Paolo Angelo Cortesi, Giovanni Damiani, Nicole Davis Weaver, Cristian Del Bo', Natalia Fabin, Folorunso Oludayo Fasina, Pietro Ferrara, Marco Fonzo, Carla Fornari, Daniela Fortuna, Matteo Foschi, Fabrizio Gemmi, Scott D Glenn, Davide Golinelli, Giovanni Guarducci, Stefano Guicciardi, Mihajlo Jakovljevic, Carlo La Vecchia, Caterina Ledda, Matilde Leonardi, Giancarlo Logroscino, Alessandra Lugo, Lorenzo Giovanni Mantovani, Daniela Martini, Giada Minelli, Antonio Mirijello, Ali H Mokdad, Sabrina Molinaro, Lorenzo Monasta, Mohsen Naghavi, Luciano Nieddu, Raffaele Palladino, Paola Pani, Maja Pasovic, Roberto Passera, Paolo Pedersini, Umberto Pensato, Norberto Perico, Daniela Pierannunzio, Alberto Raggi, Giuseppe Remuzzi, Marina Romozzi, Luca Ronfani, Michele Russo, Simona Sacco, Domenico Trico, Mario Valenti, and Giulia Zamagni.

Managing the estimation or publications process

Benedetta Armocida, Nicole Davis Weaver, Johan Månsson, Ali H Mokdad, Lorenzo Monasta, Mohsen Naghavi, and Maja Pasovic.
