# Supplementary material for: State of health and inequalities among Italian regions from 2000 to 2021: a systematic analysis based on the Global Burden of Disease Study 2021
Source: Lancet Public Health. 2025 Mar 31;10(4):e309–20. doi: 10.1016/S2468-2667(25)00045-3 (PMC11962357; doi:10.1016/S2468-2667(25)00045-3)

THE LANCET
Public Health

Supplementary appendix 2

This appendix formed part of the original submission and has been peer reviewed. We post it as supplied by the authors.

Supplement to: GBD 2021 Italy Subnational Burden of Disease Collaborators. State of health and inequalities among Italian regions from 2000 to 2021: a systematic analysis based on the Global Burden of Disease Study 2021. *Lancet Public Health* 2025; **10:** e309–20.

Supplementary Appendix

Contents

Figure S1. Trends of Health-Adjusted Life Expectancy at birth (HALE) by region between 2000 and 2021 for females (a) and males (b) 2
Figure S2. Trends of all-age Years Lived with Disability (YLDs) by region in Italy between 2000 and 2021 for females (a) and males (b) 3
Figure S3. Leading causes of all-age Years Lived with Disability (YLDs) in Italy in 2000 and 2019 – Both sexes 4
Figure S4a. Leading causes of all-age Years Lived with Disability (YLDs) in Italy in 2000 and 2019 – Males 5
Figure S4b. Leading causes of all-age Years Lived with Disability (YLDs) in Italy in 2000 and 2019 – Females 6
Figure S5. Trends of age-standardised Years Lived with Disability (YLDs) by region in Italy between 2000 and 2021 for females (a) and males (b) 7
Figure S6. Leading causes of age-standardised Years Lived with Disability (YLDs) in Italy in 2000 and 2019 – Both sexes 8
Figure S7a. Leading causes of age-standardised Years Lived with Disability (YLDs) in Italy in 2000 and 2019 – Males 9
Figure S7b. Leading causes of age-standardised Years Lived with Disability (YLDs) in Italy in 2000 and 2019 – Females 10
Figure S8. Ten leading causes of age-standardised Years Lived with Disability (YLDs) in 2019 in Italy and in the five macro-regions 11
Table S1. Ten leading causes of all-age Years Lived with Disability (YLDs) in 2019 by region 12
Table S2. Ten leading causes of age-standardised Years Lived with Disability (YLDs) in 2019 by region 20
Figure S9. Trends of all-age Years of Life Lost (YLLs) by region in Italy between 2000 and 2021 for females (a) and males (b) 28
Figure S10. Leading causes of all-age Years of Life Lost (YLLs) in Italy in 2000 and 2019 – Both sexes 29
Figure S11a. Leading causes of all-age Years of Life Lost (YLLs) in Italy in 2000 and 2019 – Males 30
Figure S11b. Leading causes of all-age Years of Life Lost (YLLs) in Italy in 2000 and 2019 – Females 31
Figure S12. Trends of age-standardised Years of Life Lost (YLLs) by region in Italy between 2000 and 2021 for females (a) and males (b) 32
Figure S13. Leading causes of age-standardised Years of Life Lost (YLLs) in Italy in 2000 and 2019 – Both sexes 33
Figure S14a. Leading causes of age-standardised Years of Life Lost (YLLs) in Italy in 2000 and 2019 – Males 34
Figure S14b. Leading causes of age-standardised Years of Life Lost (YLLs) in Italy in 2000 and 2019 – Females 35
Figure S15. Ten leading causes of age-standardised Years of Life Lost (YLLs) in 2019 in Italy and in the five macro-regions 36
Table S3. Ten leading causes of all-age Years Lived with Disability (YLDs) in 2019 by region 37
Table S4. Ten leading causes of age-standardised Years Lived with Disability (YLDs) in 2019 by region 45
Figure S16. Trends of all-age Years of Life Lost (YLLs) by region in Italy between 2000 and 2021 for females (a) and males (b) 53
Figure S17. Ten leading causes of all-age Disability-Adjusted Life-Years (DALYs) in 2019 in Italy and the five macro-regions 54
Figure S18. Leading causes of all-age Disability-Adjusted Life-Years (DALYs) in Italy in 2000 and 2019 – Both sexes 55
Figure S19a. Leading causes of all-age Disability-Adjusted Life-Years (DALYs) in Italy in 2000 and 2019 – Males 56
Figure S19b. Leading causes of all-age Disability-Adjusted Life-Years (DALYs) in Italy in 2000 and 2019 – Females 57
Figure S20. Trends of all-age Years of Life Lost (YLLs) by region in Italy between 2000 and 2021 for females (a) and males (b) 58
Figure S21. Leading causes of age-standardised Disability-Adjusted Life-Years (DALYs) in Italy in 2000 and 2019 – Both sexes 59
Figure S22a. Leading causes of age-standardised Disability-Adjusted Life-Years (DALYs) in Italy in 2000 and 2019 – Males 60
Figure S22b. Leading causes of age-standardised Disability-Adjusted Life-Years (DALYs) in Italy in 2000 and 2019 – Females 61
Figure S23. Ten leading causes of age-standardised Disability-Adjusted Life-Years (DALYs) in 2019 in Italy and in the five macro-regions 62
Table S5. Ten leading causes of all-age Disability-Adjusted Life-Years (DALYs) in 2019 by region 63
Table S6. Ten leading causes of age-standardised Disability-Adjusted Life-Years (DALYs) in 2019 by region 71
Figure S24. Percentage of family health expenditure over total health expenditure in Italy by region and macro-region from 2000 to 2019 79

Figure S1. Trends of Health-Adjusted Life Expectancy at birth (HALE) by region in Italy between 2000 and 2021 for females (a) and males (b).

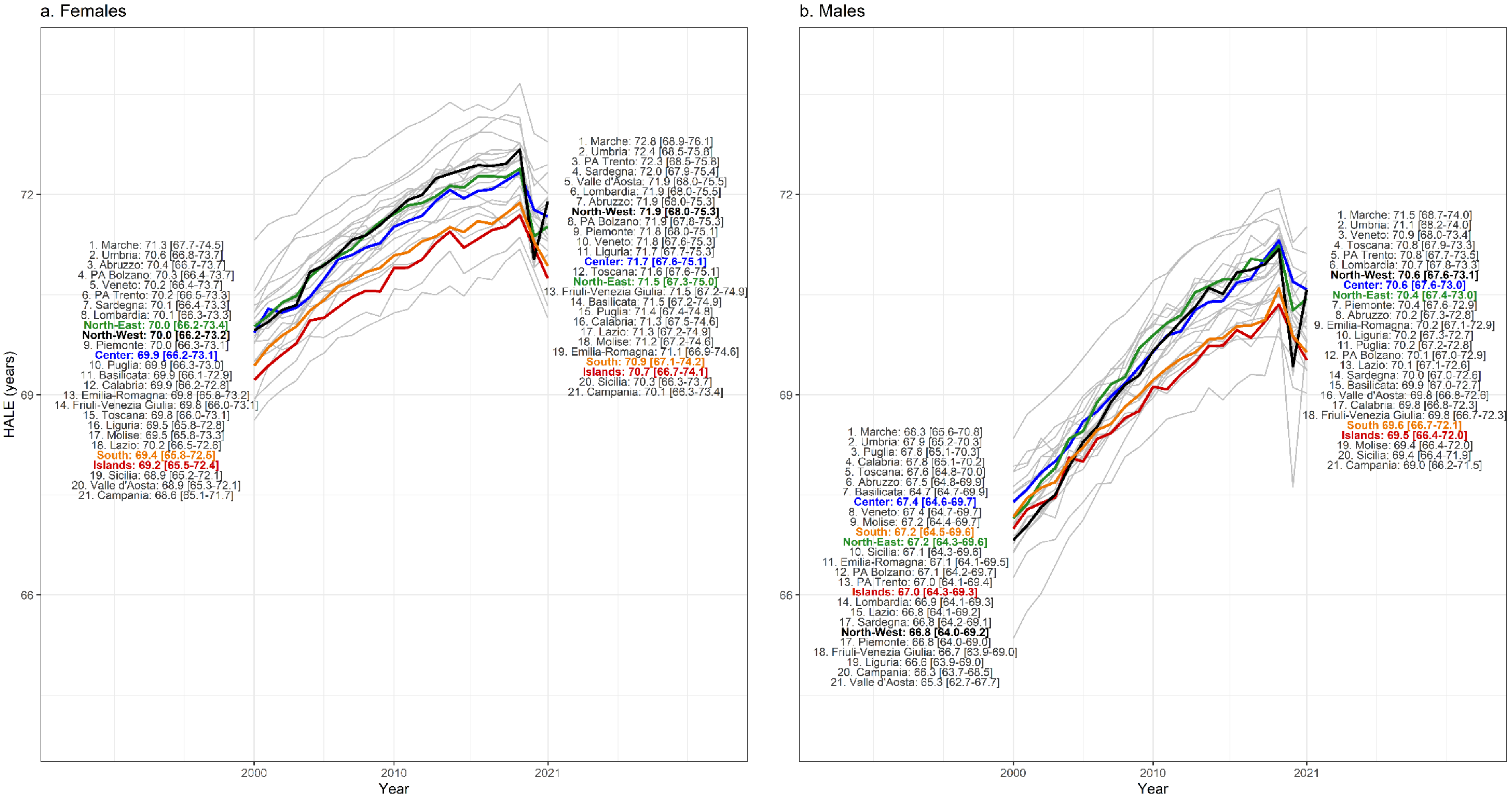

Figure S2. Trends of all-age Years Lived with Disability (YLDs) by region in Italy between 2000 and 2021 for females (a) and males (b).

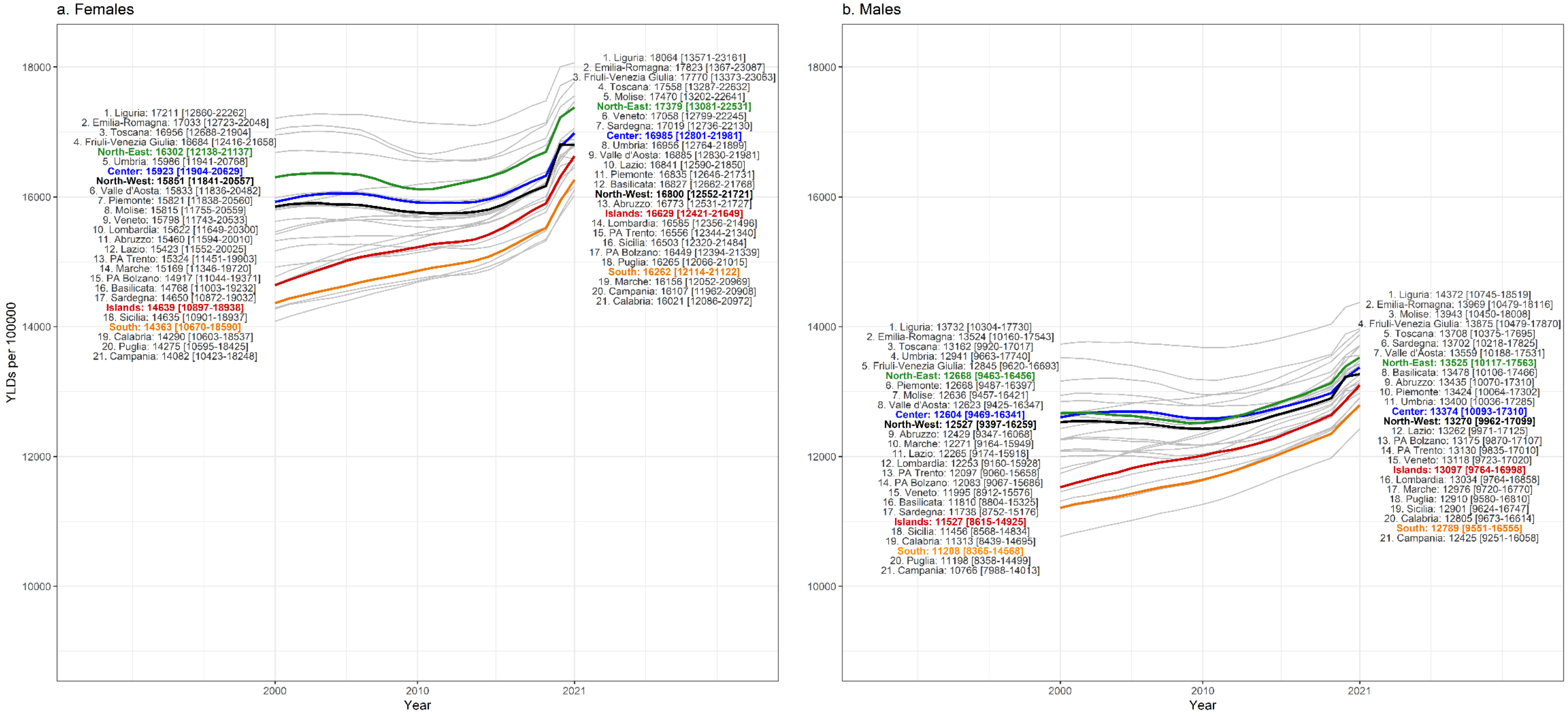

Figure S3. Leading causes of all-age Years Lived with Disability (YLDs) in Italy in 2000 and 2019 – Both sexes.

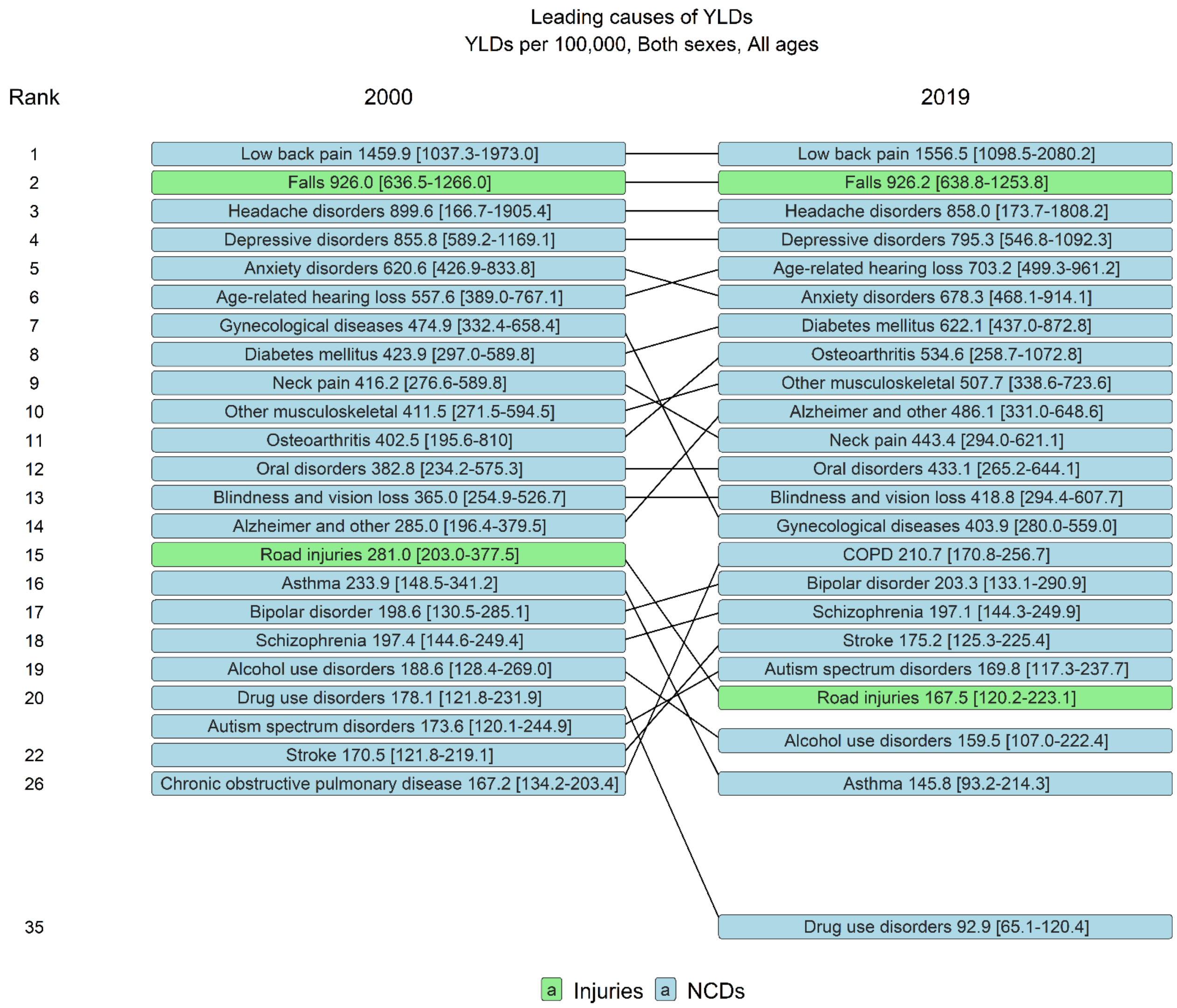

Abbreviations: COPD: Chronic Obstructive Pulmonary Disease; NCDs: Non-Communicable Diseases.

Figure S4a. Leading causes of all-age Years Lived with Disability (YLDs) in Italy in 2000 and 2019 – Males.

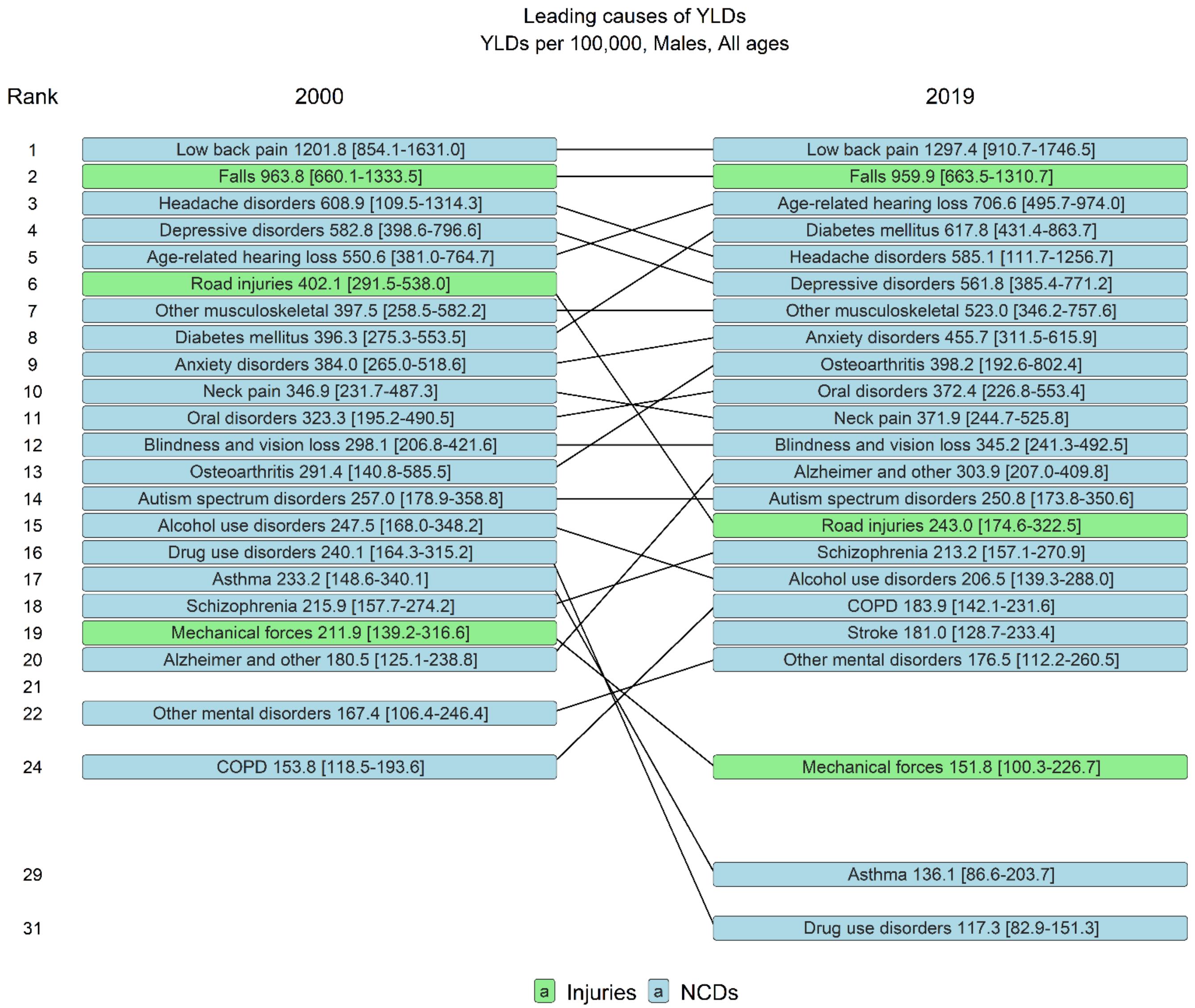

Abbreviations: COPD: Chronic Obstructive Pulmonary Disease; NCDs: Non-Communicable Diseases.

Figure S4b. Leading causes of all-age Years Lived with Disability (YLDs) in Italy in 2000 and 2019 – Females.

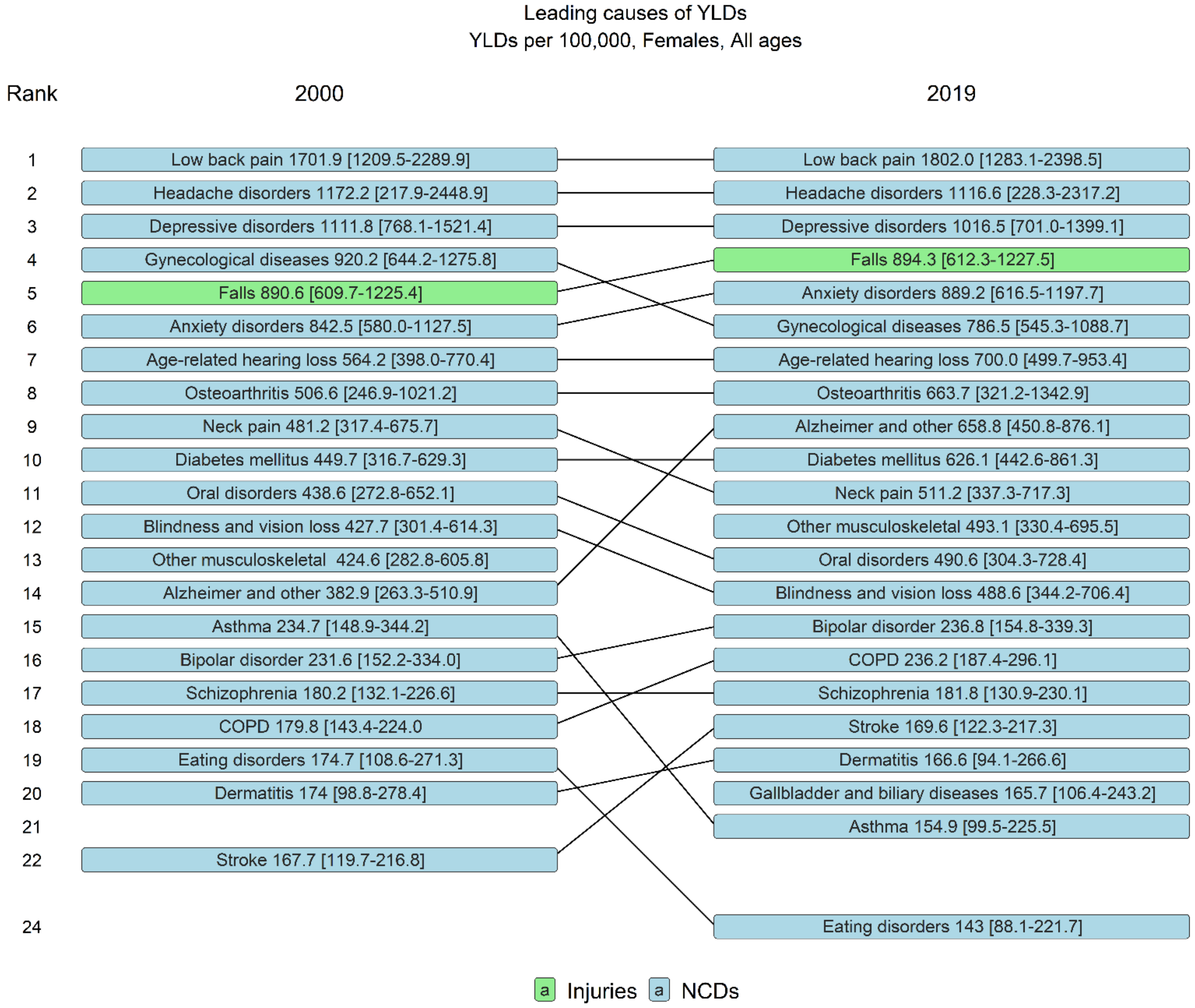

Abbreviations: COPD: Chronic Obstructive Pulmonary Disease; NCDs: Non-Communicable Diseases.

Figure S5. Trends of age-standardised Years Lived with Disability (YLDs) by region in Italy between 2000 and 2021 for females (a) and males (b).

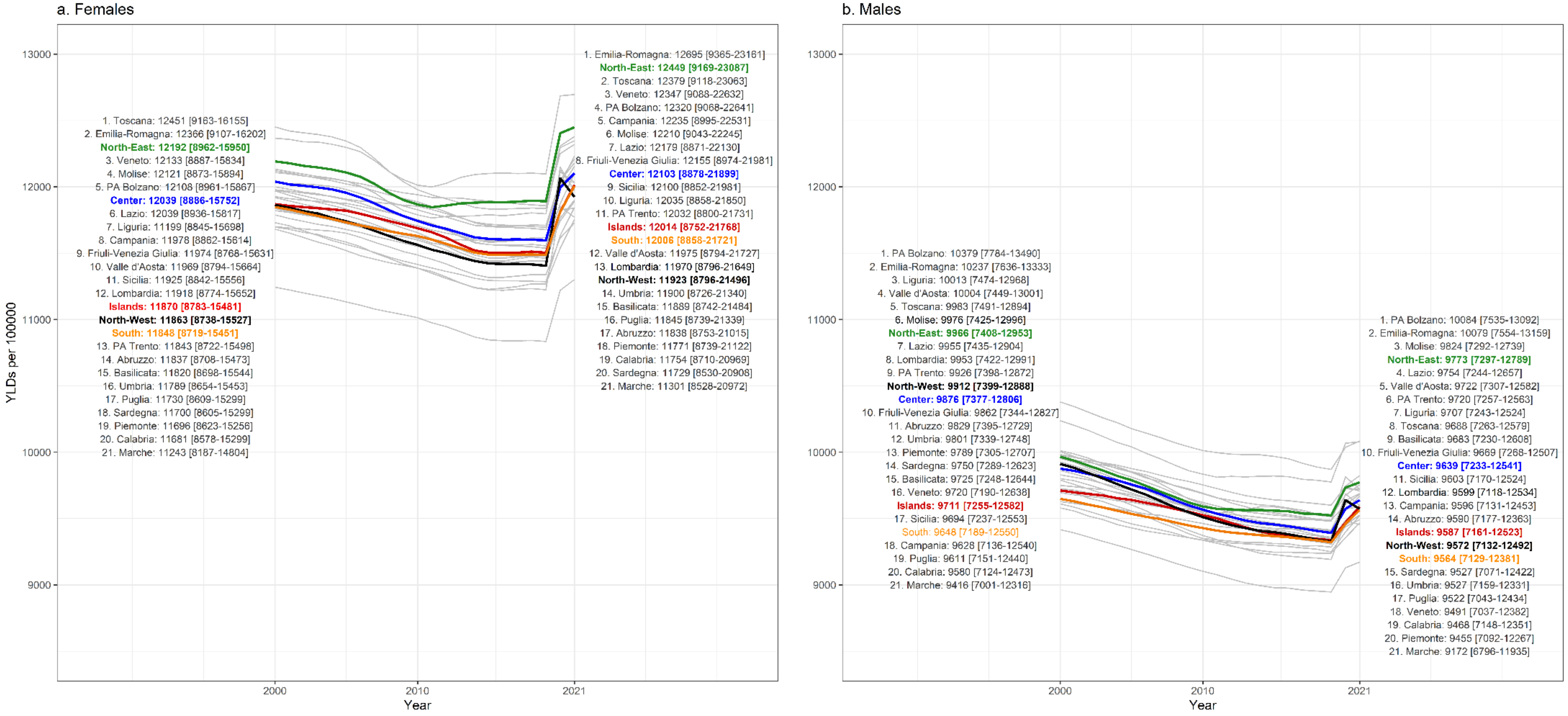

Figure S6. Leading causes of age-standardised Years Lived with Disability (YLDs) in Italy in 2000 and 2019 – Both sexes.

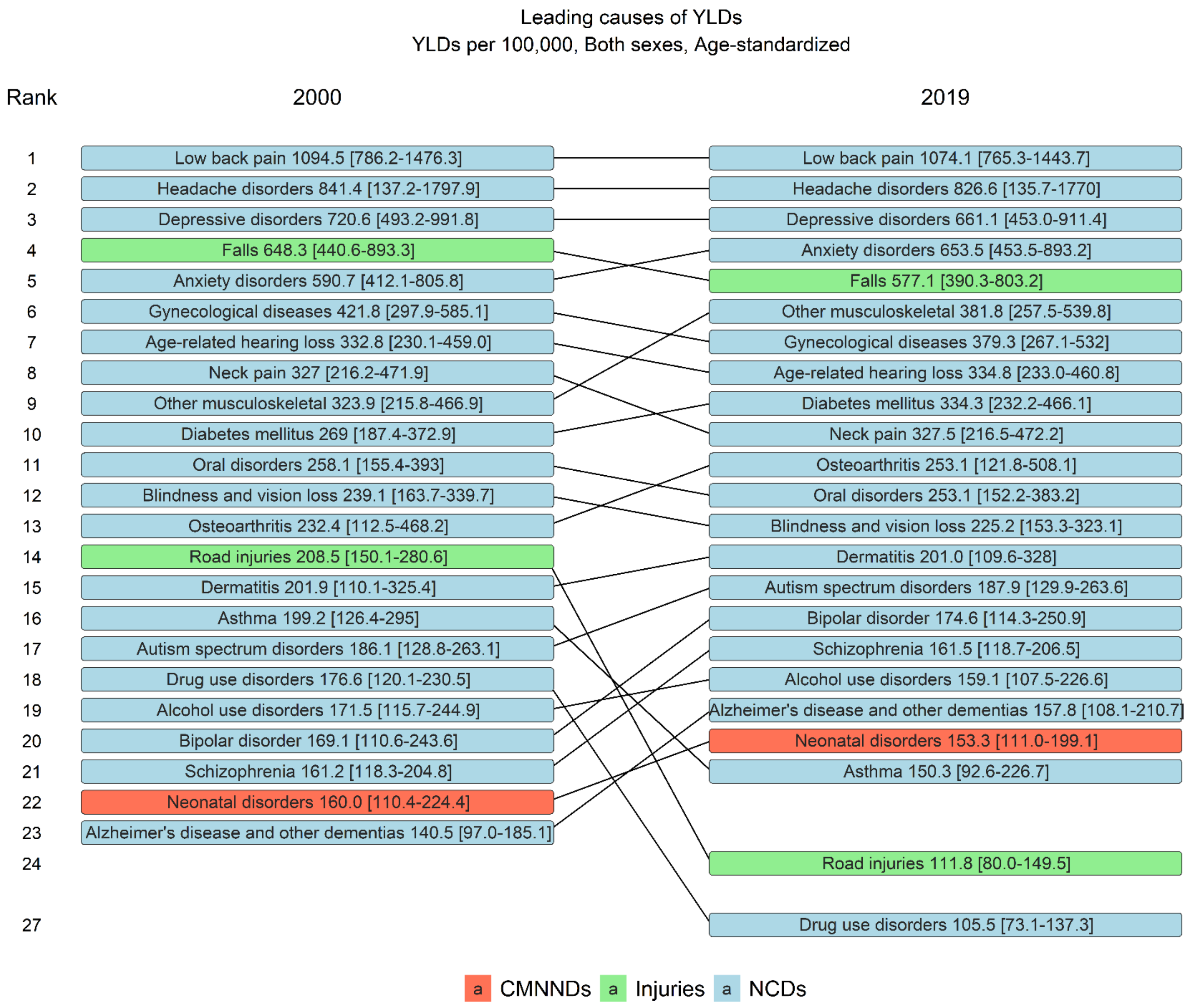

Abbreviations: CMNNDs: Communicable, Maternal, Neonatal and Nutritional Diseases; NCDs: Non-Communicable Diseases.

Figure S7a. Leading causes of age-standardised Years Lived with Disability (YLDs) in Italy in 2000 and 2019 - Males.

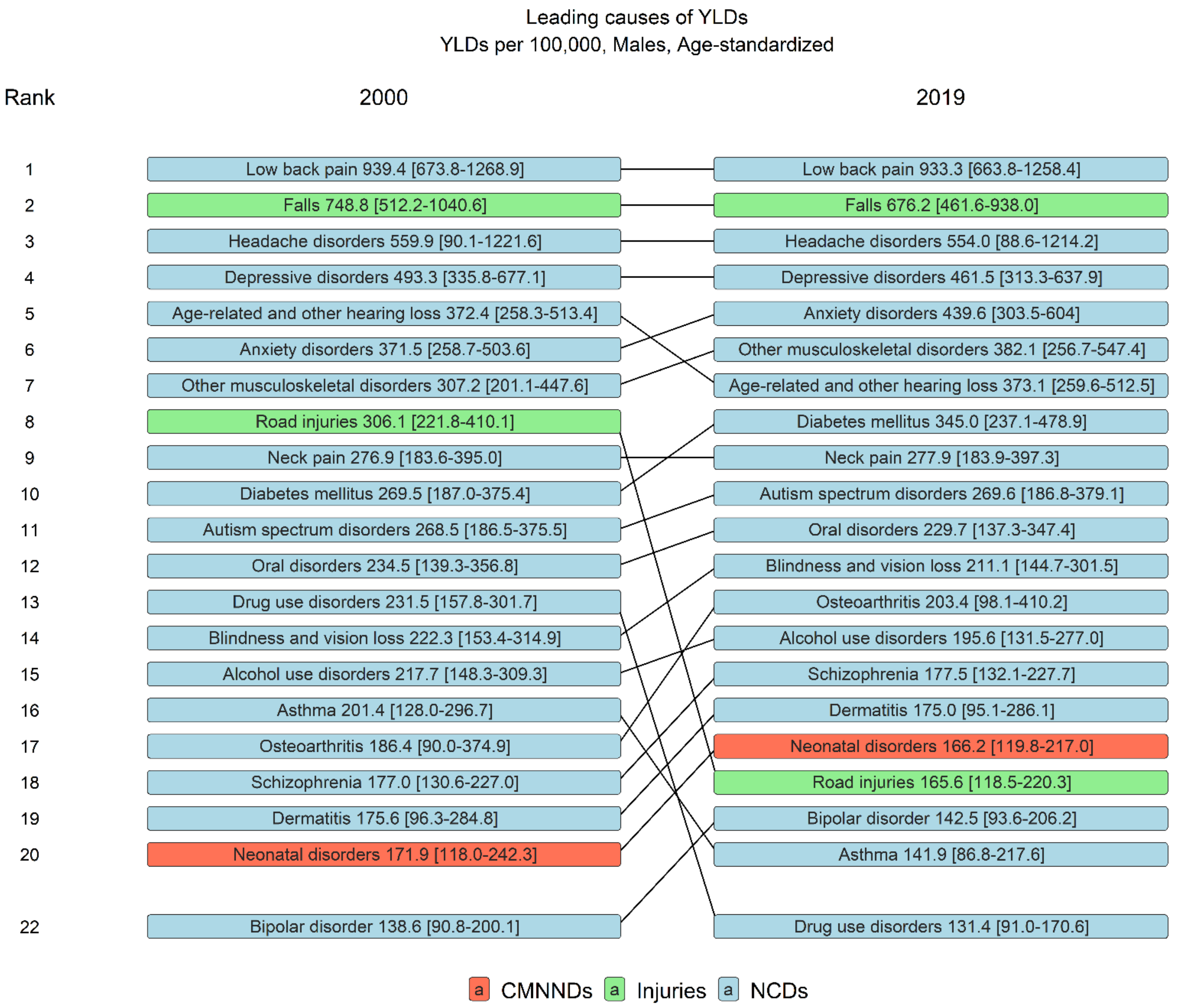

Abbreviations: CLDs: Chronic Liver Diseases; COPD: Chronic Obstructive Pulmonary Disease; CMNNDs: Communicable, Maternal, Neonatal and Nutritional Diseases; NCDs: Non-Communicable Diseases.

Figure S7b. Leading causes of age-standardised Years Lived with Disability (YLDs) in Italy in 2000 and 2019 - Females.

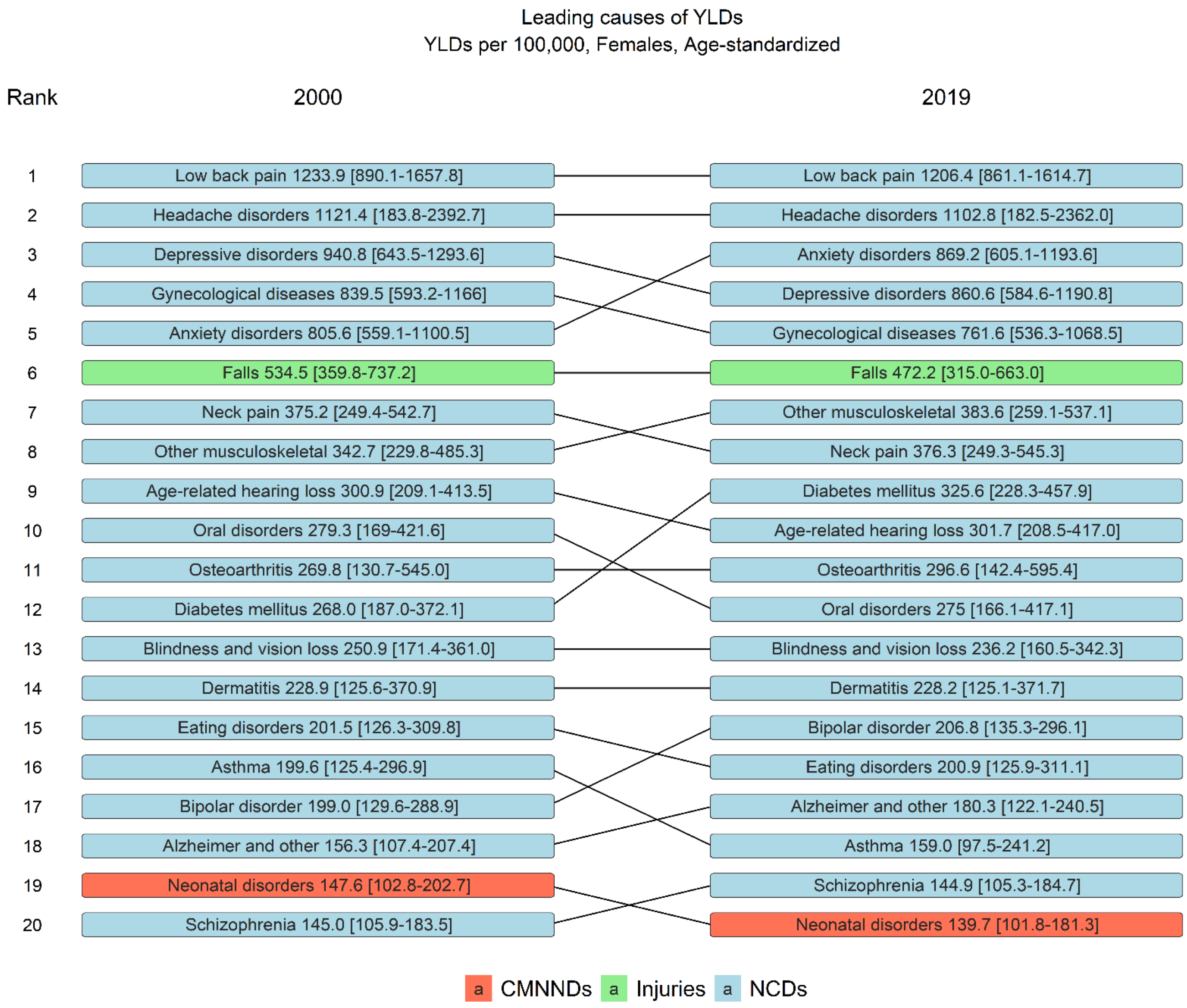

Abbreviations: CLDs: Chronic Liver Diseases; COPD: Chronic Obstructive Pulmonary Disease; CMNNDs: Communicable, Maternal, Neonatal and Nutritional Diseases; NCDs: Non-Communicable Diseases.

Figure S8. Ten leading causes of age-standardised Years Lived with Disability (YLDs) in 2019 in Italy and in the five macro-regions. Rate are expressed per 100,000 people.

Italy				
Rank 2019	Cause	YLD rate (95% UI)	Change from 2000 to 2019	Change from 2019 to 2021
1	Low back pain	1074.1 (165.3-1443.7)	-1.9%	0.7%
2	Headache disorders	826.6 (1375.7-1770.0)	-1.8%	-4.0%
3	Depressive disorders	661.1 (453.0-911.4)	-8.3%	25.1%
4	Anxiety disorders	653.5 (453.5-893.2)	10.7%	-0.4%
5	Falls	577.1 (390.3-803.2)	-11.0%	24.3%
6	Other musculoskeletal	381.8 (257.5-539.8)	17.9%	3.5%
7	Gynecological diseases	379.3 (267.1-532.0)	-10.1%	-0.6%
8	Age-related hearing loss	334.8 (233.0-460.8)	0.6%	0.0%
9	Diabetes mellitus	334.3 (232.2-466.1)	24.3%	-0.2%
10	Neck pain	327.5 (216.5-472.2)	0.2%	0.3%

North-West				
Rank 2019	Cause	YLD rate (95% UI)	Change from 2000 to 2019	Change from 2019 to 2021
1	Low back pain	1083.2 (774.3-1457.5)	-1.8%	0.7%
2	Headache disorders	841.8 (139.5-1812.5)	-1.7%	-4.0%
3	Anxiety disorders	657.4 (456.3-895.6)	11.4%	25.1%
4	Falls	616.9 (416.1-861.4)	-12.5%	-0.4%
5	Depressive disorders	606.7 (418.7-836.9)	-9.5%	24.3%
6	Other musculoskeletal	385.7 (261-545.2)	20.7%	3.5%
7	Gynecological diseases	348.7 (242.3-490.1)	-10.0%	-0.6%
8	Age-related hearing loss	335.4 (233.2-462)	0.9%	0.0%
9	Neck pain	327.7 (215.3-471.3)	0.3%	-0.2%
10	Osteoarthritis	254.6 (122.4-509.3)	9.0%	0.3%

North-East				
Rank 2019	Cause	YLD rate (95% UI)	Change from 2000 to 2019	Change from 2019 to 2021
1	Low back pain	1088.1 (774.9-1456.8)	-2.0%	0.7%
2	Headache disorders	840.4 (135.3-1808.8)	-1.5%	-4.6%
3	Depressive disorders	803.7 (545.5-1116.6)	-3.9%	22.1%
4	Anxiety disorders	655.7 (456.7-899.7)	11.7%	22.7%
5	Falls	620.9 (420.1-867.3)	-9.1%	-0.1%
6	Gynecological diseases	382.7 (271.3-535.4)	-7.8%	-0.5%
7	Other musculoskeletal	367.3 (247.3-524.8)	18.8%	3.6%
8	Age-related hearing loss	334.3 (232-459.8)	0.6%	-0.05%
9	Diabetes mellitus	331.4 (234.1-463.7)	31.2%	7.1%
10	Neck pain	326.9 (215.0-471.7)	0.2%	-0.2%

Center				
Rank 2019	Cause	YLD rate (95% UI)	Change from 2000 to 2019	Change from 2019 to 2021
1	Low back pain	1069.5 (764.2-1440.6)	-1.7%	1.2%
2	Headache disorders	825.6 (136.7-1768.6)	-1.8%	-4.9%
3	Depressive disorders	680.7 (463.4-934)	-10.7%	23.0%
4	Anxiety disorders	662.1 (457.4-910.5)	8.2%	24.0%
5	Falls	563.0 (383.6-774.7)	-12.9%	-0.2%
6	Gynecological diseases	389.1 (271.9-541.4)	-9.8%	-0.7%
7	Other musculoskeletal	383.3 (257.6-543.1)	17.0%	3.6%
8	Age-related hearing loss	334.6 (232.1-461.8)	0.5%	-0.1%
9	Neck pain	327.9 (216.7-471.8)	0.2%	-0.2%
10	Diabetes mellitus	308.7 (213.7-433.8)	25.7%	5.7%

South				
Rank 2019	Cause	YLD rate (95% UI)	Change from 2000 to 2019	Change from 2019 to 2021
1	Low back pain	1063.3 (758.7-1422.7)	-1.9%	0.7%
2	Headache disorders	821.1 (134.2-1755.9)	-2.1%	-4.9%
3	Anxiety disorders	658.0 (457.7-904.8)	11.0%	25.0%
4	Depressive disorders	606.6 (415.3-835.2)	-9.7%	22.5%
5	Falls	579.3 (391.0-809.0)	-4.6%	-0.3%
6	Gynecological diseases	399.1 (280.7-559.7)	-10.7%	-0.7%
7	Other musculoskeletal	380.0 (258.3-533.8)	14.8%	3.3%
8	Diabetes mellitus	364.5 (251.9-509.9)	23.8%	5.9%
9	Age-related hearing loss	335.0 (232.5-462.1)	0.3%	-0.1%
10	Neck pain	327.8 (217.2-472.6)	0.1%	-0.3%

Islands				
Rank 2019	Cause	YLD rate (95% UI)	Change from 2000 to 2019	Change from 2019 to 2021
1	Low back pain	1058.9 (750.1-1421.2)	-2.1%	0.6%
2	Headache disorders	781.3 (137.4-1652)	-2.1%	-4.9%
3	Depressive disorders	624.4 (424.2-860.2)	-8.5%	23.5%
4	Anxiety disorders	616.8 (428.4-852.1)	10.0%	26.5%
5	Diabetes mellitus	553.8 (381.6-751.5)	29.8%	5.1%
6	Falls	424.8 (291.0-583.0)	-22.0%	-0.4%
7	Other musculoskeletal	400.0 (270.7-566.9)	18.1%	3.3%
8	Gynecological diseases	383.5 (268.1-533.7)	-13.5%	-0.8%
9	Age-related hearing loss	334.1 (231.6-460.4)	0.2%	0.1%
10	Neck pain	327.4 (217.2-469.9)	0.0%	-0.3%

NCDs **CMNNDs** **Injuries**

Change 2000: YLD rate change (%) from 2000 to 2019; Change 2021: YLD rate change (%) from 2019 to 2021. Abbreviations: NCDs: Non-Communicable Diseases; CMNNDs: Communicable, Maternal, Neonatal, Nutritional Disorders.

Table S1. Ten leading causes of all-age Years Lived with Disability (YLDs) in 2019 by region.

Region	Rank (2019)	Cause	YLDs per 100000 [95% UI]	Change 2000 (%)	Change 2021 (%)
Piemonte - North-West	1	Low back pain	1646.6 [1163.5-2199]	4.5	1.9
	2	Headache disorders	842.1 [173.6-1767]	-8.2	3.2
	3	Falls	825.8 [574.7-1122.2]	-7.5	1.1
	4	Age-related hearing loss	771.3 [547-1055.6]	-5.3	6.2
	5	Depressive disorders	735.4 [511.3-1014.7]	18.4	8.7
	6	Anxiety disorders	679 [470.7-911.7]	9.5	15.4
	7	Osteoarthritis	578.8 [278.8-1173.3]	28.3	2.0
	8	Other musculoskeletal	557.8 [370.6-797.8]	26.2	4.8
	9	Alzheimer and other	555.3 [380.5-752.9]	27.3	3.1
	10	Diabetes mellitus	537.2 [373.8-763.4]	27.9	5.9
Valle d'Aosta - North-West	1	Low back pain	1603.3 [1147.1-2157.7]	5.2	2.2
	2	Falls	1052.8 [732.1-1425.9]	-3.7	1.3
	3	Headache disorders	844.4 [173.1-1760.9]	-5.8	-0.2
	4	Age-related hearing loss	730 [516.6-994.1]	-9.1	9.8
	5	Depressive disorders	726.9 [500.1-990.6]	18.5	9.7
	6	Anxiety disorders	677.4 [476.4-914]	16.9	9.9
	7	Diabetes mellitus	612.9 [428.6-851.8]	28.4	8.6
	8	Osteoarthritis	559.4 [272-1122.7]	31.1	3.1
	9	Alzheimer and other	490.9 [335.3-660.9]	15.2	2.5
	10	Other musculoskeletal	477.4 [312.5-701.4]	13.2	4.9

Region	Rank (2019)	Cause	YLDs per 100000 [95% UI]	Change 2000 (%)	Change 2021 (%)
Lombardia - North-West	1	Low back pain	1554.1 [1103-2068.8]	4.8	2.0
	2	Falls	1046.4 [714-1431.8]	-2.3	0.9
	3	Headache disorders	880.1 [173.6-1873.2]	-6.5	-2.9
	4	Depressive disorders	724.9 [499.6-992.9]	-10.0	17.1
	5	Age-related hearing loss	700.7 [500.1-963.9]	13.2	16.3
	6	Anxiety disorders	679.2 [464.7-918.9]	23.5	5.4
	7	Osteoarthritis	536.9 [258.2-1079.2]	19.9	2.7
	8	Other musculoskeletal	500.8 [332.1-728.5]	18.0	4.8
	9	Alzheimer and other	498 [338.3-669.2]	20.9	3.0
	10	Neck pain	442.6 [292.8-617.8]	8.5	0.4
AP Bolzano - North-East	1	Low back pain	1506.2 [1078.8-2010.6]	7.7	2.0
	2	Falls	1180.4 [812.8-1603.1]	2.5	1.5
	3	Headache disorders	847.6 [163.1-1794.5]	-4.5	-5.2
	4	Depressive disorders	705.2 [488.7-958.9]	-8.1	13.9
	5	Anxiety disorders	675.4 [460.9-907.4]	11.2	16.6
	6	Age-related hearing loss	636.5 [454.6-875.1]	28.7	2.5
	7	Diabetes mellitus	496.7 [349.4-694.6]	0.5	8.6
	8	Osteoarthritis	489.2 [235.9-989.6]	24.0	3.1
	9	Gynaecological diseases	428.7 [299-602.4]	20.5	1.1
	10	Alzheimer and other	423.6 [292.6-560.1]	21.3	0.4
AP Trento – North-East	1	Low back pain	1546.6 [1102.6-2076.4]	5.4	2.3
	2	Falls	1189.7 [814.9-1627.4]	10.0	0.9
	3	Headache disorders	845.7 [170-1790.1]	-4.8	-2.3
	4	Depressive disorders	719.1 [496.3-980.5]	-8.8	11.7
	5	Age-related hearing loss	694.5 [493.5-948]	13.4	12.6
	6	Anxiety disorders	677.4 [472.3-919]	21.8	4.3
	7	Diabetes mellitus	561.3 [395-774.7]	26.1	8.3
	8	Osteoarthritis	527.7 [257.2-1065.4]	27.5	2.7
	9	Alzheimer and other	482.9 [331.8-649.4]	20.5	0.8
	10	Neck pain	434.7 [288-608.8]	10.3	4.7

Region	Rank (2019)	Cause	YLDs per 100000 [95% UI]	Change 2000 (%)	Change 2021 (%)
Veneto - North East	1	Low back pain	1572 [1108.9-2101.9]	5.7	2.4
	2	Depressive disorders	914.7 [614.7-1263.4]	-4.5	15.1
	3	Falls	909 [624.5-1233.5]	-3.3	1.2
	4	Headache disorders	851.7 [171.1-1791.2]	-5.7	-5.2
	5	Age-related hearing loss	703.3 [495.3-956.6]	14.3	13.3
	6	Anxiety disorders	679.1 [471.5-916.1]	21.6	6.2
	7	Diabetes mellitus	637.2 [436.5-873.2]	29.6	10.7
	8	Osteoarthritis	525.1 [254.1-1068.3]	24.6	3.2
	9	Other musculoskeletal	499.2 [326-714.6]	22.8	5.3
	10	Alzheimer and other	464.1 [314.1-629.1]	14.3	3.4
Friuli-Venezia Giulia - North-East	1	Low back pain	1644.8 [1184.8-2220.3]	4.1	1.8
	2	Falls	989.2 [689.1-1333.2]	-3.2	1.5
	3	Osteoarthritis	871.4 [424.7-1750]	-2.0	2.2
	4	Headache disorders	835.1 [172.9-1747.3]	2.4	3.0
	5	Age-related hearing loss	779.5 [557-1064.5]	13.3	4.1
	6	Depressive disorders	734.1 [503.8-1005.6]	14.6	8.4
	7	Anxiety disorders	676.2 [469.1-912.1]	9.5	17.1
	8	Diabetes mellitus	656.3 [460-890.7]	40.4	9.6
	9	Alzheimer and other	556.7 [381.8-744.5]	25.6	2.5
	10	Other musculoskeletal	517.7 [339.6-757]	18.2	4.9
Liguria - North-West	1	Low back pain	1684.4 [1189.4-2251.3]	2.3	1.6
	2	Falls	1235.3 [853.1-1676.2]	-1.4	0.9
	3	Age-related hearing loss	850.3 [609.9-1156.8]	-2.0	1.3
	4	Headache disorders	826.1 [172.4-1726.2]	-0.1	4.2
	5	Depressive disorders	743.7 [517.2-1016.2]	3.7	8.1
	6	Anxiety disorders	676.6 [459-904.6]	9.3	15.9
	7	Osteoarthritis	635.8 [310-1288.5]	21.9	4.1
	8	Alzheimer and other	617.9 [424.6-825.9]	30.5	5.0
	9	Diabetes mellitus	613.2 [420.3-856.5]	33.2	3.1
	10	Oral disorders	492.1 [306.5-730]	8.3	1.3

Region	Rank (2019)	Cause	YLDs per 100000 [95% UI]	Change 2000 (%)	Change 2021 (%)
Emilia-Romagna - North-East	1	Low back pain	1596.4 [1130.8-2128]	0.5	1.8
	2	Depressive disorders	1140.7 [785.2-1551.9]	-4.0	17.8
	3	Falls	1039.5 [715.4-1412.9]	-5.0	0.9
	4	Headache disorders	896.6 [174.6-1913.8]	-4.5	-3.8
	5	Age-related hearing loss	729.5 [520.6-997.5]	12.2	11.0
	6	Anxiety disorders	675.9 [468.2-908.4]	10.3	9.3
	7	Diabetes mellitus	625.9 [444.5-859.4]	34.3	7.1
	8	Alzheimer and other	611.4 [418.3-806.4]	31.6	1.0
	9	Osteoarthritis	552.1 [268-1108.5]	23.8	2.1
	10	Other musculoskeletal	480.2 [315.1-689.2]	10.0	5.2
Toscana - Centre	1	Low back pain	1616.3 [1144-2163.9]	2.8	2.0
	2	Depressive disorders	1011 [690.3-1381.7]	-12.0	15.7
	3	Headache disorders	843.1 [174.2-1769.2]	-6.2	8.9
	4	Falls	819 [564.7-1106.9]	-7.2	1.5
	5	Anxiety disorders	784.2 [551.2-1062.4]	2.4	2.1
	6	Age-related hearing loss	770.1 [544.9-1048.7]	18.0	1.8
	7	Diabetes mellitus	613.5 [426.5-847.5]	36.2	7.2
	8	Osteoarthritis	546.2 [263.2-1112.3]	21.7	4.3
	9	Other musculoskeletal	543.8 [363.2-779.3]	21.3	2.9
	10	Alzheimer and other	525.9 [362.4-705.1]	20.4	2.3
Umbria - Centre	1	Low back pain	1611.3 [1146.2-2152.2]	2.2	2.2
	2	Falls	1025.5 [711.6-1395.2]	-0.5	1.0
	3	Headache disorders	848.3 [173.6-1777.4]	-3.4	0.3
	4	Age-related hearing loss	762.1 [542-1037.1]	-6.0	5.8
	5	Depressive disorders	735.8 [513-1006]	12.7	9.6
	6	Anxiety disorders	681.4 [477.2-906.3]	10.1	14.1
	7	Osteoarthritis	564.9 [274.1-1146.7]	21.7	2.5
	8	Alzheimer and other	551.1 [372.8-732.7]	21.1	1.6
	9	Other musculoskeletal	492.4 [328.1-703.9]	13.6	5.9
	10	Diabetes mellitus	490.6 [337.9-691.5]	15.3	5.7

Region	Rank (2019)	Cause	YLDs per 100000 [95% UI]	Change 2000 (%)	Change 2021 (%)
Marche - Centre	1	Low back pain	1455.1 [1012.9-2035.1]	4.1	5.5
	2	Headache disorders	852.2 [174.7-1785.1]	-3.8	0.4
	3	Falls	829.9 [572.8-1126.3]	-3.9	1.4
	4	Age-related hearing loss	755.5 [540.3-1030.2]	-6.4	7.2
	5	Depressive disorders	736.2 [509.4-1011.5]	16.2	4.4
	6	Osteoarthritis	531.5 [255.4-1102.1]	18.8	6.2
	7	Other musculoskeletal	530.4 [344-770.4]	19.9	5.4
	8	Alzheimer and other	524.2 [355.5-707.6]	20.0	3.9
	9	Anxiety disorders	469 [317.5-646.4]	9.5	14.4
	10	Diabetes mellitus	468.5 [329.4-663.4]	12.1	7.5
Lazio - Centre	1	Low back pain	1560 [1114.7-2087.1]	6.2	2.5
	2	Falls	1044.9 [722.5-1421]	1.0	1.6
	3	Headache disorders	862.9 [174.8-1815.8]	-5.3	0.9
	4	Depressive disorders	735.5 [503.7-1016.3]	-9.2	12.2
	5	Anxiety disorders	683.2 [470.2-921]	9.7	19.6
	6	Age-related hearing loss	678 [481-929]	26.2	2.8
	7	Diabetes mellitus	615.8 [429.6-848.7]	16.3	8.0
	8	Osteoarthritis	527.7 [257.1-1068.8]	24.9	3.1
	9	Other musculoskeletal	483.1 [322.7-702.2]	15.2	5.5
	10	Blindness and vision loss	479.7 [340.2-679.1]	17.4	2.5
Abruzzo - South	1	Low back pain	1581.8 [1124.9-2106]	6.8	2.4
	2	Falls	1024.2 [708.9-1394.5]	-3.8	1.4
	3	Headache disorders	804.9 [158.6-1705.1]	-5.3	3.2
	4	Depressive disorders	734.2 [509.7-1017.5]	-7.5	6.7
	5	Age-related hearing loss	729.5 [521.6-999.8]	17.6	5.0
	6	Anxiety disorders	681.6 [474.4-915.2]	15.5	9.4
	7	Diabetes mellitus	639.5 [441.3-877.8]	30.5	8.2
	8	Osteoarthritis	550.9 [265.5-1104.3]	30.4	3.0
	9	Alzheimer and other	507.3 [348.7-672.8]	21.2	3.7
	10	Other musculoskeletal	499.9 [332.8-727.4]	20.0	4.4

Region	Rank (2019)	Cause	YLDs per 100000 [95% UI]	Change 2000 (%)	Change 2021 (%)
Molise - South	1	Low back pain	1590.7 [1128.4-2109.6]	7.9	2.2
	2	Falls	1119.2 [775.5-1532.8]	1.5	1.3
	3	Headache disorders	851.5 [173.1-1778.6]	-2.9	1.7
	4	Age-related hearing loss	753.8 [535.1-1027]	-4.3	8.2
	5	Depressive disorders	736.7 [514.5-1010.5]	18.9	9.4
	6	Diabetes mellitus	717.9 [499.6-984.5]	19.1	8.3
	7	Anxiety disorders	681.7 [481.3-912.9]	43.8	13.3
	8	Osteoarthritis	555.1 [266.8-1116.7]	18.8	3.1
	9	Alzheimer and other	532.7 [361.4-718.5]	26.2	3.0
	10	Other musculoskeletal	466.1 [303.5-675]	12.0	5.5
Campania - South	1	Low back pain	1462.1 [1028.8-1959.4]	12.6	2.7
	2	Headache disorders	877.7 [171.2-1837.2]	-2.3	-2.8
	3	Falls	817.9 [555.8-1116.7]	8.0	3.1
	4	Depressive disorders	718.3 [492.1-986.2]	8.0	15.8
	5	Anxiety disorders	691.4 [480.7-931.6]	11.2	20.1
	6	Diabetes mellitus	663.4 [463.6-920.5]	29.8	7.6
	7	Age-related hearing loss	601.1 [423.1-827.4]	34.7	3.2
	8	Other musculoskeletal	513.2 [345.9-734.1]	24.6	5.1
	9	Gynaecological diseases	466.4 [323-651.3]	15.4	1.5
	10	Osteoarthritis	455.7 [218.1-916.3]	19.2	1.1
Puglia - South	1	Low back pain	1517.3 [1073.6-2035.8]	12.4	2.4
	2	Falls	913.6 [626.1-1246.9]	1.1	1.9
	3	Headache disorders	868.4 [172.4-1829.5]	5.2	0.0
	4	Depressive disorders	731.4 [506.2-996.9]	-5.6	14.2
	5	Anxiety disorders	688.1 [479.2-923.6]	10.1	19.7
	6	Age-related hearing loss	676 [475.8-932.9]	38.3	3.5
	7	Diabetes mellitus	591.6 [411.5-839.6]	21.6	11.1
	8	Osteoarthritis	505.3 [246.3-1021.9]	25.7	3.6
	9	Other musculoskeletal	493.4 [329-701.4]	24.8	5.5
	10	Neck pain	440.6 [293.4-616.8]	13.3	1.3

Region	Rank (2019)	Cause	YLDs per 100000 [95% UI]	Change 2000 (%)	Change 2021 (%)
Basilicata - South	1	Low back pain	1569.5 [1114.3-2089.8]	11.4	2.6
	2	Falls	860.4 [596.8-1170.3]	-3.3	2.0
	3	Headache disorders	860.4 [178.2-1815]	10.3	-3.4
	4	Depressive disorders	735.5 [505-1019.6]	-5.5	10.8
	5	Age-related hearing loss	717.7 [509.8-982]	15.8	11.3
	6	Diabetes mellitus	715.6 [499.8-990]	33.0	8.7
	7	Anxiety disorders	682.9 [469.4-923.2]	47.3	8.1
	8	Osteoarthritis	532.5 [257.1-1081.5]	18.9	3.7
	9	Other musculoskeletal	492.3 [325-699.9]	22.7	5.5
	10	Alzheimer and other	489.2 [334-655.1]	22.8	3.5
Calabria - South	1	Low back pain	1496.3 [1051.1-2009.7]	10.8	2.4
	2	Headache disorders	867 [171.3-1826.4]	-3.0	-1.0
	3	Falls	844.5 [580.8-1149.3]	3.4	0.2
	4	Depressive disorders	728.1 [507.8-989.5]	-5.5	12.8
	5	Diabetes mellitus	716.5 [503.3-987.7]	14.6	14.3
	6	Anxiety disorders	687.9 [467.7-922.8]	35.0	13.0
	7	Age-related hearing loss	673.4 [472.4-924.5]	43.7	2.5
	8	Other musculoskeletal	511.3 [332.1-735.4]	17.1	4.8
	9	Osteoarthritis	501.2 [243.5-1009.4]	27.2	3.1
	10	Neck pain	437.2 [290-616.9]	12.1	1.4
Sicilia - Islands	1	Low back pain	1484.4 [1048.1-1999.7]	9.7	2.4
	2	Diabetes mellitus	1065.9 [729.8-1462.4]	29.1	6.8
	3	Headache disorders	804.8 [171.6-1689]	4.6	7.9
	4	Depressive disorders	722.5 [496.5-993.3]	1.7	16.6
	5	Anxiety disorders	685.8 [478.3-928.6]	6.3	11.5
	6	Age-related hearing loss	652.2 [460.9-893.2]	4.8	3.0
	7	Falls	643.7 [442.5-874.2]	27.2	1.6
	8	Other musculoskeletal	540 [365.1-763.9]	9.5	5.1
	9	Osteoarthritis	486.8 [235.9-986.2]	18.2	3.3
	10	Alzheimer and other	467.6 [322.6-624]	18.5	3.4

Region	Rank (2019)	Cause	YLDs per 100000 [95% UI]	Change 2000 (%)	Change 2021 (%)
Sardegna - Islands	1	Low back pain	1610 [1133.2-2156.1]	14.5	2.7
	2	Diabetes mellitus	887.2 [618.3-1230.1]	-4.2	12.2
	3	Headache disorders	862.7 [178.3-1812.6]	2.7	9.6
	4	Depressive disorders	839.2 [574.5-1151.2]	7.2	1.7
	5	Falls	833.5 [577-1134]	54.4	-2.2
	6	Age-related hearing loss	726 [515.8-991.8]	43.9	4.0
	7	Osteoarthritis	553.9 [267.3-1109.3]	12.4	5.7
	8	Other musculoskeletal	531.9 [352.1-757.3]	10.1	8.3
	9	Anxiety disorders	510 [345-698.3]	21.9	9.8
	10	Alzheimer and other	479.8 [325.7-643.7]	17.1	5.2

Change 2000: YLL rate change (%) from 2000 to 2019; Change 2021: YLL rate change (%) from 2019 to 2021.
Abbreviations: UI: Uncertainty Interval.

Table S2. Ten leading causes of age-standardised Years Lived with Disability (YLDs) in 2019 by region.

Region	Rank (2019)	Cause	YLDs per 100000 [95% UI]	Change 2000 (%)	Change 2021 (%)
Piemonte - North-West	1	Low back pain	1101.6 [776-1471.5]	-1.4	0.6
	2	Headache disorders	826.5 [135.8-1773.9]	-1.5	0.6
	3	Anxiety disorders	658.3 [453-896.4]	-1.9	19.4
	4	Depressive disorders	608 [414.2-845.3]	3.0	25.6
	5	Falls	469.8 [323.3-645.1]	-19.9	-0.2
	6	Other musculoskeletal	411.3 [279.5-579.8]	10.4	3.6
	7	Age-related hearing loss	336.6 [233.5-467]	0.9	-0.2
	8	Gynaecological diseases	333.9 [230.5-470.2]	0.6	-0.6
	9	Neck pain	328.3 [215.3-472.9]	0.4	-0.2
	10	Diabetes mellitus	270.4 [187-380.5]	9.2	5.1
Valle d'Aosta - North-West	1	Low back pain	1091 [784.4-1460.6]	-1.8	0.6
	2	Headache disorders	825.6 [136.1-1771]	-0.9	-2.8
	3	Anxiety disorders	657.5 [452-903.1]	-11.0	19.5
	4	Falls	634.2 [434.8-869.3]	-4.8	16.5
	5	Depressive disorders	606.3 [414.8-828.4]	3.4	4.4
	6	Other musculoskeletal	364.1 [242.2-518.2]	-13.5	3.1
	7	Gynaecological diseases	355.8 [249.9-500.7]	6.6	-0.3
	8	Age-related hearing loss	335.8 [232.6-463.8]	3.2	2.4
	9	Neck pain	327.4 [215.4-470.9]	9.7	2.4
	10	Diabetes mellitus	323.2 [223.6-446.4]	23.8	1.1

Region	Rank (2019)	Cause	YLDs per 100000 [95% UI]	Change 2000 (%)	Change 2021 (%)
Lombardia - North-West	1	Low back pain	1075.2 [764-1447.2]	-2.0	0.7
	2	Headache disorders	850.9 [142.5-1830.1]	-1.9	-3.5
	3	Falls	675.8 [454-952.8]	-10.5	21.1
	4	Anxiety disorders	656.9 [454.1-893]	-2.0	14.3
	5	Depressive disorders	606.2 [418.5-832.5]	2.7	11.0
	6	Other musculoskeletal	378.1 [253.7-536.3]	-2.1	3.4
	7	Gynaecological diseases	350.4 [243.8-489]	5.7	-0.6
	8	Age-related hearing loss	335.1 [232.5-463.2]	2.6	0.1
	9	Neck pain	327.4 [215.2-469.9]	3.8	-0.2
	10	Osteoarthritis	254.6 [122.2-509.3]	-1.4	0.5
AP Bolzano - North-East	1	Low back pain	1096.3 [789.9-1470.3]	-1.8	0.5
	2	Headache disorders	819.7 [134.9-1765.3]	-7.7	-2.6
	3	Falls	797.3 [541.7-1104.5]	-4.3	-1.5
	4	Anxiety disorders	655.1 [455.9-889.2]	-1.8	19.0
	5	Depressive disorders	602.8 [410.6-825.7]	2.8	17.9
	6	Gynaecological diseases	405.6 [284.8-570.6]	-7.2	-0.7
	7	Age-related hearing loss	333.7 [232.5-461.8]	0.7	0.1
	8	Neck pain	325.5 [213.8-469.1]	0.1	-0.2
	9	Other musculoskeletal	308.2 [197-447]	19.4	4.2
	10	Diabetes mellitus	295.7 [206.1-411.7]	15.5	6.3
AP Trento - North-East	1	Low back pain	1087.7 [782.7-1465.1]	-1.9	0.8
	2	Headache disorders	823.9 [137.5-1767.9]	-1.4	-4.0
	3	Falls	790.8 [534.1-1104.6]	2.2	-0.9
	4	Anxiety disorders	656.3 [459.8-894.6]	-2.0	19.0
	5	Depressive disorders	605.8 [408.9-833.7]	2.8	19.6
	6	Gynaecological diseases	377.9 [266.6-534.8]	-4.4	-0.2
	7	Age-related hearing loss	335 [231.9-459.8]	0.5	4.2
	8	Other musculoskeletal	335 [221.5-485.2]	2.7	0.0
	9	Neck pain	326.8 [215.4-469.2]	11.9	1.6
	10	Diabetes mellitus	312.5 [218.2-437.9]	23.0	4.4

Region	Rank (2019)	Cause	YLDs per 100000 [95% UI]	Change 2000 (%)	Change 2021 (%)
Veneto - North East	1	Low back pain	1083.3 [767.5-1452.2]	-2.1	0.8
	2	Headache disorders	823.5 [135.7-1760.2]	-1.5	11.2
	3	Depressive disorders	762.4 [518-1063]	-4.2	4.4
	4	Anxiety disorders	656 [456.8-895.5]	0.2	19.3
	5	Falls	576.2 [389.9-802.5]	-1.8	-0.5
	6	Gynaecological diseases	396.5 [279.7-556.6]	-6.9	-0.4
	7	Other musculoskeletal	374.5 [249.5-533.9]	12.4	3.6
	8	Diabetes mellitus	337.4 [233.5-470.7]	3.3	8.3
	9	Age-related hearing loss	335.8 [231.8-463.8]	6.6	-0.2
	10	Neck pain	327.2 [216.9-471.2]	26.2	-0.3
Friuli-Venezia Giulia - North-East	1	Low back pain	1087.5 [773-1465.5]	-1.8	0.6
	2	Headache disorders	822.6 [136.5-1768.3]	-1.5	0.6
	3	Anxiety disorders	656.3 [453.5-901.6]	-1.5	19.2
	4	Depressive disorders	604.8 [413.6-829.7]	-5.4	24.8
	5	Falls	567.9 [389.4-779.3]	-3.5	0.0
	6	Other musculoskeletal	385.5 [262.3-544.4]	-1.0	3.4
	7	Osteoarthritis	382.7 [184.7-766.9]	8.4	-0.1
	8	Gynaecological diseases	350 [245.5-492.9]	5.6	1.0
	9	Age-related hearing loss	333.9 [232.4-462.4]	2.4	4.3
	10	Diabetes mellitus	328.1 [228.5-454.1]	0.6	1.7
Liguria - North-West	1	Low back pain	1082.7 [781.1-1445.1]	-1.7	0.6
	2	Headache disorders	825.9 [134.9-1771.6]	-1.6	-0.5
	3	Anxiety disorders	658 [452.7-886.3]	-10.0	19.3
	4	Falls	637.7 [435-876.2]	-4.9	17.3
	5	Depressive disorders	606.6 [414.6-831.5]	2.6	4.7
	6	Gynaecological diseases	380.6 [269.8-534.5]	-12.6	-0.4
	7	Other musculoskeletal	365.4 [244.1-517.1]	10.2	3.5
	8	Age-related hearing loss	333.9 [232-459.9]	2.1	-0.1
	9	Neck pain	327.5 [215.9-470.7]	5.0	-0.3
	10	Diabetes mellitus	287.9 [197.8-408.5]	11.0	6.9

Region	Rank (2019)	Cause	YLDs per 100000 [95% UI]	Change 2000 (%)	Change 2021 (%)
Emilia-Romagna - North-East	1	Low back pain	1092.8 [780.2-1474]	-1.9	7.6
	2	Depressive disorders	950 [644.9-1304.4]	-2.7	15.7
	3	Headache disorders	868.1 [136.1-1879.7]	-1.8	-4.0
	4	Anxiety disorders	655.1 [451.8-892.7]	-5.7	24.3
	5	Falls	642.3 [433.2-899.7]	9.4	0.1
	6	Gynaecological diseases	373.7 [265-524.9]	-8.7	1.1
	7	Other musculoskeletal	365 [245.5-513]	10.0	1.9
	8	Age-related hearing loss	332.9 [231.4-457.7]	2.1	5.6
	9	Diabetes mellitus	332.1 [233.7-461.6]	8.5	0.3
	10	Neck pain	326.8 [214.5-471.1]	24.7	-0.1
Toscana - Centre	1	Low back pain	1082 [777.3-1443.1]	-1.6	0.6
	2	Depressive disorders	837.6 [566.4-1145.2]	-11.3	22.0
	3	Headache disorders	825.6 [134.7-1763]	-1.3	11.8
	4	Anxiety disorders	758.2 [527.8-1042.2]	3.9	3.6
	5	Falls	443.4 [303.6-606.3]	-20.3	-0.2
	6	Other musculoskeletal	405.9 [272.2-566.2]	1.1	3.5
	7	Gynaecological diseases	361.8 [251.1-507.5]	6.5	-0.8
	8	Age-related hearing loss	335.6 [232.3-464.8]	0.9	-0.3
	9	Neck pain	327.7 [215.3-466.8]	0.4	-0.1
	10	Diabetes mellitus	309.9 [214.9-431.3]	19.5	5.6
Umbria - Centre	1	Low back pain	1090.7 [781.9-1468.8]	-1.7	0.8
	2	Headache disorders	827.8 [137.2-1774.2]	-1.3	-2.0
	3	Anxiety disorders	659.1 [455.4-904.3]	-1.7	19.5
	4	Falls	613.2 [418.3-847.9]	-7.2	20.6
	5	Depressive disorders	608.8 [411.8-837.3]	3.3	0.5
	6	Gynaecological diseases	381 [265.4-532.6]	-8.2	1.1
	7	Other musculoskeletal	371.5 [251.7-528.5]	11.1	2.0
	8	Age-related hearing loss	334.4 [232.2-462.2]	2.3	0.2
	9	Neck pain	328.4 [219.7-472.3]	4.8	-0.1
	10	Diabetes mellitus	254.2 [175.5-359.6]	-1.5	4.9

Region	Rank (2019)	Cause	YLDs per 100000 [95% UI]	Change 2000 (%)	Change 2021 (%)
Marche - Centre	1	Low back pain	980.8 [680.3-1363.9]	-1.8	3.9
	2	Headache disorders	829.6 [137-1778.7]	-1.4	-4.9
	3	Depressive disorders	609.6 [418-839.7]	-9.3	22.3
	4	Falls	482 [327.5-666.3]	-13.4	18.8
	5	Anxiety disorders	456.5 [305.7-636.5]	7.9	5.6
	6	Other musculoskeletal	396.9 [265.9-565.3]	-1.4	3.7
	7	Gynaecological diseases	365.1 [256.5-511.6]	8.5	-0.5
	8	Age-related hearing loss	336.5 [234.1-465.9]	0.3	-0.1
	9	Neck pain	329.1 [219.4-475.3]	0.4	-0.3
	10	Oral disorders	255.8 [153-385.2]	-2.1	1.4
Lazio - Centre	1	Low back pain	1082.2 [773.7-1451.7]	-1.8	0.9
	2	Headache disorders	824.2 [136.9-1767.6]	-2.3	0.2
	3	Anxiety disorders	657.5 [452.2-904.7]	-10.8	19.1
	4	Falls	655.3 [444.9-907.2]	-3.7	16.2
	5	Depressive disorders	611.8 [418.8-839.7]	3.4	6.7
	6	Gynaecological diseases	413.8 [289.6-575.3]	-10.3	-0.8
	7	Other musculoskeletal	366.6 [247.5-528.2]	10.5	3.7
	8	Diabetes mellitus	334.6 [233.5-466.6]	2.2	5.8
	9	Age-related hearing loss	333.4 [231.1-459.5]	4.6	0.0
	10	Neck pain	327.5 [216.7-470]	19.8	-0.3
Abruzzo - South	1	Low back pain	1076.4 [771-1435.5]	-1.9	0.8
	2	Headache disorders	780.8 [125.3-1709.7]	-3.6	0.1
	3	Anxiety disorders	656.7 [454.3-901]	-9.1	13.5
	4	Falls	614.5 [417.6-853.3]	-8.3	16.2
	5	Depressive disorders	605.9 [411.7-834.5]	2.6	1.3
	6	Gynaecological diseases	381.7 [269.2-538.9]	-13.7	1.7
	7	Other musculoskeletal	375.9 [251.5-539.4]	12.9	0.7
	8	Diabetes mellitus	334.4 [230.3-459.6]	2.3	5.9
	9	Age-related hearing loss	334.3 [234.4-460.3]	6.9	-0.1
	10	Neck pain	327.5 [216.9-468.4]	26.3	-0.2

Region	Rank (2019)	Cause	YLDs per 100000 [95% UI]	Change 2000 (%)	Change 2021 (%)
Molise - South	1	Low back pain	1070.7 [760.9-1434.4]	-2.1	0.6
	2	Headache disorders	820 [136.4-1746.1]	-2.1	0.3
	3	Falls	667.6 [452.7-929.6]	-10.1	16.7
	4	Anxiety disorders	654.4 [454.1-903.8]	-2.0	14.4
	5	Depressive disorders	602.5 [412.7-827.4]	2.3	10.3
	6	Gynaecological diseases	379.5 [266.6-538.6]	-13.2	3.1
	7	Diabetes mellitus	369.3 [258-504.5]	-3.4	2.0
	8	Other musculoskeletal	351.9 [235-501.9]	5.6	3.7
	9	Age-related hearing loss	334.3 [233.2-459.8]	2.6	0.0
	10	Neck pain	326.5 [216.3-464.9]	11.2	-0.3
Campania - South	1	Low back pain	1065.4 [759.8-1426.4]	-1.7	0.7
	2	Headache disorders	825.2 [135.6-1758.7]	-1.8	0.6
	3	Anxiety disorders	659 [461.2-907.3]	-1.9	19.0
	4	Depressive disorders	606.2 [413.1-830.7]	2.2	23.1
	5	Falls	573.5 [386-800.9]	4.8	-0.5
	6	Gynaecological diseases	426.2 [300.6-598]	-7.8	-0.7
	7	Other musculoskeletal	389.4 [267.4-552.8]	12.1	3.6
	8	Diabetes mellitus	386.5 [268.8-539.2]	15.8	3.9
	9	Age-related hearing loss	334.8 [232.9-464]	2.3	-0.2
	10	Neck pain	327.8 [218.2-472]	6.8	-0.3
Puglia - South	1	Low back pain	1058.4 [753.2-1424]	-2.2	0.5
	2	Headache disorders	825.7 [135.7-1761]	-2.1	0.7
	3	Anxiety disorders	657.1 [457.5-894.5]	-2.5	19.5
	4	Depressive disorders	607.6 [412.4-832.5]	-5.0	24.4
	5	Falls	593.7 [402.1-833]	0.0	-0.2
	6	Gynaecological diseases	382.1 [267.7-536.4]	-12.9	0.2
	7	Other musculoskeletal	369.7 [251-516.3]	10.7	2.5
	8	Age-related hearing loss	335.4 [234-466.2]	2.2	6.2
	9	Diabetes mellitus	329.2 [225-465.3]	2.4	1.9
	10	Neck pain	327.9 [216.3-472.5]	14.8	-0.2

Region	Rank (2019)	Cause	YLDs per 100000 [95% UI]	Change 2000 (%)	Change 2021 (%)
Basilicata - South	1	Low back pain	1075.6 [767.1-1451.9]	-1.5	0.7
	2	Headache disorders	821.1 [136.9-1759.8]	-2.1	-3.4
	3	Anxiety disorders	653.6 [456.1-897]	-2.4	19.5
	4	Depressive disorders	604.8 [415.2-839.1]	2.8	17.6
	5	Falls	509.3 [349.3-699.7]	-10.3	-0.1
	6	Diabetes mellitus	378.5 [261.5-526.2]	-10.7	5.9
	7	Gynaecological diseases	374.7 [263-527.7]	12.0	2.1
	8	Other musculoskeletal	369.9 [249.9-526.7]	13.2	0.8
	9	Age-related hearing loss	334.3 [233.7-462]	3.0	0.0
	10	Neck pain	327.1 [216.4-474.1]	9.9	-0.3
Calabria - South	1	Low back pain	1054.1 [751.7-1405.7]	-2.0	0.6
	2	Headache disorders	826 [136.6-1769.8]	-1.8	-1.4
	3	Anxiety disorders	659.1 [458.9-908.5]	-1.8	19.1
	4	Depressive disorders	607.4 [416.7-838.1]	-1.2	21.0
	5	Falls	543.6 [367.8-756.3]	-8.3	-0.1
	6	Diabetes mellitus	396.1 [275.7-544.4]	-7.9	5.8
	7	Other musculoskeletal	385.3 [257-555.5]	15.1	3.0
	8	Gynaecological diseases	374.2 [261.4-527.2]	14.3	-0.7
	9	Age-related hearing loss	335.4 [231.4-465.2]	2.6	-0.1
	10	Neck pain	328 [215.5-473.8]	7.6	-0.3
Sicilia - Islands	1	Low back pain	1054.8 [742.2-1418.2]	-2.2	0.6
	2	Headache disorders	768.7 [137.7-1622.4]	-1.9	9.1
	3	Anxiety disorders	655.8 [457.7-903.6]	-2.6	16.0
	4	Depressive disorders	605.7 [410.3-834.2]	2.1	20.8
	5	Diabetes mellitus	590.3 [403.9-801.8]	10.8	4.1
	6	Other musculoskeletal	405.7 [272.8-579.7]	-10.2	3.2
	7	Falls	404.3 [277-555.5]	-9.2	-0.4
	8	Gynaecological diseases	387.3 [270.4-540.8]	13.5	-0.9
	9	Age-related hearing loss	333.9 [231.4-459.8]	0.1	0.1
	10	Neck pain	327.5 [218.1-471.5]	0.0	-0.3

Region	Rank (2019)	Cause	YLDs per 100000 [95% UI]	Change 2000 (%)	Change 2021 (%)
Sardegna - Islands	1	Low back pain	1071.6 [756.2-1429.4]	-1.9	0.6
	2	Headache disorders	821.7 [136.6-1759]	-2.2	-2.1
	3	Depressive disorders	684.1 [468.3-942.6]	-3.6	14.3
	4	Anxiety disorders	488.4 [330-673]	-15.4	19.4
	5	Falls	486.7 [333.1-670.2]	5.7	1.5
	6	Diabetes mellitus	453.8 [316-629.5]	8.4	6.7
	7	Other musculoskeletal	383.5 [264-543.3]	3.3	3.4
	8	Gynaecological diseases	371.1 [260.2-518.4]	11.2	-0.6
	9	Age-related hearing loss	334.6 [233.2-459.4]	0.9	0.0
	10	Neck pain	327.1 [216-469.8]	0.0	-0.2

Change 2000: YLL rate change (%) from 2000 to 2019; Change 2021: YLL rate change (%) from 2019 to 2021;
Abbreviations: UI: Uncertainty Interval.

Figure S9. Trends of all-age Years of Life Lost (YLLs) by region in Italy between 2000 and 2021 for females (a) and males (b).

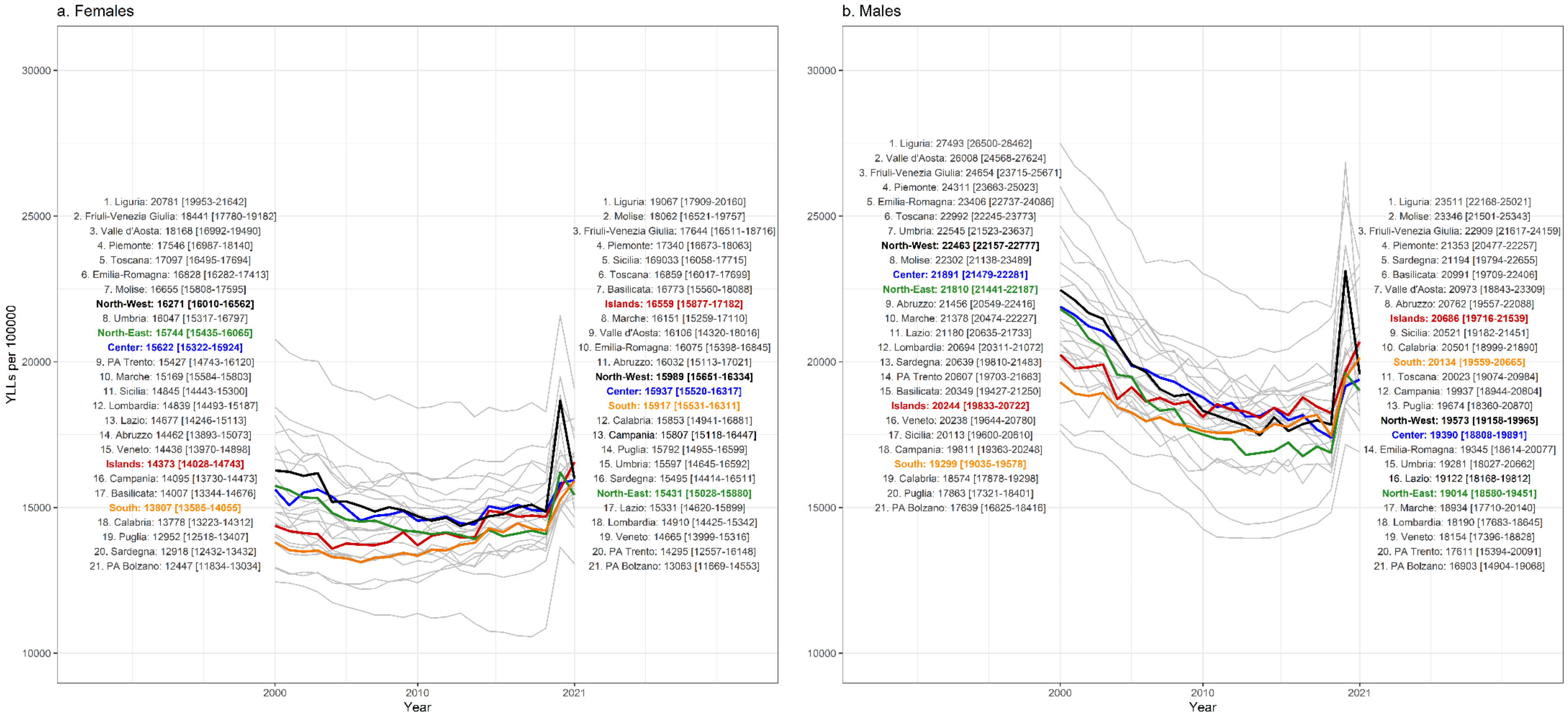

Figure S10. Leading causes of all-age Years of Life Lost (YLLs) in Italy in 2000 and 2019 – Both sexes.

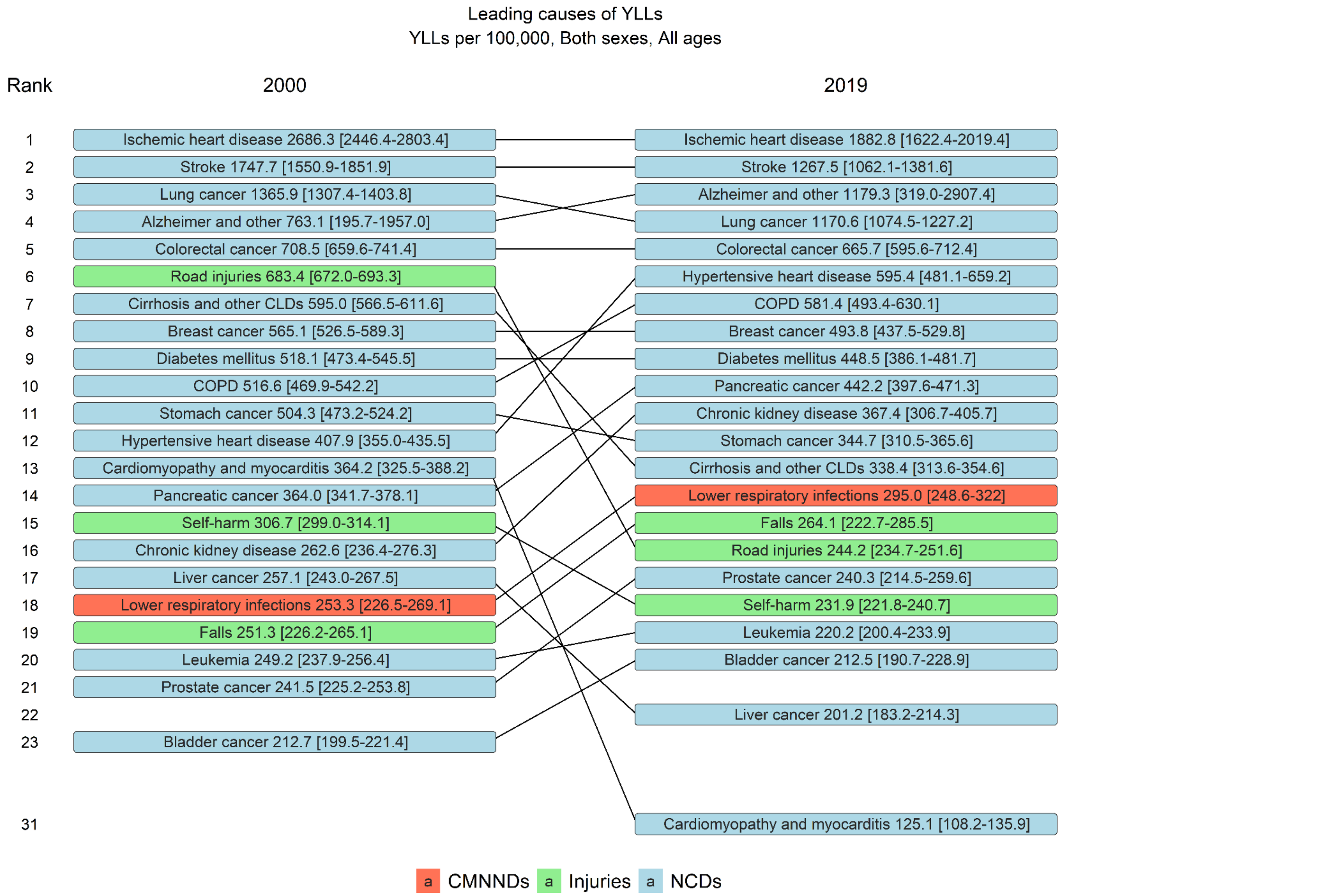

Abbreviations: CLDs: Chronic Liver Diseases; COPD: Chronic Obstructive Pulmonary Disease; CMNNDs: Communicable, Maternal, Neonatal and Nutritional Diseases; NCDs: Non-Communicable Diseases.

Figure S11a. Leading causes of all-age Years of Life Lost (YLLs) in Italy in 2000 and 2019 - Males.

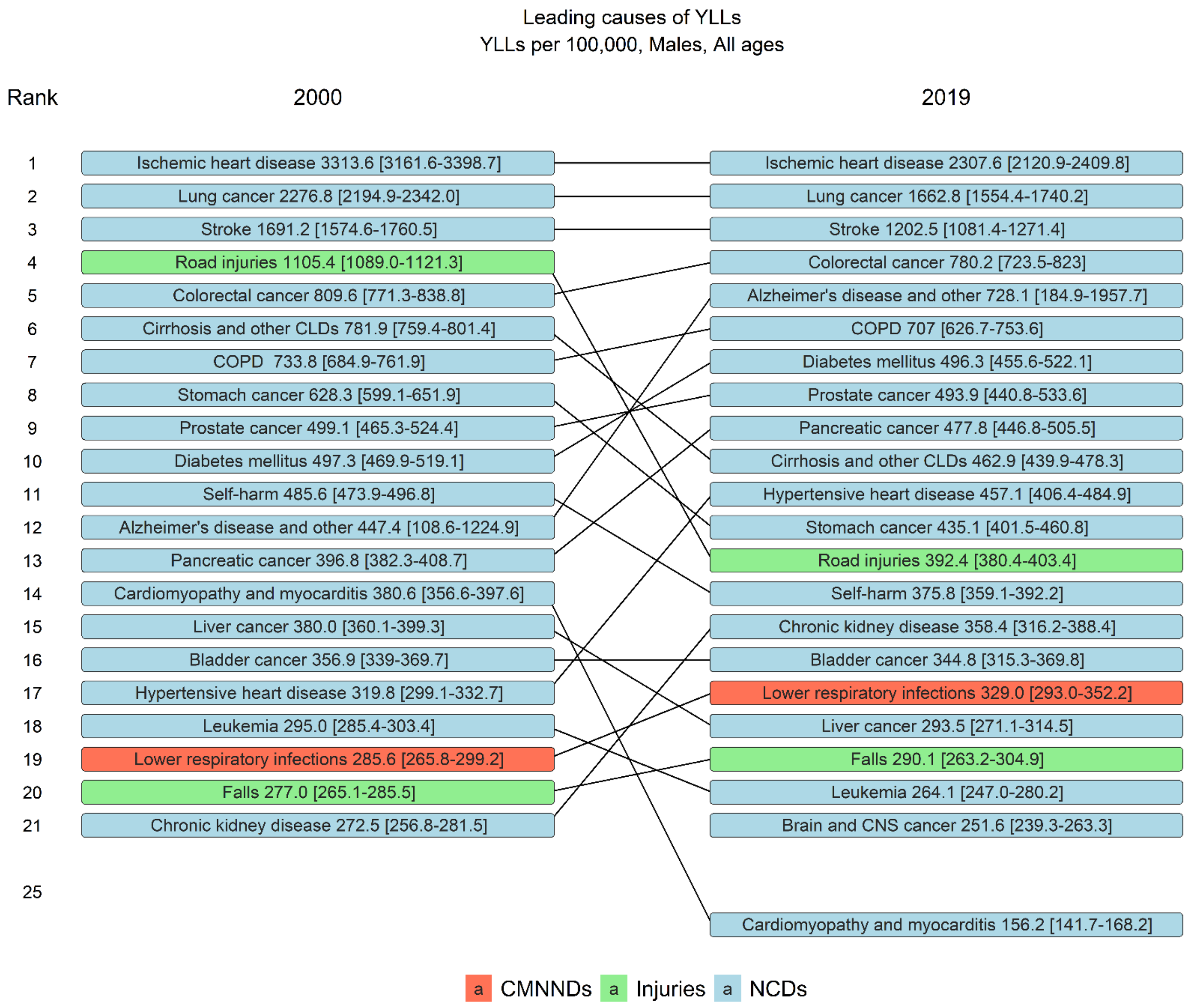

Abbreviations: CLDs: Chronic Liver Diseases; COPD: Chronic Obstructive Pulmonary Disease; CNS: Central Nervous System; CMNNDs: Communicable, Maternal, Neonatal and Nutritional Diseases; NCDs: Non-Communicable Diseases.

Figure S11b. Leading causes of all-age Years of Life Lost (YLLs) in Italy in 2000 and 2019 - Females.

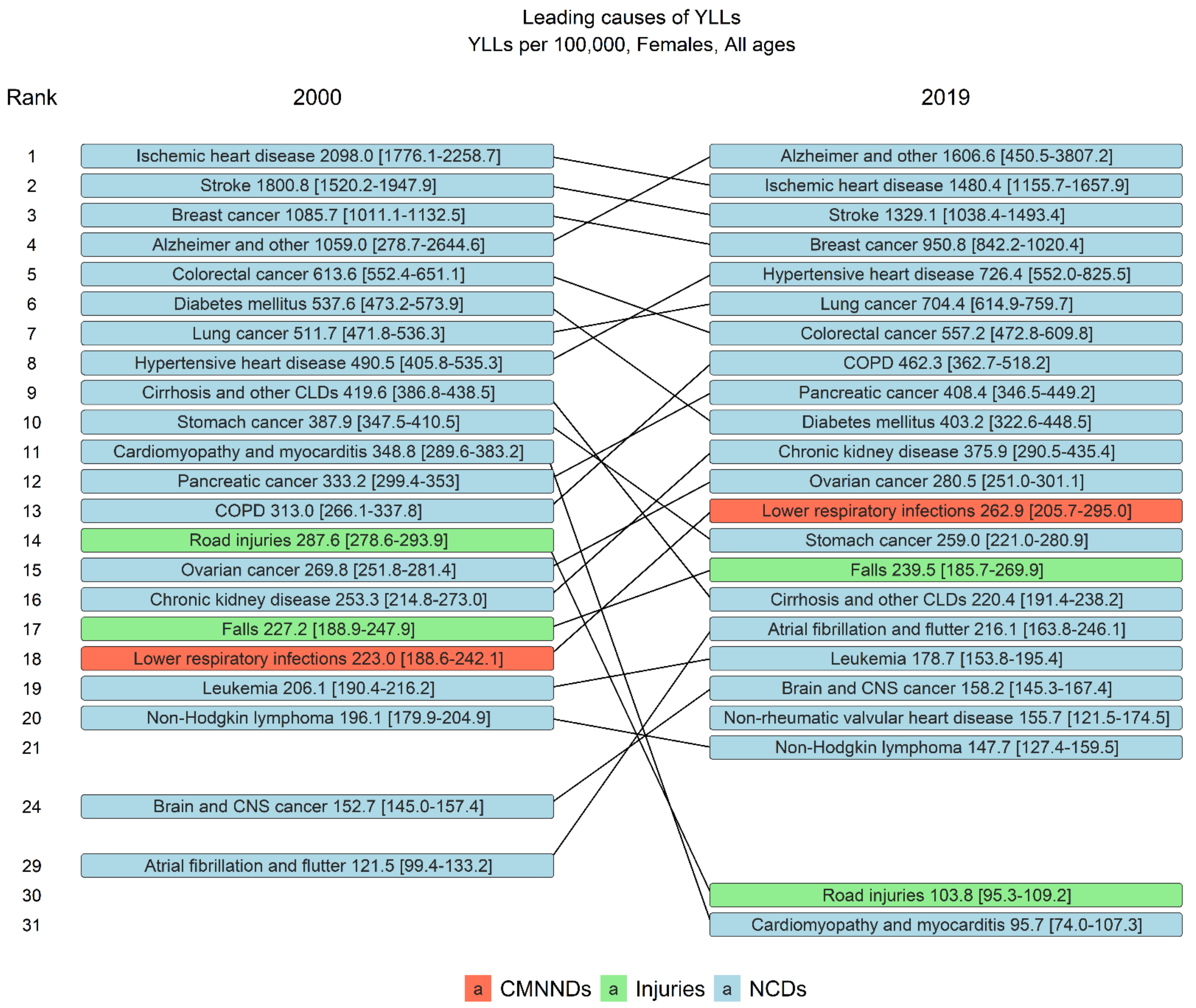

Abbreviations: CLDs: Chronic Liver Diseases; COPD: Chronic Obstructive Pulmonary Disease; CNS: Central Nervous System; CMNNDs: Communicable, Maternal, Neonatal and Nutritional Diseases; NCDs: Non-Communicable Diseases.

Figure S12. Trends of age-standardised Years of Life Lost (YLLs) by region in Italy between 2000 and 2021 for females (a) and males (b).

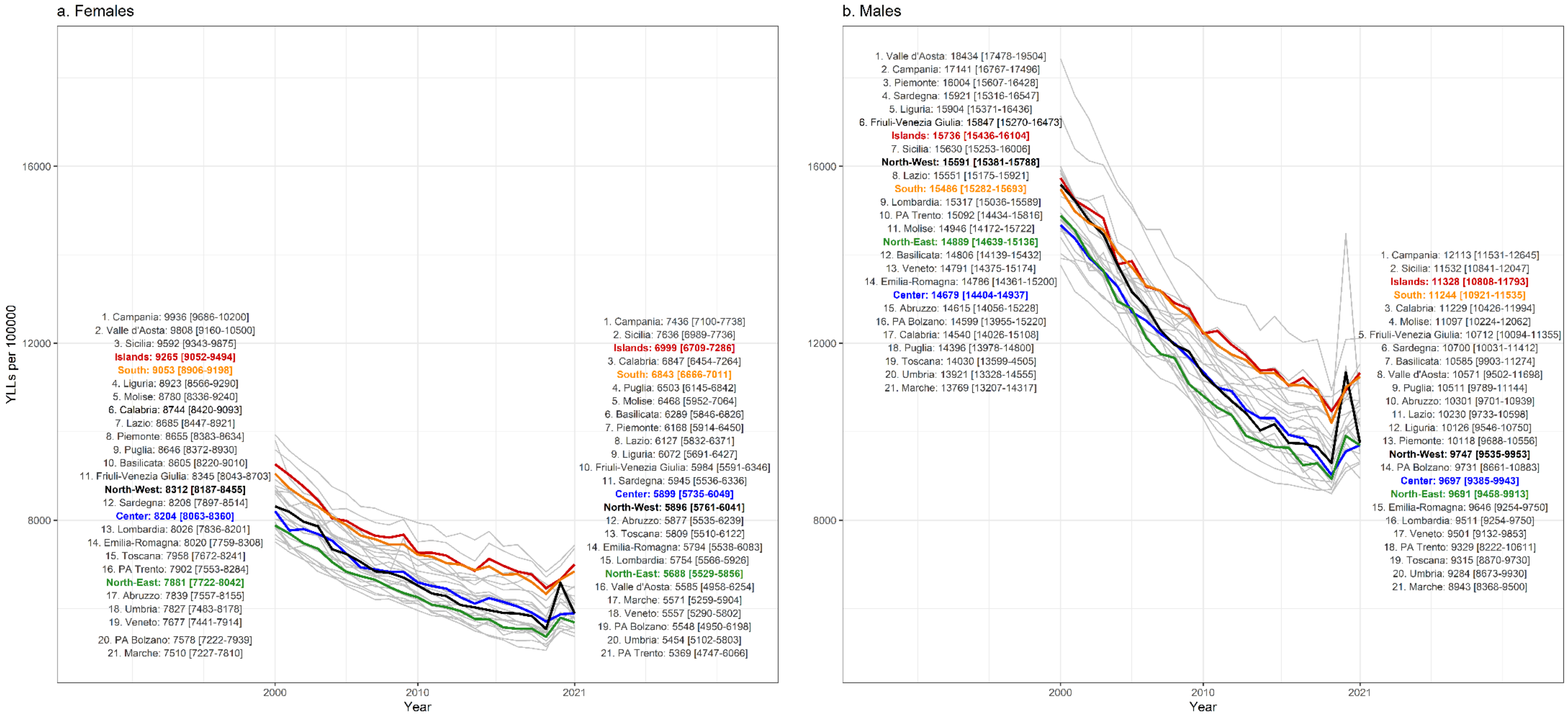

Figure S13. Leading causes of age-standardised Years of Life Lost (YLLs) in Italy in 2000 and 2019 – Both sexes.

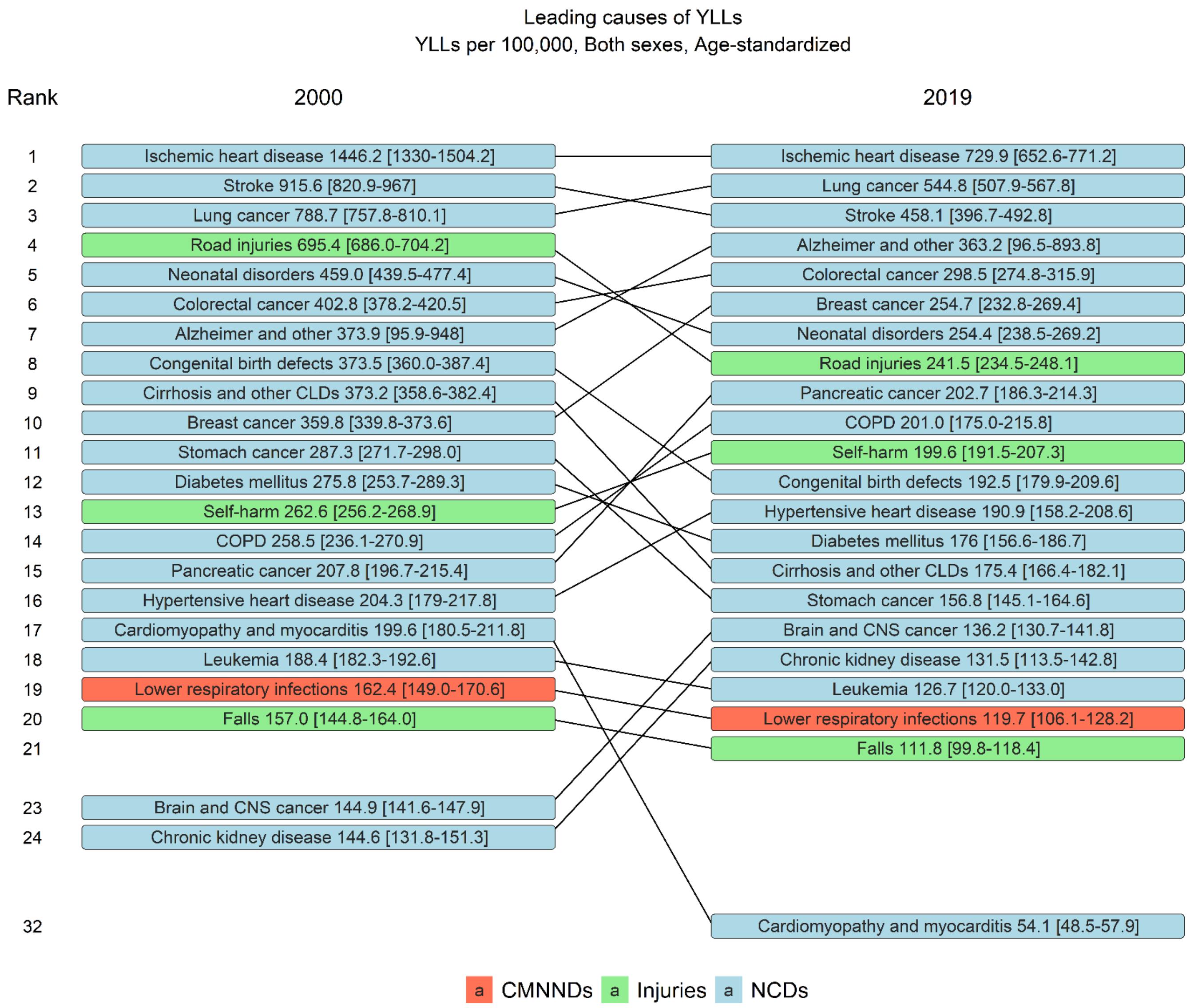

Abbreviations: CLDs: Chronic Liver Diseases; COPD: Chronic Obstructive Pulmonary Disease; CNS: Central Nervous System; CMNNDs: Communicable, Maternal, Neonatal and Nutritional Diseases; NCDs: Non-Communicable Diseases.

Figure S14a. Leading causes of age-standardised Years of Life Lost (YLLs) in Italy in 2000 and 2019 - Males.

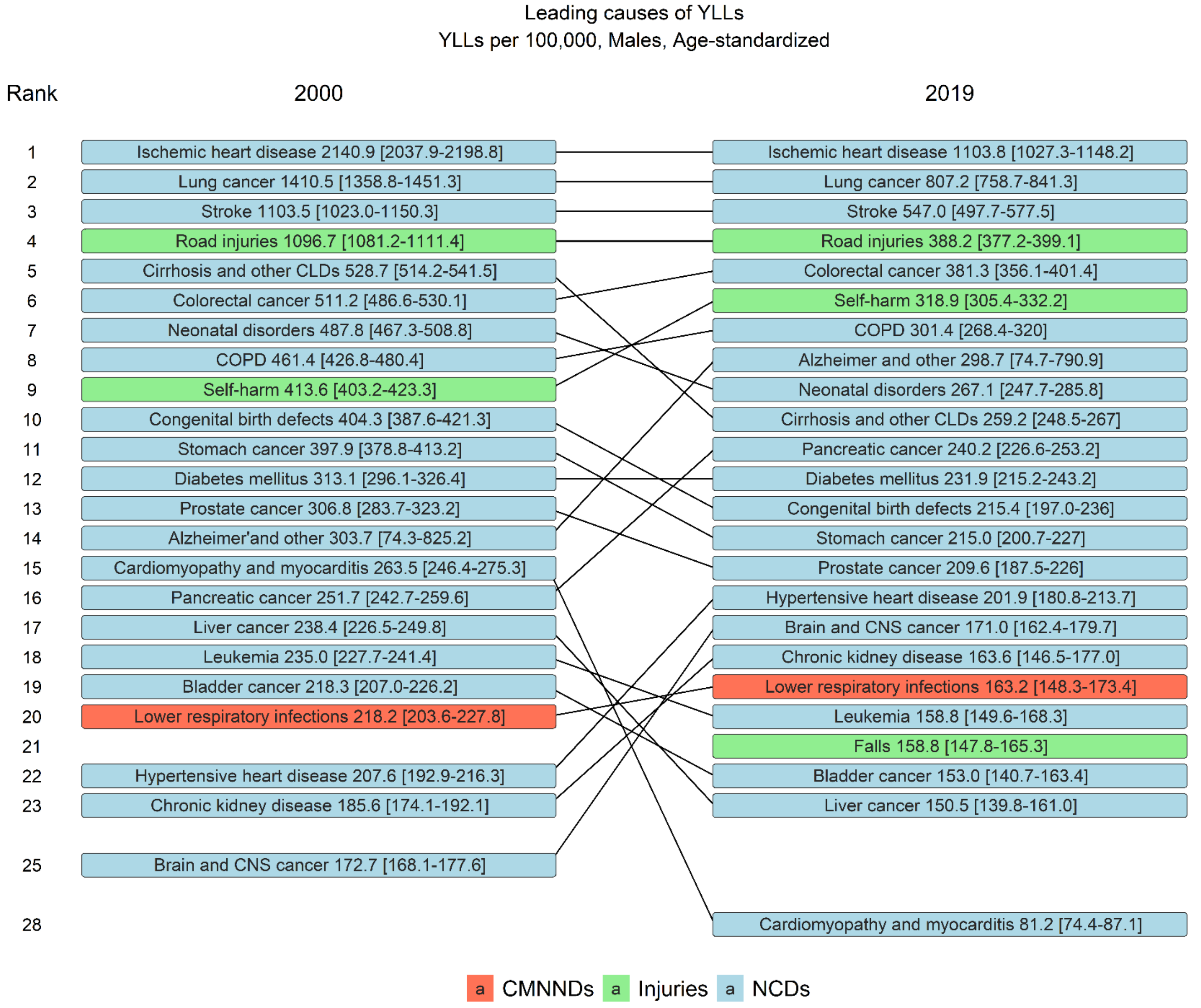

Abbreviations: CLDs: Chronic Liver Diseases; COPD: Chronic Obstructive Pulmonary Disease; CNS: Central Nervous System; CMNNDs: Communicable, Maternal, Neonatal and Nutritional Diseases; NCDs: Non-Communicable Diseases.

Figure S14b. Leading causes of age-standardised Years of Life Lost (YLLs) in Italy in 2000 and 2019 - Females.

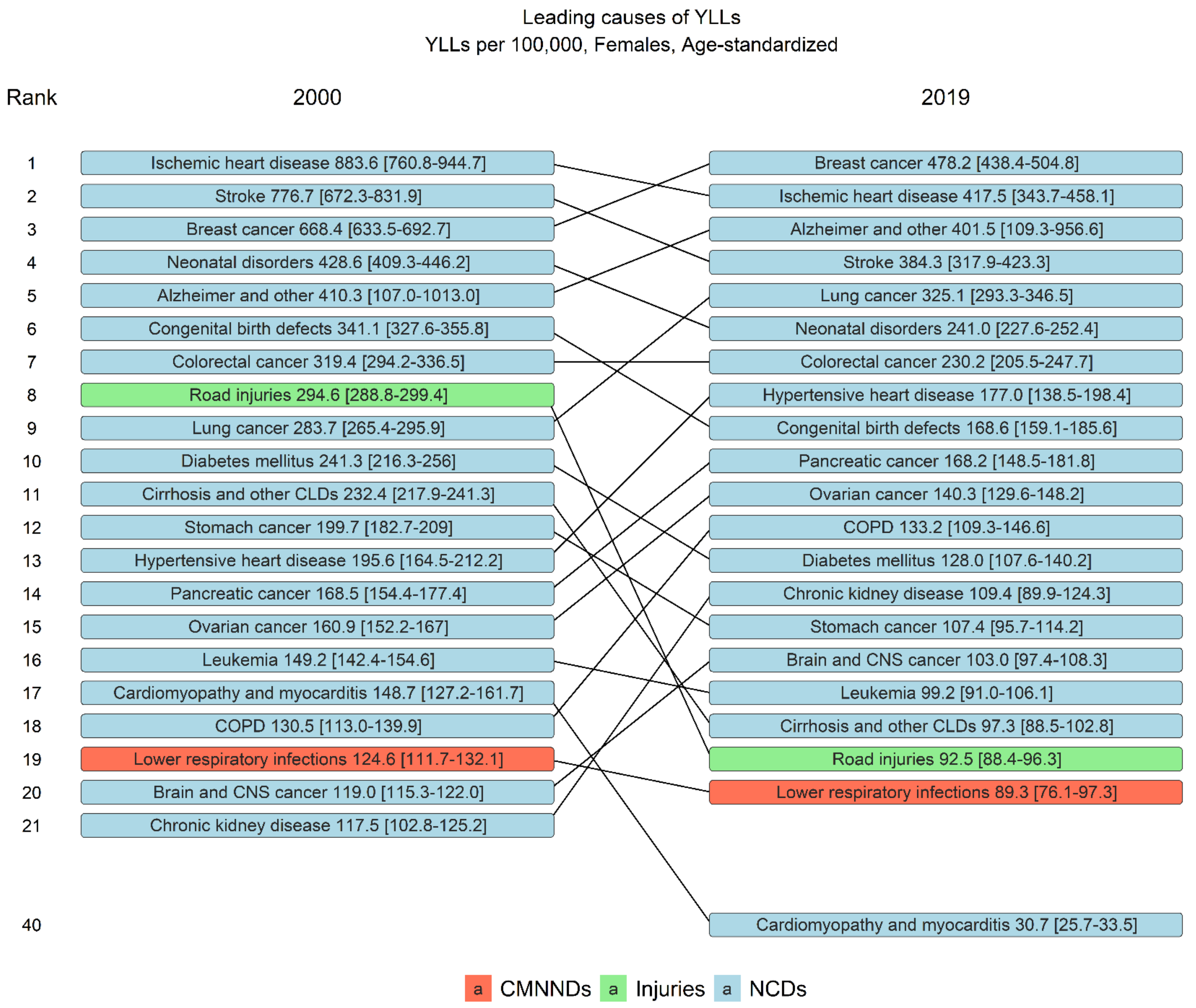

Abbreviations: CLDs: Chronic Liver Diseases; COPD: Chronic Obstructive Pulmonary Disease; CNS: Central Nervous System; CMNNDs: Communicable, Maternal, Neonatal and Nutritional Diseases; NCDs: Non-Communicable Diseases.

Figure S15. Ten leading causes of age-standardised Years of Life Lost (YLLs) in 2019 in Italy and in the five macro-regions. Rate are expressed per 100,000 people.

Italy

Rank 2019	Cause	YLL rate (95% UI)	Change from 2000 to 2019	Change from 2019 to 2021
1	Ischemic heart disease	729.9 (652.6-771.2)	-52.1%	-4.8%
2	Lung cancer	544.8 (507.9-567.8)	-35.0%	-3.9%
3	Stroke	458.1 (396.7-492.8)	-49.1%	-4.6%
4	Alzheimer and other	363.2 (96.5-893.8)	-3.3%	-3.8%
5	Colorectal cancer	298.5 (274.8-315.9)	-31.0%	-4.0%
6	Breast cancer	254.7 (238.5-269.2)	-34.5%	-5.1%
7	Neonatal disorders	254.4 (238.5-269.2)	-70.0%	-9.9%
8	Road injuries	241.5 (234.5-248.1)	-39.8%	-3.4%
9	Pacreatic cancer	202.7 (186.3-214.3)	-23.2%	-10.6%
10	COPD	201.0 (175.0-215.8)	-5.1%	-3.5%

North-West

Rank 2019	Cause	YLL rate (95% UI)	Change from 2000 to 2019	Change from 2019 to 2021
1	Ischemic heart disease	662.5 (589.7-704.1)	-52.1%	-4.8%
2	Lung cancer	557.9 (514.2-593.5)	-35.0%	-3.9%
3	Stroke	441.2 (377.0-478.9)	-49.1%	-4.6%
4	Alzheimer and other	366.7 (99.0-889.3)	-3.3%	-3.8%
5	Colorectal cancer	289.4 (262.3-314.8)	-31.0%	-4.0%
6	Breast cancer	249.7 (224.0-269.0)	-34.5%	-5.1%
7	Road injuries	226.2 (217.3-234.8)	-70.0%	-9.9%
8	Neonatal disorders	221.6 (203.1-242.4)	-39.8%	-3.4%
9	Self-harm	215.8 (204.9-226)	-23.2%	-10.6%
10	Pancreatic cancer	212.3 (194.5-226.5)	-5.1%	-3.5%

North-East

Rank 2019	Cause	YLL rate (95% UI)	Change from 2000 to 2019	Change from 2019 to 2021
1	Ischemic heart disease	622.2 (549-663.6)	-55.0%	-5.6%
2	Lung cancer	500.4 (460.6-530.5)	-38.5%	-7.6%
3	Alzheimer and other	372.4 (100.3-915.5)	-4.2%	-3.3%
4	Stroke	365.7 (312.5-397.7)	-48.2%	-5.1%
5	Colorectal cancer	276.9 (246.6-300.0)	-33.5%	-7.2%
6	Road injuries	264.7 (255.1-273.9)	-69.2%	-7.2%
7	Breast cancer	233.4 (211.8-248.8)	-34.2%	-9.0%
8	Self-harm	229.1 (218.6-238.1)	-26.2%	-10.4%
9	Pancreatic cancer	217.5 (197.5-231.7)	-7.5%	-6.9%
10	Neonatal disorders	212.2 (194.5-227.7)	-36.3%	-4.6%

Center

Rank 2019	Cause	YLL rate (95% UI)	Change from 2000 to 2019	Change from 2019 to 2021
1	Ischemic heart disease	720.7 (641.8-766.8)	-50.1%	-2.8%
2	Lung cancer	558.9 (515.9-591.5)	-28.2%	-4.0%
3	Stroke	439.3 (382-472.2)	-50.4%	-3.5%
4	Alzheimer and other	340.5 (88.6-852.0)	-1.8%	-4.0%
5	Colorectal cancer	294.6 (266.7-321.4)	-29.6%	-4.0%
6	Breast cancer	248.2 (226.9-264.9)	-27.7%	-6.6%
7	Road injuries	246.1 (236.5-255.3)	-65.1%	-4.9%
8	Neonatal disorders	218.8 (203.7-232.8)	-49.8%	-11.0%
9	Pancreatic cancer	194.8 (178.8-207.7)	-3.3%	-3.7%
10	COPD	187.3 (162.5-201.3)	-17.9%	-2.6%

South

Rank 2019	Cause	YLL rate (95% UI)	Change from 2000 to 2019	Change from 2019 to 2021
1	Ischemic heart disease	888.6 (801.4-942.2)	-42.9%	-1.2%
2	Lung cancer	551.0 (517.1-581.2)	-24.7%	-1.4%
3	Stroke	519.8 (458.7-557.5)	-51.7%	-1.7%
4	Alzheimer and other	355.1 (93.1-889.4)	-2.1%	-2.8%
5	Colorectal cancer	314.3 (286.5-339.2)	-13.0%	-1.4%
6	Neonatal disorders	310.2 (288.5-331.7)	-44.9%	-2.0%
7	Hypertensive heart disease	283.5 (237.5-308.3)	-7.4%	-2.3%
8	Breast cancer	270.0 (250.0-286.9)	-21.1%	-3.2%
9	COPD	261.1 (229.0-280.4)	-24.1%	-0.8%
10	Diabetes mellitus	257.4 (230.6-274.6)	-35.4%	-0.7%

Islands

Rank 2019	Cause	YLL rate (95% UI)	Change from 2000 to 2019	Change from 2019 to 2021
1	Ischemic heart disease	802.3 (720.1-851.7)	-45.8%	-0.5%
2	Stroke	587.2 (508.6-637.1)	-51.4%	-1.0%
3	Lung cancer	547.9 (505-586.5)	-20.7%	-1.0%
4	Alzheimer and other	396.8 (104.4-958.9)	-2.2%	-3.8%
5	Neonatal disorders	350.8 (325.5-375.3)	-44.6%	0.2%
6	Colorectal cancer	334.0 (300.6-367.2)	-13.7%	-0.9%
7	Breast cancer	282.6 (258.1-304.3)	-25.0%	-2.5%
8	Road injuries	258.3 (247.0-269.3)	-55.9%	-11.4%
9	Hypertensive heart disease	255.4 (213.1-282.9)	-9.9%	-1.4%
10	Congenital birth defects	249.0 (229.4-275.6)	-48.3%	-2.4%

NCDs **CMNNDs** **Injuries**

Change 2000: YLL rate change (%) from 2000 to 2019; Change 2021: YLL rate change (%) from 2019 to 2021;
Abbreviations: COPD: Chronic Obstructive Pulmonary Disease; NCDs: Non-Communicable Diseases; CMNNDs: Communicable, Maternal, Neonatal, Nutritional Disorders.

Table S3. Ten leading causes of all-age Years of Life Lost (YLLs) in 2019 by region.

Region	Rank (2019)	Cause	YLLs per 100000 [95% UI]	Change 2000 (%)	Change 2021 (%)
Piemonte - North-West	1	Ischemic heart disease	1901.5 [1633.4-2050.2]	-30.2	0.3
	2	Stroke	1635.3 [1361.5-1797.3]	-26.3	0.0
	3	Lung cancer	1314.8 [1186.4-1427]	-20.5	16.5
	4	Alzheimer and other	1289.1 [346-3231.1]	48.4	2.6
	5	Colorectal cancer	764.3 [671.6-857.6]	-9.8	65.5
	6	COPD	681.9 [580.5-744.1]	-13.7	12.7
	7	Hypertensive heart disease	621.1 [497.2-692.3]	-5.2	11.5
	8	Breast cancer	537.7 [465.3-596.3]	-15.1	15.0
	9	Pancreatic cancer	453.4 [399.6-491.2]	-24.1	17.2
	10	Lower respiratory infections	444.4 [371.9-493.8]	-21.8	3.2
Valle d'Aosta - North-West	1	Ischemic heart disease	1913 [1626.4-2108.5]	-33.9	1.6
	2	Stroke	1353.8 [1091.3-1522.7]	-29.1	3.9
	3	Alzheimer and other	1195.3 [318.9-2861.9]	-19.2	16.6
	4	Lung cancer	1158.1 [1013-1313]	36.4	1.6
	5	COPD	692.4 [573.6-771]	-12.3	67.1
	6	Colorectal cancer	642.4 [547.4-740.2]	-17.4	10.4
	7	Breast cancer	490.6 [406.5-573.8]	-36.8	31.8
	8	Hypertensive heart disease	482.5 [385.4-550.8]	-30.4	2.8
	9	Pancreatic cancer	453 [398.8-503.9]	-32.7	9.5
	10	Lower respiratory infections	420.6 [341.9-473.4]	-36.0	8.9

Region	Rank (2019)	Cause	YLLs per 100000 [95% UI]	Change 2000 (%)	Change 2021 (%)
Lombardia - North-West	1	Ischemic heart disease	1673 [1414.8-1817.4]	-35.5	-1.2
	2	Lung cancer	1214.2 [1095.1-1317.4]	-19.4	34.6
	3	Alzheimer and other	1212.9 [340.4-2907.1]	-17.9	0.5
	4	Stroke	1119.8 [911.1-1241.9]	45.7	7.1
	5	Colorectal cancer	615.3 [533.8-695]	-14.0	79.8
	6	COPD	497.7 [405.4-549.8]	-28.3	22.3
	7	Breast cancer	495.7 [424.5-549.5]	-19.5	1.1
	8	Pancreatic cancer	490.5 [429.2-533.2]	-17.6	-0.8
	9	Stomach cancer	397.9 [352.5-436.8]	-27.7	21.5
	10	Hypertensive heart disease	393.7 [310.4-444.3]	-5.0	-1.3
AP Bolzano - North-East	1	Ischemic heart disease	1457 [1242.1-1603.5]	-38.3	52.3
	2	Alzheimer and other	994.4 [270.4-2367]	-16.2	50.5
	3	Stroke	872 [716.9-972.5]	-8.0	14.5
	4	Lung cancer	839 [748.5-930.8]	13.7	9.6
	5	Colorectal cancer	556.7 [480.1-634.1]	-14.1	43.3
	6	COPD	410 [341.6-452.5]	-35.3	33.8
	7	Pancreatic cancer	400.1 [352.3-436.7]	-13.0	6.6
	8	Hypertensive heart disease	374.3 [291.1-428.4]	-16.9	10.0
	9	Breast cancer	361.6 [310-408.1]	-15.7	8.9
	10	Lower respiratory infections	319.8 [268.1-356.7]	-23.3	10.2
AP Trento - North-East	1	Ischemic heart disease	1735.3 [1422.6-1922.3]	-43.8	9.9
	2	Alzheimer and other	1214.2 [319.8-2922.5]	-5.6	45.1
	3	Lung cancer	977.9 [861.4-1078.9]	-22.9	21.0
	4	Stroke	855.3 [685.5-960.2]	12.3	8.6
	5	Colorectal cancer	607.7 [513.7-694.5]	-19.2	43.3
	6	Breast cancer	462.9 [386.9-523.2]	-24.7	27.2
	7	Pancreatic cancer	432.9 [380.8-475.8]	-28.0	-1.4
	8	Hypertensive heart disease	409.5 [312.8-467.7]	-31.5	2.6
	9	COPD	401.9 [332.2-449]	-18.5	2.9
	10	Cirrhosis and other CLDs	324.9 [291.3-353.6]	-28.4	26.7

Region	Rank (2019)	Cause	YLLs per 100000 [95% UI]	Change 2000 (%)	Change 2021 (%)
Veneto - North East	1	Ischemic heart disease	1657.7 [1412.2-1803.9]	-32.1	15.9
	2	Alzheimer and other	1176.1 [314.5-2869.4]	-20.6	39.2
	3	Lung cancer	1047.5 [934.5-1140.5]	-21.6	14.2
	4	Stroke	1038.6 [851.9-1151.1]	27.8	-1.1
	5	Colorectal cancer	626.4 [530.8-706.9]	-19.2	61.9
	6	Hypertensive heart disease	537.1 [424.2-603.4]	-24.2	13.4
	7	Pancreatic cancer	475.5 [415.3-520]	-14.9	12.3
	8	Breast cancer	471.1 [409-524.5]	-11.6	-1.8
	9	COPD	416.8 [344.6-461.7]	-2.8	7.3
	10	Diabetes mellitus	367 [312.2-408]	-13.3	13.6
Friuli-Venezia Giulia - North-East	1	Ischemic heart disease	2040.9 [1731.3-2231.7]	-38.2	61.7
	2	Alzheimer and other	1354.9 [370-3316.6]	-19.9	47.5
	3	Stroke	1302.7 [1066.3-1445.7]	-20.1	1.5
	4	Lung cancer	1208.6 [1082.9-1317.5]	25.5	5.7
	5	Colorectal cancer	757.9 [655.1-853.1]	-18.8	54.3
	6	COPD	591.5 [489.3-656.6]	-24.8	24.2
	7	Breast cancer	571.4 [487.2-638.2]	-21.8	3.7
	8	Pancreatic cancer	565 [500.4-621.5]	-22.4	-2.8
	9	Hypertensive heart disease	549.2 [435.7-623.4]	-13.8	-2.6
	10	Lower respiratory infections	403.7 [333.9-447.7]	-27.0	31.5
Liguria - North-West	1	Ischemic heart disease	2241.4 [1877-2452]	-39.5	-2.0
	2	Stroke	1700.6 [1388.3-1892.7]	-23.9	-0.8
	3	Alzheimer and other	1627.1 [439.8-3889.9]	-14.1	2.4
	4	Lung cancer	1412.8 [1270.6-1548.3]	24.1	15.1
	5	Colorectal cancer	866.5 [753.4-967.2]	-18.8	58.4
	6	Hypertensive heart disease	719.1 [552-818.2]	-9.8	17.2
	7	COPD	710.1 [598.9-784.8]	6.9	-0.2
	8	Breast cancer	589.6 [492.1-666]	-8.9	19.2
	9	Pancreatic cancer	546.8 [477.6-600.5]	-14.4	2.9
	10	Diabetes mellitus	521.9 [442.8-580.9]	-11.2	1.9

Region	Rank (2019)	Cause	YLLs per 100000 [95% UI]	Change 2000 (%)	Change 2021 (%)
Emilia-Romagna - North-East	1	Ischemic heart disease	1728.1 [1440.5-1909.5]	-46.5	25.4
	2	Alzheimer and other	1435.5 [400.8-3426.9]	-15.2	18.2
	3	Lung cancer	1188.1 [1051.2-1305.3]	-25.8	19.2
	4	Stroke	1130.1 [918.2-1266.5]	2.0	0.2
	5	Colorectal cancer	627.4 [524.7-716.5]	-27.8	76.9
	6	Hypertensive heart disease	593.5 [454.3-675.8]	-29.3	1.4
	7	COPD	542.1 [441.3-606.5]	-22.6	8.4
	8	Pancreatic cancer	502.3 [439.8-548.4]	-18.4	6.7
	9	Breast cancer	455.1 [392.3-500.1]	-16.5	5.9
	10	Stomach cancer	373.3 [322.1-414]	-20.1	15.2
Toscana - Centre	1	Ischemic heart disease	1892.3 [1634.9-2040.1]	-38.6	0.0
	2	Stroke	1565.4 [1298.7-1715.9]	-30.0	5.7
	3	Lung cancer	1253.7 [1141.4-1369]	-16.8	24.1
	4	Alzheimer and other	1219.1 [319.8-3073]	40.4	1.1
	5	Colorectal cancer	689.9 [596-779.8]	-19.6	77.8
	6	COPD	616.7 [529.8-675.3]	-15.4	10.2
	7	Breast cancer	526.7 [460.4-579.4]	-18.3	17.4
	8	Pancreatic cancer	460.9 [411-497.4]	-22.0	10.7
	9	Hypertensive heart disease	444.5 [359-496.4]	-22.2	2.3
	10	Diabetes mellitus	431 [370.7-474.7]	-14.7	2.5
Umbria - Centre	1	Ischemic heart disease	2178.3 [1795.2-2394]	-36.6	-2.1
	2	Stroke	1403 [1142.6-1561.9]	-33.1	5.3
	3	Alzheimer and other	1376.7 [364.1-3406]	12.0	-0.8
	4	Lung cancer	1077.2 [957.8-1189]	19.0	24.5
	5	Colorectal cancer	693.7 [590.3-781.7]	-15.3	48.5
	6	COPD	609.5 [502.7-678.6]	-11.8	8.8
	7	Hypertensive heart disease	525.2 [411.8-596.8]	-21.7	14.0
	8	Breast cancer	460.9 [394.9-513.7]	-20.1	10.6
	9	Pancreatic cancer	443 [388.1-486.9]	-17.9	-4.0
	10	Diabetes mellitus	418.3 [346.8-462.3]	-15.5	0.6

Region	Rank (2019)	Cause	YLLs per 100000 [95% UI]	Change 2000 (%)	Change 2021 (%)
Marche - Centre	1	Ischemic heart disease	2050.8 [1706-2274.8]	-26.8	1.8
	2	Alzheimer and other	1345.4 [356.9-3311.7]	-29.5	20.8
	3	Stroke	1244.5 [1018.7-1393.2]	-2.3	9.0
	4	Lung cancer	999.2 [878.2-1106.7]	10.5	27.1
	5	Colorectal cancer	685.2 [590.2-773]	-12.2	44.1
	6	COPD	556.3 [449.6-621.3]	-22.9	22.5
	7	Hypertensive heart disease	497.5 [389.6-565.8]	-29.5	13.9
	8	Pancreatic cancer	458.7 [403.4-502.3]	-11.0	11.3
	9	Breast cancer	439.9 [379.9-495.5]	-14.0	4.1
	10	Stomach cancer	418.9 [363.4-465.5]	-1.7	3.8
Lazio - Centre	1	Ischemic heart disease	1967.9 [1707.5-2144]	-29.6	2.6
	2	Lung cancer	1308.1 [1176.2-1417.2]	-14.3	24.5
	3	Stroke	1094.4 [935.6-1195.9]	-25.9	19.7
	4	Alzheimer and other	1046.5 [283.2-2651.6]	40.6	6.5
	5	Colorectal cancer	665.1 [572.8-760.6]	-5.5	58.0
	6	Hypertensive heart disease	588.5 [484-655.1]	-10.1	13.5
	7	COPD	544.9 [467.2-597.5]	-3.4	10.5
	8	Breast cancer	483.2 [426.1-529.8]	-14.2	15.1
	9	Diabetes mellitus	433 [374.3-474]	-15.0	8.4
	10	Pancreatic cancer	416 [372.8-455.1]	-13.9	6.1
Abruzzo - South	1	Ischemic heart disease	2349.2 [2014-2568.6]	-6.7	1.4
	2	Stroke	1302.8 [1095-1440.7]	-28.4	31.2
	3	Alzheimer and other	1218.8 [325.9-3024.2]	12.9	7.9
	4	Lung cancer	973 [873.2-1066]	21.4	25.7
	5	Hypertensive heart disease	720.6 [572.1-805.6]	2.2	34.5
	6	Colorectal cancer	676.1 [597.2-743.2]	-3.0	7.7
	7	COPD	613.1 [516.2-675.7]	0.6	9.8
	8	Diabetes mellitus	471.1 [403.4-517.3]	-19.4	32.8
	9	Breast cancer	432.8 [374.5-479.8]	-25.0	10.1
	10	Chronic kidney disease	388.2 [322.4-432.8]	-22.5	7.4

Region	Rank (2019)	Cause	YLLs per 100000 [95% UI]	Change 2000 (%)	Change 2021 (%)
Molise - South	1	Ischemic heart disease	2769.1 [2385.8-3046.5]	-13.8	-2.4
	2	Stroke	1579.6 [1336.9-1748]	-26.8	35.0
	3	Alzheimer and other	1316.5 [347.5-3305.9]	41.2	16.8
	4	Lung cancer	1044 [932.9-1148.6]	22.4	24.8
	5	Hypertensive heart disease	865.8 [710-969.4]	18.6	15.9
	6	Colorectal cancer	755.5 [656.2-851.2]	4.1	12.8
	7	COPD	664.5 [562.4-735.1]	-5.6	8.7
	8	Diabetes mellitus	629.5 [544.7-696.2]	-2.0	3.4
	9	Cirrhosis and other CLDs	516.5 [468-565.3]	-10.1	19.1
	10	Chronic kidney disease	468 [392.4-524.8]	-18.3	2.8
Campania - South	1	Ischemic heart disease	2141.1 [1896.1-2299.3]	-18.3	3.7
	2	Stroke	1327.7 [1149.7-1445.1]	-28.3	38.0
	3	Lung cancer	1250.9 [1145.9-1349.5]	0.9	9.5
	4	Alzheimer and other	858.9 [226-2139.9]	-3.0	50.2
	5	COPD	663.2 [576.1-718.3]	-5.2	33.6
	6	Hypertensive heart disease	647.1 [532.9-714.3]	11.6	6.4
	7	Diabetes mellitus	633.5 [563.6-684.6]	17.1	5.1
	8	Colorectal cancer	626.8 [547.7-702.3]	18.7	4.9
	9	Breast cancer	487.1 [436.9-534.4]	-3.1	33.0
	10	Cirrhosis and other CLDs	481.6 [449-508.7]	-1.8	3.1
Puglia - South	1	Ischemic heart disease	1738 [1487.3-1892]	-15.2	26.9
	2	Alzheimer and other	1056.3 [285.4-2613.9]	-21.2	73.0
	3	Lung cancer	989.6 [894.1-1071.7]	-7.2	10.9
	4	Stroke	984.1 [821.6-1082.3]	52.1	4.8
	5	Hypertensive heart disease	779.9 [626.8-875.3]	25.2	31.0
	6	COPD	668.2 [565.8-733.4]	11.7	22.6
	7	Colorectal cancer	610.9 [534.6-677.3]	5.6	14.9
	8	Diabetes mellitus	537.8 [456.9-596.9]	-5.8	17.6
	9	Breast cancer	507.3 [453.1-566.6]	-6.0	11.4
	10	Chronic kidney disease	480.6 [391.5-555.8]	-4.4	5.7

Region	Rank (2019)	Cause	YLLs per 100000 [95% UI]	Change 2000 (%)	Change 2021 (%)
Basilicata - South	1	Ischemic heart disease	2157.9 [1872-2361.7]	-12.7	2.2
	2	Stroke	1414.8 [1193.4-1560.7]	-24.7	1.2
	3	Alzheimer and other	1217.2 [325.8-3037.5]	37.8	-0.1
	4	Hypertensive heart disease	1102.8 [899.7-1232.8]	57.2	2.0
	5	Lung cancer	938.3 [851.2-1028.3]	36.1	19.8
	6	COPD	816.4 [684.5-900.2]	22.2	17.5
	7	Colorectal cancer	686.4 [609.9-759.2]	8.6	20.3
	8	Diabetes mellitus	641.9 [553.4-710.6]	2.4	7.9
	9	Cirrhosis and other CLDs	529.6 [480-570.9]	-14.4	23.5
	10	Chronic kidney disease	488.4 [396.3-549]	-19.4	8.2
Calabria - South	1	Ischemic heart disease	2100.5 [1850.6-2264.5]	-12.4	2.0
	2	Stroke	1423.1 [1208.4-1547.9]	-22.0	11.1
	3	Alzheimer and other	1049.1 [270.7-2614.3]	20.3	37.8
	4	Hypertensive heart disease	983.7 [812.2-1089.8]	47.7	5.8
	5	Lung cancer	955.7 [876.6-1032.2]	53.7	4.7
	6	Colorectal cancer	691.3 [619.2-754.1]	11.5	39.6
	7	Diabetes mellitus	639.3 [558-698.5]	14.9	9.5
	8	COPD	633.8 [547.6-691.5]	16.9	2.7
	9	Breast cancer	450.2 [404.4-492.8]	-16.7	43.5
	10	Chronic kidney disease	438.2 [371.7-484.9]	-15.1	2.6
Sicilia - Islands	1	Ischemic heart disease	1956.6 [1707.5-2108.2]	-21.4	3.7
	2	Stroke	1565.5 [1310.3-1724.5]	-28.1	25.8
	3	Alzheimer and other	1164 [309.8-2844.8]	11.2	39.2
	4	Lung cancer	1094.3 [996.8-1183]	44.1	7.2
	5	Hypertensive heart disease	787 [640.1-882.5]	8.5	42.4
	6	Colorectal cancer	693 [611.3-764.3]	13.1	17.8
	7	COPD	663.8 [568.3-725.8]	9.1	7.2
	8	Diabetes mellitus	646.5 [552.7-707.1]	14.7	6.6
	9	Breast cancer	503.3 [444.2-546.9]	-8.5	32.9
	10	Chronic kidney disease	501.2 [413-559]	-5.5	4.4

Region	Rank (2019)	Cause	YLLs per 100000 [95% UI]	Change 2000 (%)	Change 2021 (%)
Sardegna - Islands	1	Ischemic heart disease	1633.1 [1397.4-1775.1]	-24.6	5.4
	2	Lung cancer	1220.2 [1105.6-1335.5]	-20.1	4.1
	3	Stroke	1200.6 [1001.4-1318.6]	5.2	4.9
	4	Alzheimer and other	1138.9 [307-2732.9]	58.2	4.5
	5	Colorectal cancer	769.5 [672.2-870.3]	14.9	51.2
	6	Breast cancer	568.6 [499.4-636.1]	-9.7	40.4
	7	Pancreatic cancer	521.6 [467.6-571]	-14.6	9.1
	8	COPD	520.6 [438.7-574.6]	-4.2	6.7
	9	Cirrhosis and other CLDs	492.6 [450.7-531.3]	7.3	10.3
	10	Hypertensive heart disease	487.8 [391.7-546.6]	7.9	6.4

Change 2000: YLL rate change (%) from 2000 to 2019; Change 2021: YLL rate change (%) from 2019 to 2021.
Abbreviations: COPD: Chronic Obstructive Pulmonary Disease; CLDs: Chronic Liver Diseases; UI: Uncertainty Interval.

Table S4. Ten leading causes of age-standardised Years of Life Lost (YLLs) in 2019 by region.

Region	Rank (2019)	Cause	YLLs per 100000 [95% UI]	Change 2000 (%)	Change 2021 (%)
Piemonte - North-West	1	Ischemic heart disease	683.8 [608.3-732.8]	-47.4	-3.0
	2	Lung cancer	561 [510.6-605.8]	-44.2	15.1
	3	Stroke	506.5 [434.2-550.3]	-40.7	8.6
	4	Alzheimer and other	349.7 [92.1-866.7]	-58.8	40.2
	5	Colorectal cancer	314.6 [280.8-351]	-26.3	5.4
	6	Breast cancer	254 [226.3-280.4]	-36.3	21.2
	7	Road injuries	244 [231.1-257.6]	-34.7	-0.2
	8	Self-harm	237.7 [223.7-252.5]	-34.3	-5.4
	9	COPD	208.2 [180.3-225.6]	-40.1	5.3
	10	Neonatal disorders	207.8 [189.6-225.4]	-37.1	-0.7
Valle d'Aosta - North-West	1	Ischemic heart disease	725.7 [641.6-795.6]	-52.0	-2.9
	2	Lung cancer	522.8 [458.8-595.2]	-45.3	16.0
	3	Stroke	446.9 [373-497.2]	-48.6	12.3
	4	Alzheimer and other	346.2 [92.3-829]	-58.4	27.0
	5	Self-harm	315.6 [286.4-347.9]	-42.4	4.4
	6	Colorectal cancer	275.6 [237.5-315.6]	-42.8	1.9
	7	Road injuries	254.7 [234-279.1]	-46.5	4.3
	8	Breast cancer	243.2 [206.2-284.8]	-43.7	-4.4
	9	COPD	229.6 [194.6-255.1]	-44.9	-1.2
	10	Pancreatic cancer	201.3 [178.3-223]	-44.8	9.8

Region	Rank (2019)	Cause	YLLs per 100000 [95% UI]	Change 2000 (%)	Change 2021 (%)
Lombardia - North-West	1	Ischemic heart disease	650.6 [572.3-697.4]	-54.0	13.2
	2	Lung cancer	557 [508.8-600.6]	-35.1	10.3
	3	Stroke	404.9 [342.4-443.6]	-49.1	31.4
	4	Alzheimer and other	376.8 [104.1-911.2]	-48.1	1.7
	5	Colorectal cancer	272.4 [240.8-305.4]	-32.8	33.7
	6	Breast cancer	247 [217.6-269.6]	-36.9	5.3
	7	Neonatal disorders	225.1 [203.5-250.2]	-40.5	3.8
	8	Pancreatic cancer	222.2 [200.8-238.9]	-35.0	-0.8
	9	Road injuries	221.6 [210-232.9]	-34.8	-3.8
	10	Self-harm	207.8 [195.8-220.3]	-38.4	-4.0
AP Bolzano - North-East	1	Ischemic heart disease	616.3 [541.5-672.3]	-57.5	85.0
	2	Lung cancer	439 [394.7-485]	-42.5	35.5
	3	Stroke	344.8 [293.3-381.3]	-51.7	14.9
	4	Alzheimer and other	340.7 [91.2-822]	-45.3	0.1
	5	Colorectal cancer	275.8 [240.3-313.3]	-37.8	19.6
	6	Self-harm	268.2 [247.7-289]	-35.8	-4.3
	7	Neonatal disorders	243.8 [209.1-283.1]	-38.6	-1.4
	8	Road injuries	225.7 [209.3-240.8]	-37.3	0.7
	9	Breast cancer	209.2 [184-235.4]	-34.2	-6.7
	10	Pancreatic cancer	203.1 [182.1-221.9]	-32.0	-6.8
AP Trento - North-East	1	Ischemic heart disease	620 [530.3-676]	-61.7	45.9
	2	Lung cancer	467.8 [420.8-513.4]	-38.3	27.9
	3	Alzheimer and other	352.7 [92.9-857.1]	-45.7	19.6
	4	Stroke	295 [249.7-326]	-53.3	14.4
	5	Colorectal cancer	273.1 [234.3-310.1]	-35.9	4.8
	6	Road injuries	237.5 [221.1-255.7]	-37.4	5.4
	7	Breast cancer	236.3 [204.1-265.8]	-36.6	-11.1
	8	Self-harm	221.3 [203.3-241.2]	-39.0	-6.0
	9	Congenital birth defects	204.7 [175.4-238.7]	-30.8	-5.1
	10	Pancreatic cancer	200.2 [178.9-218.8]	-32.3	-9.2

Region	Rank (2019)	Cause	YLLs per 100000 [95% UI]	Change 2000 (%)	Change 2021 (%)
Veneto - North East	1	Ischemic heart disease	625.7 [550.6-670.5]	-51.6	38.8
	2	Lung cancer	476.6 [432.2-516.4]	-43.5	23.4
	3	Stroke	367.4 [315-403.5]	-56.2	20.6
	4	Alzheimer and other	364.3 [96.4-901]	-47.5	-2.5
	5	Colorectal cancer	278.1 [240.6-311.5]	-31.6	24.6
	6	Road injuries	263 [249.9-276.6]	-29.7	-1.6
	7	Breast cancer	236 [211.8-257.9]	-32.4	3.4
	8	Self-harm	221.5 [208-236.1]	-33.7	-2.4
	9	Pancreatic cancer	213.5 [193.3-231.9]	-29.0	-5.3
	10	Neonatal disorders	211.3 [192-230.8]	-28.0	-5.6
Friuli-Venezia Giulia - North-East	1	Ischemic heart disease	675.9 [596.9-733.4]	-53.7	95.6
	2	Lung cancer	508.9 [459.4-552.9]	-38.8	24.6
	3	Stroke	388.8 [330.8-427.2]	-52.8	21.4
	4	Alzheimer and other	348.2 [92.2-854.9]	-52.5	5.7
	5	Colorectal cancer	304 [267.2-343]	-34.5	8.2
	6	Road injuries	272.5 [256.9-289.9]	-35.5	3.5
	7	Breast cancer	263.4 [231.3-293.8]	-35.7	-6.5
	8	Self-harm	253.6 [236-272.6]	-29.8	-5.7
	9	Pancreatic cancer	231.7 [209.2-252.2]	-32.5	-1.4
	10	Neonatal disorders	187.4 [162.8-214]	-41.0	15.3
Liguria - North-West	1	Ischemic heart disease	669.9 [592.6-720.7]	-53.7	-2.1
	2	Lung cancer	563.6 [513.5-614.6]	-35.8	12.6
	3	Stroke	462.2 [398.8-506.5]	-44.7	15.2
	4	Alzheimer and other	360.5 [94.6-879.3]	-43.3	22.3
	5	Colorectal cancer	320.8 [285.5-355.8]	-32.3	8.8
	6	Breast cancer	258.1 [226.4-288]	-45.0	17.5
	7	Neonatal disorders	242.3 [202.5-284.2]	-42.2	1.0
	8	Pancreatic cancer	213.1 [193-233.2]	-42.6	9.7
	9	Congenital birth defects	208 [180.1-239.5]	-42.9	-2.9
	10	Road injuries	202.7 [189.6-216.7]	-41.5	-5.3

Region	Rank (2019)	Cause	YLLs per 100000 [95% UI]	Change 2000 (%)	Change 2021 (%)
Emilia-Romagna - North-East	1	Ischemic heart disease	605.6 [524.3-658]	-57.3	52.5
	2	Lung cancer	534.5 [482.5-581]	-42.8	7.0
	3	Alzheimer and other	392.6 [108.2-955.9]	-51.2	25.7
	4	Stroke	368.3 [313.4-405.8]	-48.6	3.0
	5	Road injuries	272 [257.1-287]	-34.4	28.6
	6	Colorectal cancer	268.2 [233.2-301.7]	-34.9	-3.9
	7	Self-harm	227.1 [211.6-244.3]	-41.7	9.4
	8	Breast cancer	224.2 [198.9-244.3]	-35.3	-8.1
	9	Pancreatic cancer	221.3 [199.8-238]	-34.3	-7.0
	10	Neonatal disorders	216.5 [195.8-236.4]	-34.1	-5.1
Toscana - Centre	1	Ischemic heart disease	627.1 [561.6-669.9]	-52.7	10.7
	2	Lung cancer	538.7 [495.8-585.4]	-41.7	12.0
	3	Stroke	474.9 [408.2-515.4]	-37.1	8.1
	4	Alzheimer and other	321.7 [82.8-809.2]	-52.4	41.7
	5	Colorectal cancer	279.6 [246.7-314.4]	-32.3	11.7
	6	Breast cancer	253.8 [228.3-277.5]	-33.0	5.0
	7	Road injuries	230.3 [218.4-242.2]	-34.0	3.2
	8	Self-harm	194.9 [182.7-209.2]	-42.2	11.5
	9	Pancreatic cancer	193.6 [177-207.6]	-40.9	-4.2
	10	COPD	182 [161.1-197.1]	-40.2	0.6
Umbria - Centre	1	Ischemic heart disease	715 [619.2-775.5]	-53.3	-5.7
	2	Lung cancer	476.3 [429.2-524.8]	-47.1	30.6
	3	Stroke	427.2 [360.2-469.6]	-40.0	3.5
	4	Alzheimer and other	354.8 [93.8-874.2]	-42.0	13.3
	5	Colorectal cancer	284.6 [250.6-317.3]	-30.8	17.5
	6	Road injuries	232.1 [216.8-247.8]	-41.5	13.7
	7	Breast cancer	226.3 [198.5-249.9]	-36.6	12.3
	8	Self-harm	219.7 [203.9-236.5]	-38.1	-2.7
	9	Neonatal disorders	191.9 [160.5-226.6]	-42.5	6.4
	10	Pancreatic cancer	190.9 [171.1-208.1]	-38.8	-4.0

Region	Rank (2019)	Cause	YLLs per 100000 [95% UI]	Change 2000 (%)	Change 2021 (%)
Marche - Centre	1	Ischemic heart disease	684.2 [593.9-749.7]	-46.9	-2.4
	2	Lung cancer	442.1 [395.6-486.6]	-47.2	50.2
	3	Stroke	383.9 [325.8-424.9]	-49.3	9.9
	4	Alzheimer and other	357.3 [94.1-890.2]	-46.1	5.2
	5	Colorectal cancer	291.9 [259.6-324.1]	-26.6	19.4
	6	Road injuries	234 [218.4-249.6]	-37.6	19.4
	7	Breast cancer	220.2 [194.2-244.4]	-39.2	-3.8
	8	Self-harm	205.5 [190.9-221.6]	-42.2	1.6
	9	Pancreatic cancer	200.3 [180.1-218.4]	-43.6	-3.7
	10	Congenital birth defects	179.1 [155.2-209.5]	-42.7	1.7
Lazio - Centre	1	Ischemic heart disease	799.5 [713.4-863.2]	-49.7	-2.0
	2	Lung cancer	620.4 [565.9-668.9]	-28.0	24.0
	3	Stroke	427.3 [375.4-462.2]	-50.0	40.6
	4	Alzheimer and other	347.5 [92.3-871.1]	-51.0	19.4
	5	Colorectal cancer	307.5 [269-350.5]	-37.8	7.3
	6	Road injuries	261.1 [247.3-273.7]	-39.6	14.1
	7	Neonatal disorders	257 [239.3-275.7]	-34.3	-3.9
	8	Breast cancer	255.4 [230.7-276.9]	-29.6	-6.6
	9	Hypertensive heart disease	205.6 [173.5-226.2]	-42.4	7.5
	10	COPD	199.3 [174.3-217.1]	-38.2	-0.8
Abruzzo - South	1	Ischemic heart disease	846.3 [750.9-914.4]	-33.8	-3.2
	2	Stroke	447.5 [388-491.8]	-49.1	59.7
	3	Lung cancer	445.9 [405-485.7]	-38.0	-3.2
	4	Alzheimer and other	347.5 [91.2-867.7]	-42.2	23.0
	5	Colorectal cancer	296.1 [265.8-324.7]	-38.6	13.5
	6	Neonatal disorders	274.5 [230.3-317.6]	-33.6	3.1
	7	Road injuries	272.6 [258.5-286.8]	-29.4	-6.9
	8	Congenital birth defects	224.7 [193.3-261.2]	-39.2	4.0
	9	Breast cancer	222.3 [199.1-244.9]	-37.7	-7.4
	10	Hypertensive heart disease	213.2 [174.6-235.4]	-26.5	-3.5

Region	Rank (2019)	Cause	YLLs per 100000 [95% UI]	Change 2000 (%)	Change 2021 (%)
Molise - South	1	Ischemic heart disease	963.3 [860.8-1046.5]	-39.7	-7.1
	2	Stroke	532 [466.9-583.3]	-47.6	66.1
	3	Lung cancer	479.5 [429.5-526.4]	-34.0	2.9
	4	Alzheimer and other	350.7 [90.8-889.1]	-32.8	26.1
	5	Colorectal cancer	325.8 [287.1-364.2]	-27.6	1.9
	6	Road injuries	279.9 [259-302.1]	-34.7	6.8
	7	Cirrhosis and other CLDs	271.4 [248.6-296.7]	-35.7	-10.0
	8	Hypertensive heart disease	246.5 [209.6-272.6]	-37.8	-1.8
	9	Diabetes mellitus	237.6 [214.2-260]	-33.2	-3.0
	10	Breast cancer	234.9 [206.1-265.3]	-24.8	-5.2
Campania - South	1	Ischemic heart disease	1043.6 [941.3-1114.6]	-44.6	-0.7
	2	Lung cancer	676.3 [622.5-727.8]	-49.1	44.7
	3	Stroke	628 [556.2-679.8]	-29.7	6.8
	4	Alzheimer and other	357.6 [92.9-891.9]	-45.7	73.1
	5	Colorectal cancer	333.1 [291.3-371.8]	-41.3	4.9
	6	Neonatal disorders	319.7 [297.3-341.2]	-39.1	3.7
	7	Diabetes mellitus	306.7 [277.7-329]	-36.9	0.3
	8	COPD	301.6 [266-325.4]	-24.2	1.3
	9	Breast cancer	288.2 [260.7-315.1]	-27.1	4.0
	10	Hypertensive heart disease	283.3 [238.2-310.6]	-25.6	0.2
Puglia - South	1	Ischemic heart disease	711.4 [624.6-765.6]	-45.6	46.5
	2	Lung cancer	475.3 [436.1-513.2]	-43.9	49.3
	3	Stroke	392.7 [338-427.9]	-43.8	19.9
	4	Alzheimer and other	359.4 [96.2-880.5]	-43.0	8.6
	5	Colorectal cancer	286.5 [254.3-317.3]	-48.1	23.3
	6	Breast cancer	276.6 [250.7-304.8]	-39.7	2.7
	7	Hypertensive heart disease	275.8 [226.2-306.1]	-35.4	-1.1
	8	COPD	247.3 [212.9-269.7]	-32.9	7.3
	9	Neonatal disorders	244.9 [224.8-265.5]	-32.1	1.7
	10	Road injuries	236.2 [222.2-249.6]	-32.0	4.6

Region	Rank (2019)	Cause	YLLs per 100000 [95% UI]	Change 2000 (%)	Change 2021 (%)
Basilicata - South	1	Ischemic heart disease	819.3 [736-887.7]	-42.2	-1.7
	2	Stroke	502.4 [441.4-548.5]	-52.1	1.8
	3	Lung cancer	443 [405.1-484.6]	-35.6	9.1
	4	Alzheimer and other	354.4 [91.3-897]	-34.2	23.8
	5	Hypertensive heart disease	340.4 [287-376.8]	-34.9	-1.3
	6	Neonatal disorders	317.1 [268.7-368.4]	-30.9	3.6
	7	Colorectal cancer	309.9 [279.4-340.9]	-28.6	-2.2
	8	Road injuries	285.8 [268.1-305.9]	-21.1	2.4
	9	Cirrhosis and other CLDs	269.3 [247.4-291.5]	-23.4	-3.1
	10	COPD	268.5 [231.9-293.8]	-22.0	-3.1
Calabria - South	1	Ischemic heart disease	882.2 [799.9-944.4]	-39.7	-1.9
	2	Stroke	561.3 [495.2-609.1]	-48.2	35.7
	3	Lung cancer	476.9 [438.8-515]	-26.2	15.1
	4	Neonatal disorders	441.4 [402.9-480.5]	-22.0	5.4
	5	Alzheimer and other	348.3 [88.9-871]	-33.8	28.3
	6	Hypertensive heart disease	340.2 [287.8-373.2]	-25.9	-2.8
	7	Colorectal cancer	331.3 [301.7-358.7]	-25.8	-0.2
	8	Diabetes mellitus	270.3 [241.4-294.1]	-27.1	20.0
	9	Congenital birth defects	266.1 [242.7-294.8]	-24.8	0.0
	10	Road injuries	263.2 [248.3-277.8]	-24.1	-0.8
Sicilia - Islands	1	Ischemic heart disease	860.7 [772.7-917.8]	-43.7	10.9
	2	Stroke	642.1 [555.1-698.3]	-50.8	33.4
	3	Lung cancer	549 [503.7-590.1]	-21.1	15.8
	4	Alzheimer and other	413.3 [108.8-1006.8]	-39.0	31.5
	5	Neonatal disorders	387.4 [360.2-414.3]	-29.7	2.8
	6	Colorectal cancer	334.3 [299.4-368.2]	-34.2	17.2
	7	Hypertensive heart disease	290.7 [242.3-323.2]	-35.8	14.0
	8	Breast cancer	282 [255.7-304.7]	-33.1	1.6
	9	Diabetes mellitus	277.1 [243-300.7]	-33.5	0.1
	10	COPD	262.1 [229.8-284.6]	-32.2	5.3

Region	Rank (2019)	Cause	YLLs per 100000 [95% UI]	Change 2000 (%)	Change 2021 (%)
Sardegna - Islands	1	Ischemic heart disease	642.4 [569.8-694.4]	-51.9	-0.2
	2	Lung cancer	545 [497.7-592]	-40.5	-1.1
	3	Stroke	436.4 [377.2-476.1]	-40.6	18.2
	4	Alzheimer and other	351.8 [93.8-839.9]	-48.8	23.4
	5	Colorectal cancer	333.1 [293.2-374.5]	-27.8	1.1
	6	Self-harm	295.8 [276.6-317.2]	-24.3	11.5
	7	Road injuries	290.1 [274.5-306.2]	-25.4	-5.4
	8	Breast cancer	284 [254.2-316.2]	-26.9	-4.6
	9	Cirrhosis and other CLDs	248.1 [228.4-266.9]	-34.4	6.5
	10	Pancreatic cancer	231 [210.4-251]	-37.9	5.3

Change 2000: YLL rate change (%) from 2000 to 2019; Change 2021: YLL rate change (%) from 2019 to 2021.
Abbreviations: COPD: Chronic Obstructive Pulmonary Disease; CLDs: Chronic Liver Diseases; UI: Uncertainty Interval.

Figure S16. Trends of all-age Disability-Adjusted Life-Years (DALYs) by region in Italy between 2000 and 2021 for females (a) and males (b).

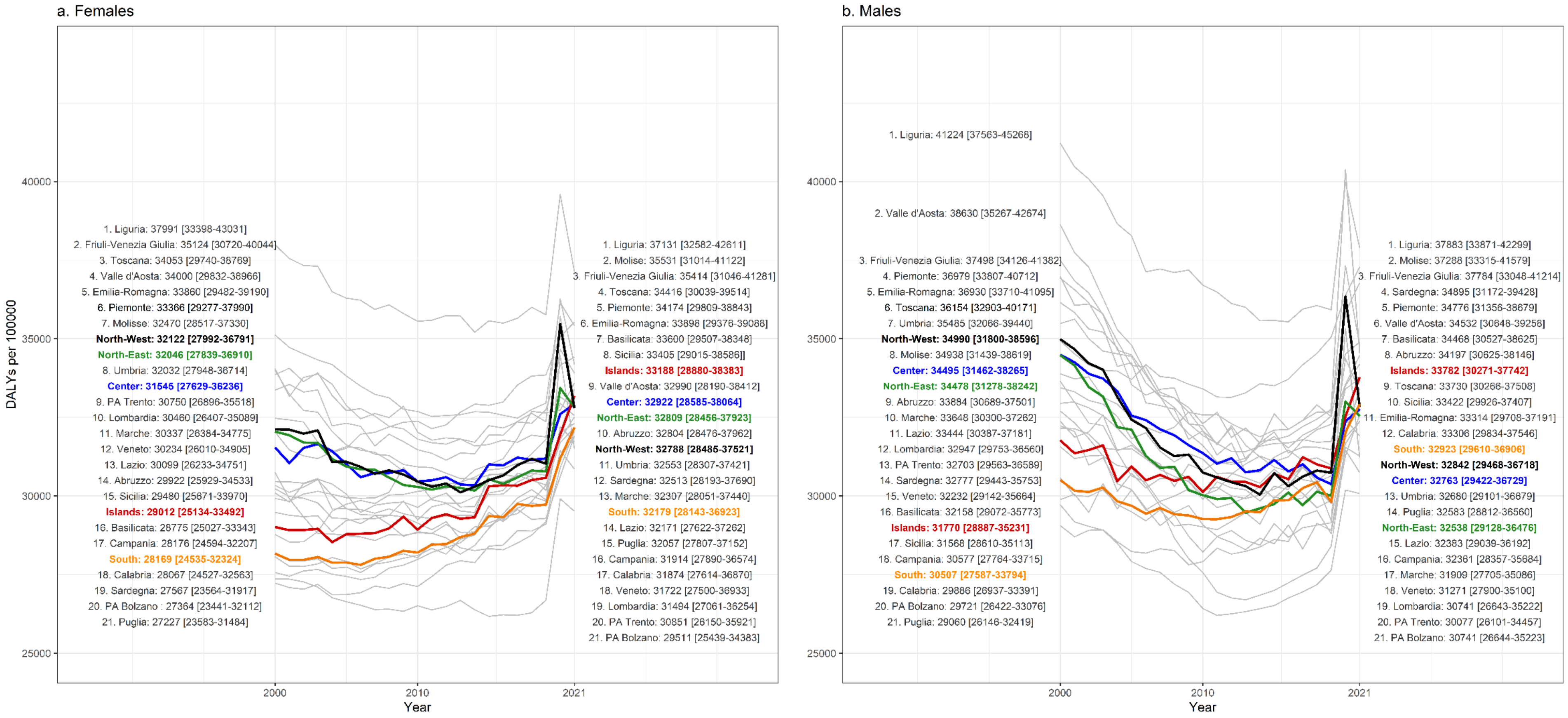

Figure S17. Ten leading causes of all-age Disability-Adjusted Life-Years (DALYs) in 2019 in Italy and the five macro-regions. Rate are expressed per 100,000 people.

Italy

Rank 2019	Cause	DALY rate (95% UI)	Change from 2000 to 2019	Change from 2019 to 2021
1	Ischemic heart disease	1975.7 (1717.0-2118.7)	-29.0%	1.1%
2	Alzheimer and other	1665.4 (789.0-3382.9)	58.9%	1.2%
3	Low back pain	1556.5 (1098.5-2080.2)	6.6%	2.3%
4	Stroke	1442.7 (1235.2-1574.2))	-24.8%	0.9%
5	Falls	1190.3 (911.2-1525.1)	1.1%	1.2%
6	Lung cancer	1186.7 (1089.7-1245.0)	-14.1%	-0.4%
7	Diabetes mellitus	1070.5 (883.6-1320.4)	13.74%	5.2%
8	Headache disorders	858.0 (173.7-1808.2)	-4.6%	-4.8%
9	Depressive disorders	795.3 (546.9-1092.3)	-7.1%	17.3%
10	COPD	792.1 (695.0-864.4)	15.8%	1.6%

North-West

Rank 2019	Cause	DALY rate (95% UI)	Change from 2000 to 2019	Change from 2019 to 2021
1	Ischemic heart disease	1888.8 (1626.3-2043.1)	-33.8%	-0.8%
2	Alzheimer and other	1798.8 (844.9-3613.8)	57.0%	0.6%
3	Low back pain	1952.4 (1128.6-2114.0)	4.3%	1.9%
4	Stroke	1512.4 (1276.7-1669.5)	-23.0%	-0.6%
5	Lung cancer	1278.2 (1157.2-1371.3)	-20.5%	-0.9%
6	Falls	1264.0 (957.5-1626.6)	-4.1%	0.6%
7	Headache disorders	864.2 (175.4-1829.8)	-6.0%	-4.1%
8	Diabetes mellitus	807.0 (662.1-997.2)	2.3%	3.4%
9	COPD	798.2 (695.2-972.2)	17.5%	0.8%
10	Age-related hearing loss	734.7 (523.8-1006.4)	24.9%	1.9%

North-East

Rank 2019	Cause	DALY rate (95% UI)	Change from 2000 to 2019	Change from 2019 to 2021
1	Ischemic heart disease	1819.9 (1557.0-1978.9)	-38.7%	-1.2%
2	Alzheimer and other	1817.5 (875.2-3690.9)	45.4%	0.4%
3	Low back pain	1584.9 (1118.6-2110.1)	3.5%	2.1%
4	Stroke	1266.7 (1062.8-1392.0)	-24.6%	-0.8%
5	Falls	1248.7 (951.3-1607.9)	-1.6%	0.6%
6	Lung cancer	1121.5 (1010.1-1196.5)	-26.4%	-4.0%
7	Diabetes mellitus	982.8 (793.8-1221.2)	22.3%	5.2%
8	Depressive disorders	964.5 (657.2-1324.4)	-4.7%	16.5%
9	Headache disorders	866.8 (172.6-1837.2)	-5.1%	-4.6%
10	Age-related hearing loss	717.8 (511.2-979.0)	19.8%	2.0%

Center

Rank 2019	Cause	DALY rate (95% UI)	Change from 2000 to 2019	Change from 2019 to 2021
1	Ischemic heart disease	2065.3 (1798.9-2227.9)	-32.1%	1.3%
2	Alzheimer and other	1654.2 (784.2-3379.7)	54.5%	1.1%
3	Low back pain	1567.8 (1109.5-2111.1)	4.5%	2.7%
4	Stroke	1470.8 (1261.3-1605.4)	-27.7%	0.7%
5	Lung cancer	1250.2 (1140.8-1333.3)	-14.0%	-0.9%
6	Falls	1235.8 (947.8-1580.9)	-0.9%	1.3%
7	Diabetes mellitus	1010.0 (827.3-1241.5)	8.7%	4.7%
8	Headache disorders	854.2 (174.4-1796.4)	-4.7%	5.2%
9	Depressive disorders	821.9 (563.4-1133.9)	-10.5%	16.9%
10	COPD	787.2 (690.6-856.9)	16.5%	1.1%

South

Rank 2019	Cause	DALY rate (95% UI)	Change from 2000 to 2019	Change from 2019 to 2021
1	Ischemic heart disease	2138.3 (1877.1-2289.3)	-14.5%	3.2%
2	Low back pain	1501.3 (1055.6-2019.5)	11.5%	2.5%
3	Alzheimer and other	1410.6 (664.2-2923.9)	73.9%	2.7%
4	Stroke	1398.2 (1222.0-1515.1)	24.3%	2.8%
5	Diabetes mellitus	1242.4 (1045.7-1506.9)	18.3%	6.2%
6	Falls	1127.8 (863.2-1444.9)	12.7%	1.8%
7	Lung cancer	1104.7 (1026.3-1169.0)	-0.6%	2.4%
8	Headache disorders	865.4 (171.1-1826.3)	-3.2%	-5.2%
9	COPD	855.7 (753.5-927.6)	19.8%	3.1%
10	Hypertensive heart disease	808.7 (667.3-883.6)	53.0%	2.5%

Islands

Rank 2019	Cause	DALY rate (95% UI)	Change from 2000 to 2019	Change from 2019 to 2021
1	Ischemic heart disease	1959.9 (1717.3-2106.5)	-21.3%	4.0%
2	Stroke	1628.9 (1402.3-1774.4)	24.5%	3.8%
3	Alzheimer and other	1628.4 (771.9-3306.2)	68.4%	1.9%
4	Diabetes mellitus	1609.9 (1299.2-1981.8)	23.6%	6.5%
5	Low back pain	1515.2 (1067.8-2034.9)	10.9%	2.5%
6	Lung cancer	1139.8 (1040.7-1226.8)	5.4%	2.6%
7	Falls	985.7 (753.5-1210.6)	-1.3%	2.1%
8	COPD	819.2 (715.4-894.9)	21.4%	3.7%
9	Headache disorders	819.0 (174.1-1715.1)	-3.7%	-5.1%
10	Colorectal cancer	761.9 (674.2-841.1)	18.8%	2.9%

NCDs CMNNDs Injuries

Change 2000: DALY rate change (%) from 2000 to 2019; Change 2021: DALY rate change (%) from 2019 to 2021.

Abbreviations: COPD: Chronic Obstructive Pulmonary Disease; NCDs: Non-Communicable Diseases; CMNNDs: Communicable, Maternal Neonatal Nutritional Disorders.

Figure S18. Leading causes of all-age Disability-Adjusted Life-Years (DALYs) in Italy in 2000 and 2019 – Both sexes.

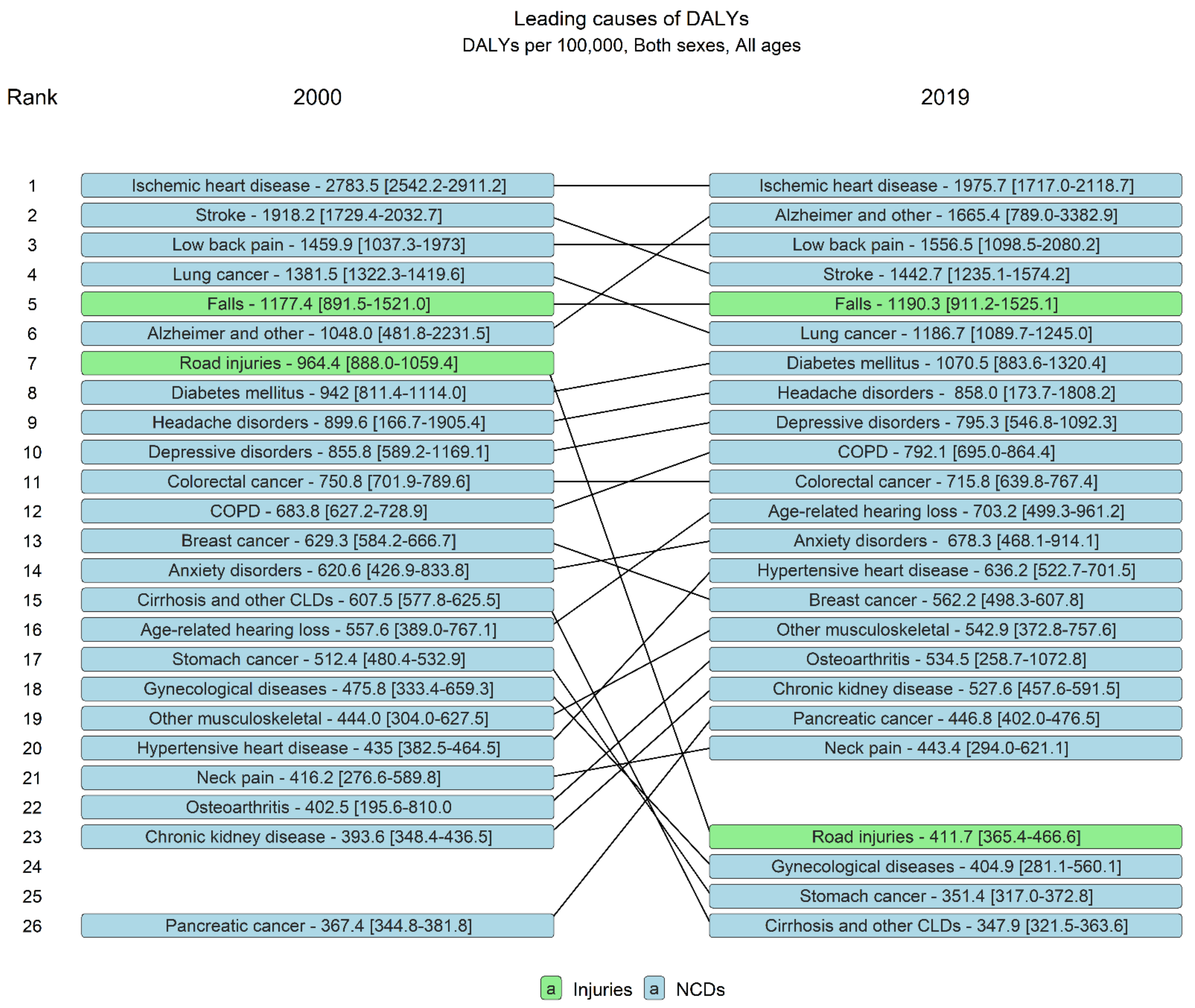

Abbreviations: COPD: Chronic Obstructive Pulmonary Disease; CLDs: Chronic Liver Diseases; NCDs: Non-Communicable Diseases.

Figure S19a. Leading causes of all-age Disability-Adjusted Life-Years in Italy in 2000 and 2019 - Males.

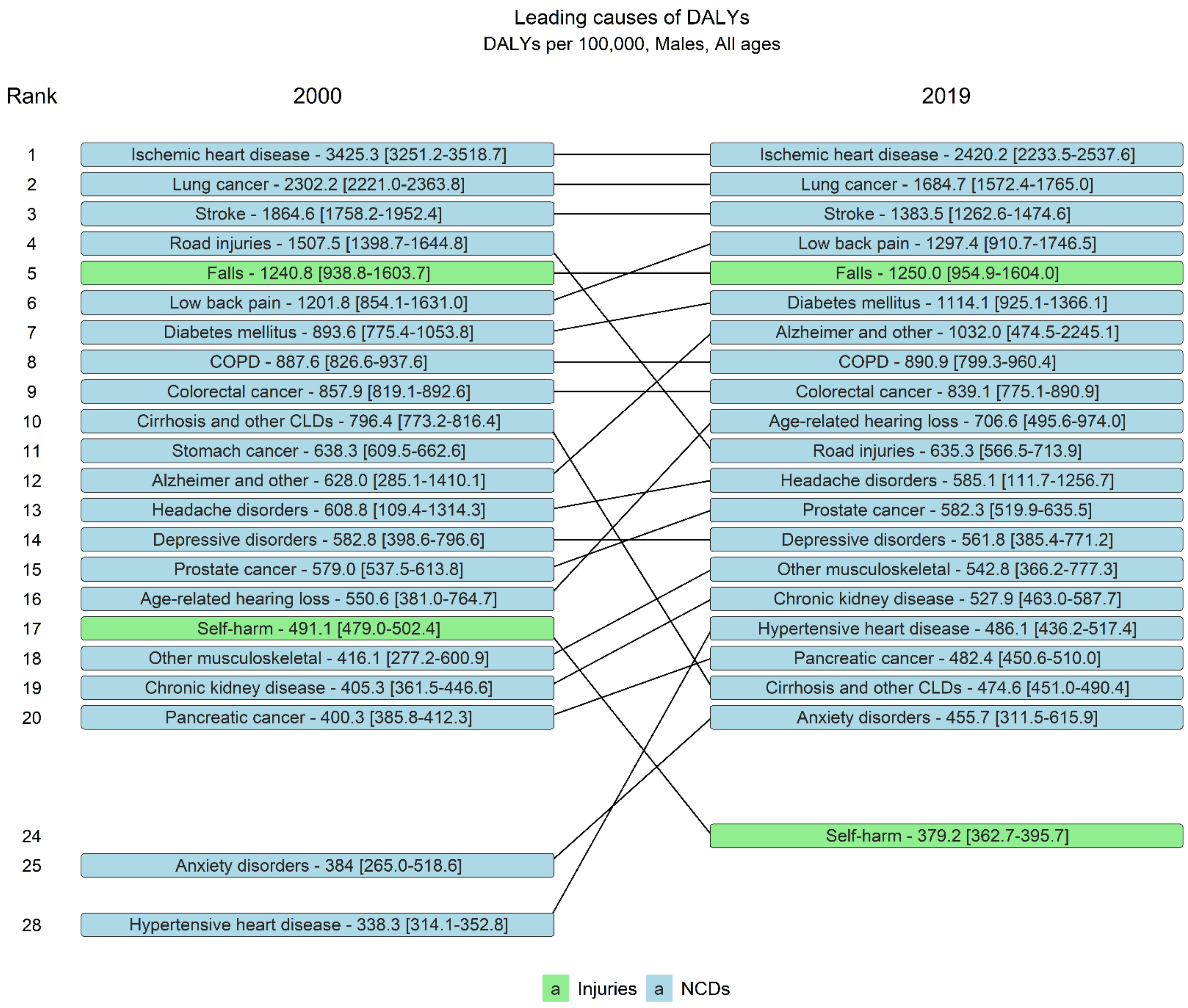

Abbreviations: COPD: Chronic Obstructive Pulmonary Disease; CLDs: Chronic Liver Diseases; NCDs: Non-Communicable Diseases.

Figure S19b. Leading causes of all-age Disability-Adjusted Life-Years in Italy in 2000 and 2019 - Females.

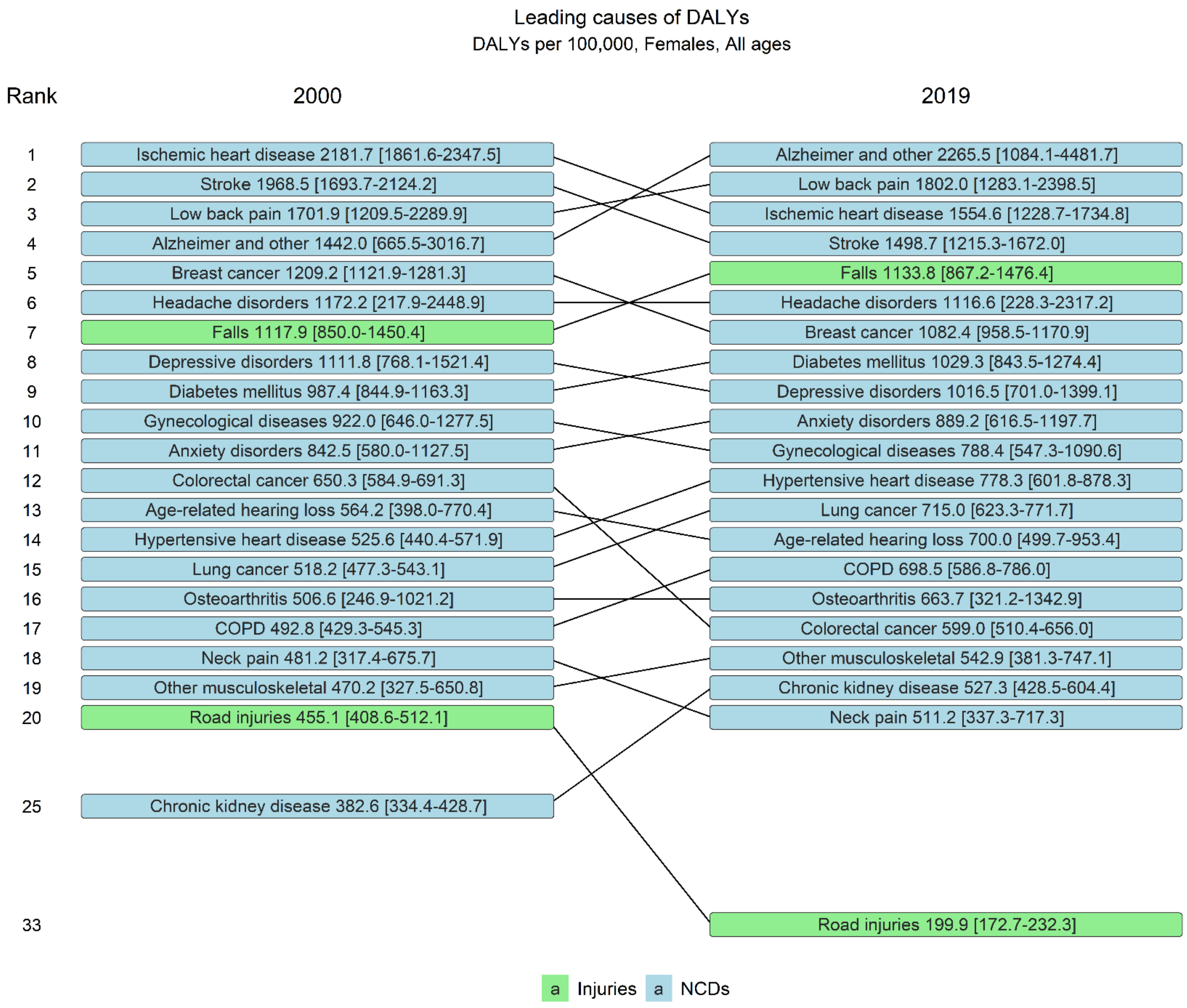

Abbreviations: COPD: Chronic Obstructive Pulmonary Disease; NCDs: Non-Communicable Diseases.

Figure S20. Trends of age-standardised Disability-Adjusted Life-Years (DALYs) by region in Italy between 2000 and 2021 for females (a) and males (b).

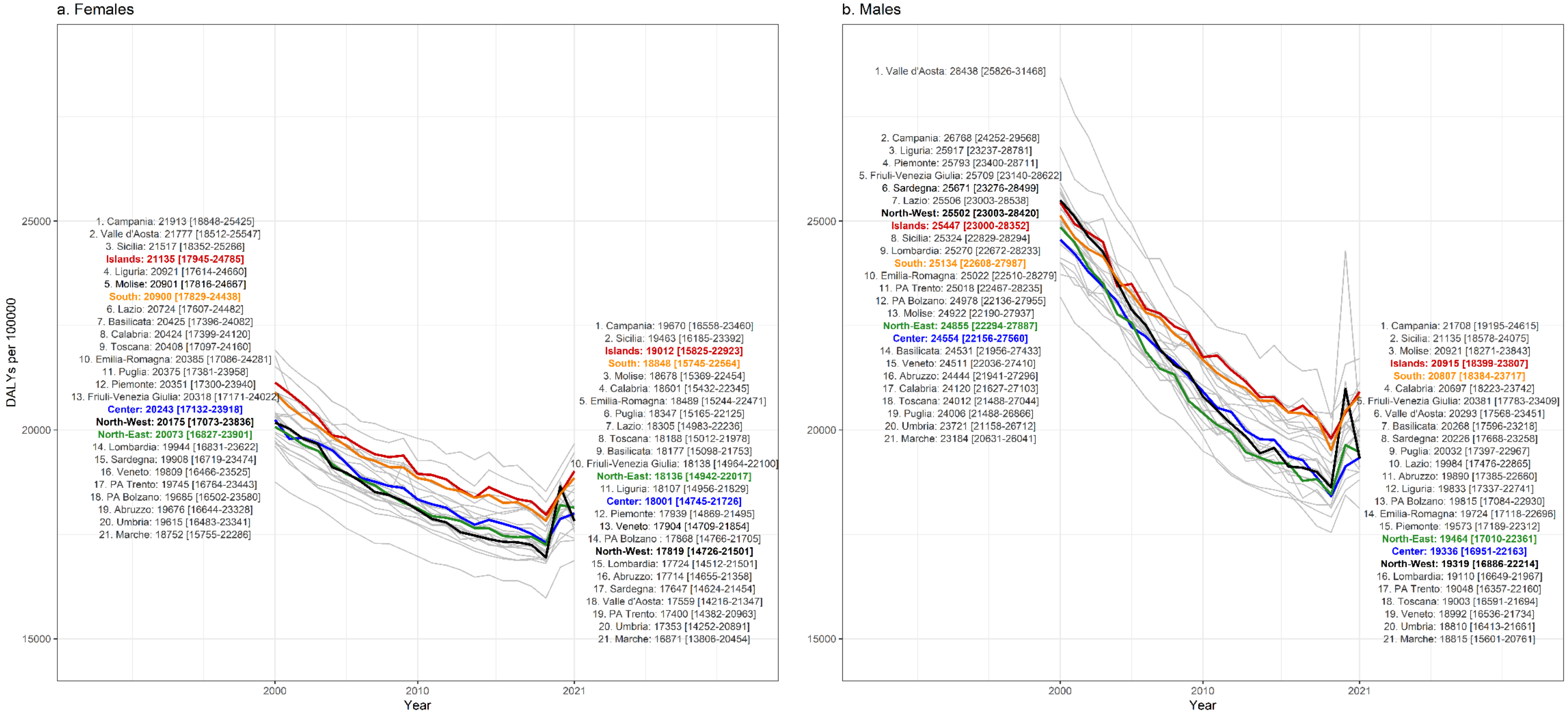

Figure S21. Leading causes of age-standardised Disability-Adjusted Life-Years (DALYs) in Italy in 2000 and 2019 – Both sexes.

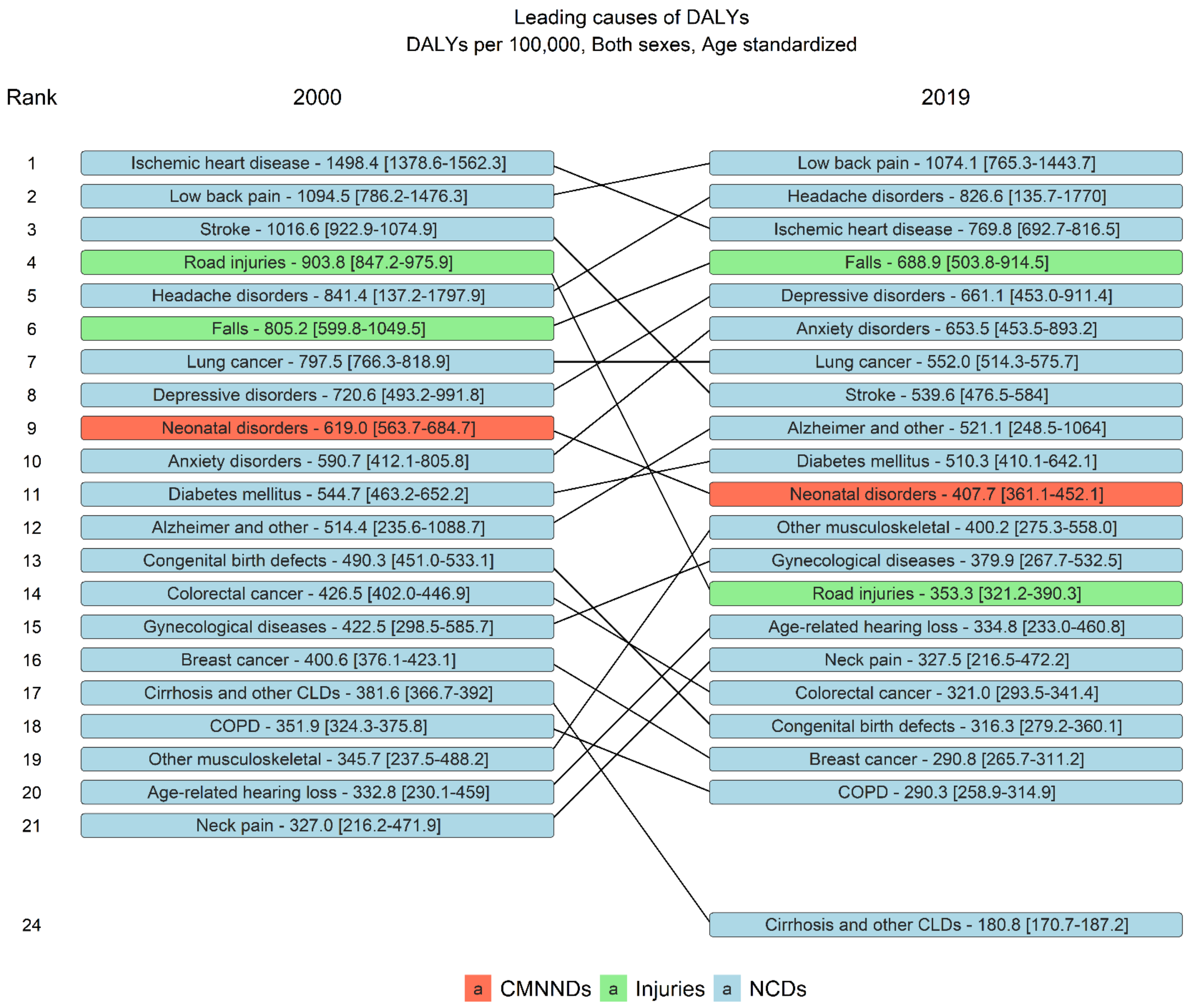

Abbreviations: CLDs: Chronic Liver Diseases; COPD: Chronic Obstructive Pulmonary Disease; CMNNDs: Communicable, Maternal, Neonatal and Nutritional Diseases; NCDs: Non-Communicable Diseases.

Figure S22a. Leading causes of age-standardised Disability-Adjusted Life-Years (DALYs) in Italy in 2000 and 2019 –Males.

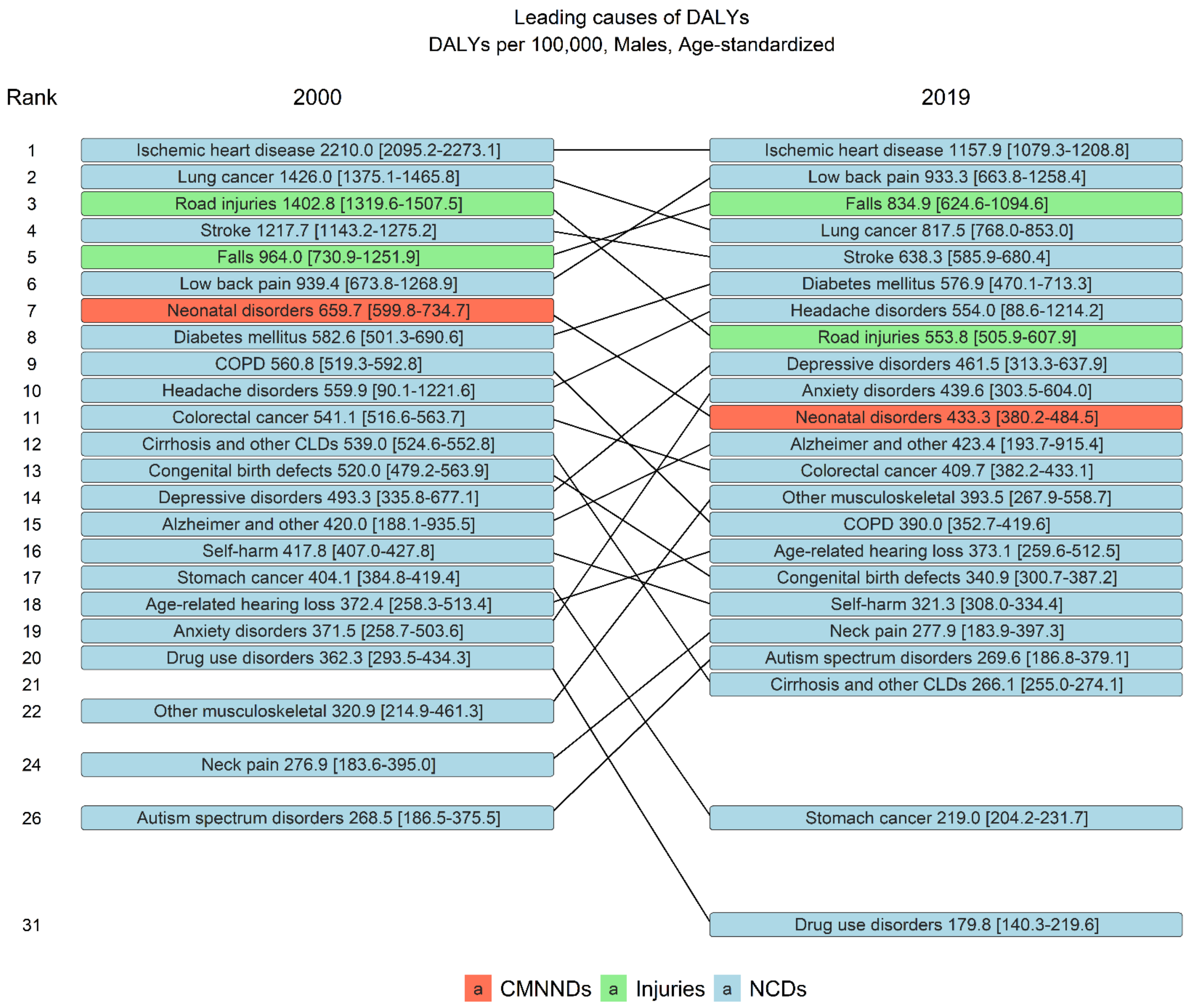

Abbreviations: COPD: Chronic Obstructive Pulmonary Disease; CLDs: Chronic Liver Diseases; CMNNDs: Communicable, Maternal, Neonatal and Nutritional Diseases; NCDs: Non-Communicable Diseases.

Figure S22b. Leading causes of age-standardised Disability-Adjusted Life-Years (DALYs) in Italy in 2000 and 2019 – Females.

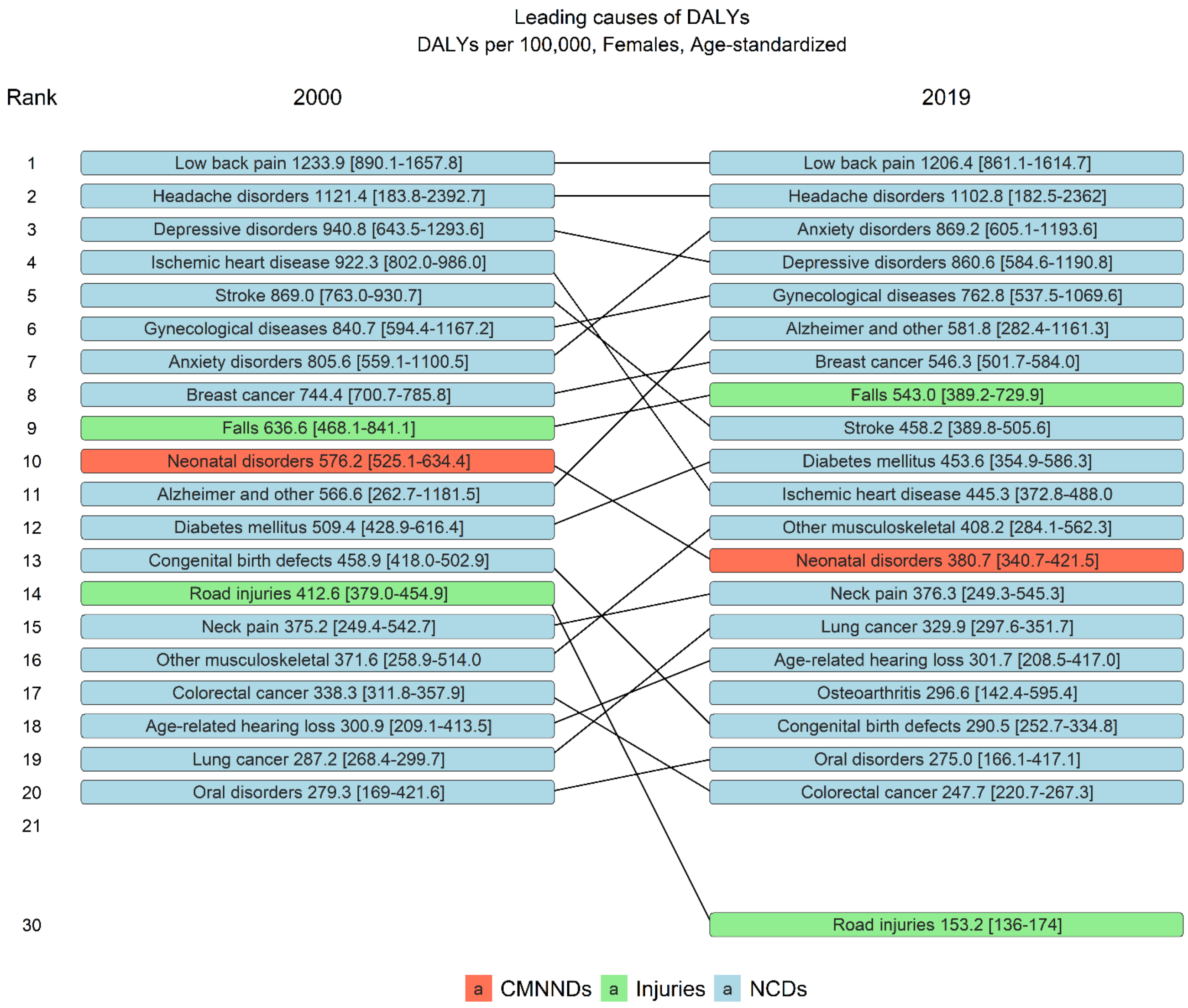

Abbreviations: CMNNDs: Communicable, Maternal, Neonatal and Nutritional Diseases; NCDs: Non-Communicable Diseases.

Figure S23. Ten leading causes of age-standardised Disability-Adjusted Life-Years (DALYs) in 2019 in Italy and in the five macro-regions. Rate are expressed per 100,000 people.

Italy

Rank 2019	Cause	DALY rate (95% UI)	Change from 2000 to 2019	Change from 2019 to 2021
1	Low back pain	1074.1 (765.3-1443.7)	1.9%	0.8%
2	Headache disorders	826.6 (135.7-1770.0)	-1.8%	-4.6%
3	Ischemic heart disease	768.9 (692.7-816.5)	-48.6%	-3.0%
4	Falls	688.9 (503.8-914.5)	-14.5%	-0.9%
5	Depressive disorders	661.1 (453.0-911.4)	-8.3%	23.1%
6	Anxiety disorders	653.5 (453.5-893.2)	10.7%	24.6%
7	Lung cancer	552.0 (514.3-575.7)	-30.8%	-3.7%
8	Stroke	539.6 (476.5-584.0)	-16.9%	-2.8%
9	Alzheimer and other	521.1 (248.5-1064.0)	1.3%	-2.6%
10	Diabetes mellitus	510.3 (410.1-642.1)	-6.3%	3.0%

North-West

Rank 2019	Cause	DALY rate (95% UI)	Change from 2000 to 2019	Change from 2019 to 2021
1	Low back pain	1083.2 (774.3-1457.5)	-1.8%	0.7%
2	Headache disorders	841.8 (139.5-1812.5)	-1.7%	-4.6%
3	Falls	723.5 (524.9-968.9)	-16.5%	-1.1%
4	Ischemic heart disease	702.2 (629.5-749.8)	-51.1%	-4.6%
5	Anxiety disorders	657.4 (456.3-895.6)	11.4%	25.1%
6	Depressive disorders	606.7 (418.7-836.9)	-9.5%	24.3%
7	Lung cancer	565.5 (521.0-602.0)	-34.8%	-3.9%
8	Alzheimer and other	526.2 (251.3-1069.9)	0.8%	-2.8%
9	Stroke	526.1 (461.0-575.0)	-46.9%	-3.9%
10	Other musculoskeletal	405.2 (280.0-564.3)	17.7%	3.1%

North-East

Rank 2019	Cause	DALY rate (95% UI)	Change from 2000 to 2019	Change from 2019 to 2021
1	Low back pain	1088.1 (774.9-1456.8)	-1.8%	0.7%
2	Headache disorders	840.4 (135.3-1808.8)	-1.5%	-4.6%
3	Depressive disorders	803.7 (545.5-1116.6)	-3.9%	22.1%
4	Falls	725.8 (526.5-972.8)	-12.4%	-0.9%
5	Ischemic heart disease	663.8 (589.6-710.4)	-53.8%	-5.4%
6	Anxiety disorders	655.7 (456.7-899.7)	11.7%	22.7%
7	Alzheimer and other	536.2 (261.0-1086.5)	0.1%	-2.5%
8	Lung cancer	507.3 (466.3-537.5)	-38.4%	-7.6%
9	Diabetes mellitus	466.0 (368.4-593.4)	5.8%	3.6%
10	Stroke	447.2 (389.1-487.7)	-44.4%	-4.3%

Center

Rank 2019	Cause	DALY rate (95% UI)	Change from 2000 to 2019	Change from 2019 to 2021
1	Low back pain	1069.5 [764.2-1440.6]	-1.7%	1.2%
2	Headache disorders	825.6 [136.7-1768.6]	-1.8%	23.0%
3	Ischemic heart disease	759.7 [683.8-808.1]	-49.6%	-2.8%
4	Depressive disorders	680.7 [463.4-934]	-10.7%	23.0%
5	Falls	677.1 [498.7-884.2]	-16.1%	-0.7%
6	Anxiety disorders	662.1 [457.4-910.5]	8.2%	24.0%
7	Lung cancer	566.5 [523.9-600.5]	-28.0%	-4.0%
8	Stroke	522.1 [463.9-564.6]	-47.1%	-3.0%
9	Alzheimer and other	492.1 [235.3-1003.6]	2.7%	-2.9%
10	Diabetes mellitus	467.5 [372.5-595.8]	-6.3%	2.8%

South

Rank 2019	Cause	DALY rate (95% UI)	Change from 2000 to 2019	Change from 2019 to 2021
1	Low back pain	1096.3 [789.9-1470.3]	-1.9%	0.7%
2	Ischemic heart disease	932.2 [679.2-1246.2]	-42.2%	-1.2%
3	Headache disorders	819.7 [134.9-1765.3]	-2.1%	-4.9%
4	Falls	656.6 [575.4-712.8]	-8.7%	-0.7%
5	Anxiety disorders	655.1 [455.9-889.2]	11.0%	25.0%
6	Diabetes mellitus	602.8 [410.6-825.7]	-10.3%	3.2%
7	Depressive disorders	494.7 [243.5-989.1]	-9.7%	22.5%
8	Stroke	444.9 [400.5-491.4]	-49.1%	-1.5%
9	Lung cancer	433.7 [377.5-477.6]	-24.5%	-1.4%
10	Alzheimer and other	405.7 [285.0-570.7]	2.1%	-2.1%

Islands

Rank 2019	Cause	DALY rate (95% UI)	Change from 2000 to 2019	Change from 2019 to 2021
1	Low back pain	1087.7 [782.7-1465.1]	-2.1%	0.6%
2	Ischemic heart disease	908.6 [649.3-1223.4]	-45.1%	-0.6%
3	Diabetes mellitus	823.9 [137.5-1767.9]	-4.4%	3.4%
4	Headache disorders	659.2 [567.3-717.7]	-2.1%	-4.9%
5	Stroke	656.3 [459.8-894.6]	-49.0%	-1.0%
6	Depressive disorders	605.8 [408.9-833.7]	-8.5%	23.5%
7	Anxiety disorders	506.2 [245.7-1024]	10.0%	26.5%
8	Alzheimer and other	474.2 [425.9-520.3]	1.4%	-2.9%
9	Lung cancer	420.8 [326.6-550.1]	-20.6%	-1.0%
10	Falls	378.4 [267.0-535.3]	-23.1%	-1.0%

NCDs **CMNNDs** **Injuries**

Change 2000: DALY rate change (%) from 2000 to 2019; Change 2021: DALY rate change (%) from 2019 to 2021. Abbreviations: NCDs: Non-Communicable Diseases; CMNNDs: Communicable, Maternal, Neonatal, Nutritional Disorders.

Table S5. Ten leading causes of all-age Disability-Adjusted Life-Years (DALYs) in 2019 by region.

Region	Rank (2019)	Cause	DALYs per 100000 [95% UI]	Change 2000 (%)	Change 2021 (%)
Piemonte - North-West	1	Ischemic heart disease	1992.8 [1731.2-2154.7]	-29.4	0.4
	2	Stroke	1854.5 [1577.8-2041]	-23.9	0.2
	3	Alzheimer and other	1844.5 [871.3-3762.8]	10.3	-0.6
	4	Low back pain	1646.6 [1163.5-2199]	4.5	1.9
	5	Lung cancer	1332.2 [1204-1445.5]	7.5	23.0
	6	Falls	1096.9 [849.9-1392.3]	-6.5	22.2
	7	Diabetes mellitus	962.9 [797.7-1192.4]	-14.0	14.9
	8	COPD	926.1 [808.3-1015.6]	3.8	8.4
	9	Headache disorders	842.1 [173.6-1767]	-5.3	11.5
	10	Colorectal cancer	815.6 [713.6-917.5]	-4.2	6.5
Valle d'Aosta - North-West	1	Ischemic heart disease	2010.4 [1726.6-2209.3]	-32.8	1.6
	2	Alzheimer and other	1686.2 [792.8-3459]	-19.8	-0.4
	3	Low back pain	1603.3 [1147.1-2157.7]	5.2	2.2
	4	Stroke	1549.3 [1280.6-1746.3]	2.3	2.6
	5	Falls	1423.2 [1110.2-1804.7]	-4.8	7.4
	6	Lung cancer	1173.6 [1026.1-1329.6]	2.2	22.7
	7	Diabetes mellitus	942.1 [753.8-1179.6]	-9.4	24.5
	8	COPD	911.3 [787.3-1004.2]	1.7	10.1
	9	Headache disorders	844.4 [173.1-1760.9]	-1.0	9.9
	10	Age-related hearing loss	730 [516.6-994.1]	-13.3	15.5

Region	Rank (2019)	Cause	DALYs per 100000 [95% UI]	Change 2000 (%)	Change 2021 (%)
Lombardia - North-West	1	Ischemic heart disease	1770.4 [1509.8-1925.2]	-34.4	-1.1
	2	Alzheimer and other	1710.9 [806.8-3423.6]	3.4	1.9
	3	Low back pain	1554.1 [1103-2068.8]	1.9	11.4
	4	Stroke	1296.1 [1085.3-1432.1]	-12.6	22.3
	5	Falls	1279.6 [957.2-1658.5]	-0.7	0.6
	6	Lung cancer	1231.8 [1113-1333.8]	15.3	4.3
	7	Headache disorders	880.1 [173.6-1873.2]	-11.3	38.2
	8	Depressive disorders	724.9 [499.6-992.9]	-23.0	17.9
	9	COPD	714.8 [622.3-789.8]	-11.3	18.8
	10	Age-related hearing loss	700.7 [500.1-963.9]	-8.1	16.3
AP Bolzano - North-East	1	Ischemic heart disease	1543.9 [1320-1695.5]	-37.0	53.4
	2	Low back pain	1506.2 [1078.8-2010.6]	7.7	5.2
	3	Falls	1453.7 [1084-1892.9]	4.1	5.7
	4	Alzheimer and other	1418 [691.3-2846.8]	5.5	4.3
	5	Stroke	1048.8 [890.2-1161.2]	-1.1	36.6
	6	Lung cancer	850.8 [758.8-942.8]	-11.3	29.1
	7	Headache disorders	847.6 [163.1-1794.5]	-4.5	-4.5
	8	Depressive disorders	705.2 [488.7-958.9]	-17.6	13.9
	9	Diabetes mellitus	687.2 [539.8-879.6]	-10.5	16.9
	10	Anxiety disorders	675.4 [460.9-907.4]	-2.0	16.6
AP Trento - North-East	1	Ischemic heart disease	1827.1 [1500.7-2022.2]	-42.7	9.8
	2	Alzheimer and other	1697.1 [808-3393.8]	15.6	9.3
	3	Low back pain	1546.6 [1102.6-2076.4]	7.7	8.0
	4	Falls	1457.8 [1090.6-1910.7]	10.9	8.5
	5	Stroke	1020.1 [855.1-1133.7]	-21.6	44.0
	6	Lung cancer	991.8 [871.4-1092.6]	-3.1	4.5
	7	Headache disorders	845.7 [170-1790.1]	-8.2	11.4
	8	Diabetes mellitus	841.3 [675.1-1071.6]	-5.3	5.6
	9	Depressive disorders	719.1 [496.3-980.5]	-10.1	14.9
	10	Age-related hearing loss	694.5 [493.5-948]	-11.9	15.7

Region	Rank (2019)	Cause	DALYs per 100000 [95% UI]	Change 2000 (%)	Change 2021 (%)
Veneto - North East	1	Ischemic heart disease	1753 [1507.7-1908.6]	-31.0	15.8
	2	Alzheimer and other	1640.2 [776-3359.5]	9.4	5.7
	3	Low back pain	1572 [1108.9-2101.9]	5.4	6.6
	4	Stroke	1202.4 [1025.5-1323.9]	-19.2	33.9
	5	Falls	1143.3 [870.6-1470.5]	-1.7	4.5
	6	Lung cancer	1062.7 [946.5-1155.2]	-6.8	8.4
	7	Diabetes mellitus	1004.1 [808.6-1253]	-4.4	6.4
	8	Depressive disorders	914.7 [614.7-1263.4]	-4.5	15.1
	9	Headache disorders	851.7 [171.1-1791.2]	-5.7	20.8
	10	Age-related hearing loss	703.3 [495.3-956.6]	-7.0	14.8
Friuli-Venezia Giulia - North-East	1	Ischemic heart disease	2156.8 [1858.8-2361.8]	-37.1	58.1
	2	Alzheimer and other	1911.6 [907.6-3897.3]	1.5	10.6
	3	Low back pain	1644.8 [1184.8-2220.3]	-0.3	15.0
	4	Stroke	1499.8 [1254-1661]	-5.1	11.7
	5	Falls	1278.5 [982.1-1630.7]	-5.1	15.5
	6	Lung cancer	1225.6 [1100-1335.8]	-4.7	4.9
	7	Diabetes mellitus	1041.1 [841.8-1273.2]	-6.7	13.9
	8	Osteoarthritis	871.4 [424.7-1750]	-12.1	26.1
	9	Headache disorders	835.1 [172.9-1747.3]	-9.5	6.7
	10	COPD	826.5 [716.7-912.8]	-7.0	4.1
Liguria - North-West	1	Ischemic heart disease	2347 [1987.6-2559.4]	-38.7	-1.9
	2	Alzheimer and other	2245 [1054.2-4576.6]	-8.9	0.6
	3	Stroke	1933 [1638.4-2140.1]	0.9	-1.7
	4	Low back pain	1684.4 [1189.4-2251.3]	0.6	5.4
	5	Falls	1623.7 [1246.7-2078.1]	-1.4	5.4
	6	Lung cancer	1432.4 [1288.8-1569.6]	-6.0	13.5
	7	Diabetes mellitus	1135.2 [937.4-1382.8]	0.3	22.6
	8	COPD	964.1 [839.6-1065.5]	-13.8	22.1
	9	Colorectal cancer	931.6 [811.3-1038.6]	5.0	3.2
	10	Age-related hearing loss	850.3 [609.9-1156.8]	-2.0	6.6

Region	Rank (2019)	Cause	DALYs per 100000 [95% UI]	Change 2000 (%)	Change 2021 (%)
Emilia-Romagna - North-East	1	Alzheimer and other	2046.9 [993.9-4102.6]	-39.0	10.8
	2	Ischemic heart disease	1833.8 [1550.3-2025.1]	-3.4	10.9
	3	Low back pain	1596.4 [1130.8-2128]	-1.5	12.9
	4	Stroke	1329.6 [1095.8-1479.9]	-16.3	22.2
	5	Falls	1306.5 [991.8-1686.1]	-15.1	2.9
	6	Lung cancer	1205.2 [1064.7-1325.7]	-10.4	8.8
	7	Depressive disorders	1140.7 [785.2-1551.9]	-8.5	14.8
	8	Diabetes mellitus	995.5 [809.4-1225]	-16.2	15.3
	9	Headache disorders	896.6 [174.6-1913.8]	-4.5	15.1
	10	COPD	764.3 [651.2-847.4]	-14.6	12.9
Toscana - Centre	1	Ischemic heart disease	1983.9 [1728.1-2139.6]	-38.0	0.1
	2	Stroke	1771.4 [1511.6-1939.3]	-27.6	-0.4
	3	Alzheimer and other	1745 [821.5-3565.7]	11.0	1.1
	4	Low back pain	1616.3 [1144-2163.9]	6.0	8.0
	5	Lung cancer	1271.8 [1156.9-1389.4]	3.9	29.6
	6	Falls	1127.7 [885-1433.4]	-4.4	10.9
	7	Diabetes mellitus	1044.6 [858.6-1294.5]	-9.1	11.9
	8	Depressive disorders	1011 [690.3-1381.7]	6.7	12.6
	9	Headache disorders	843.1 [174.2-1769.2]	-9.5	28.9
	10	COPD	840.6 [738.9-922.3]	-8.0	9.2
Umbria - Centre	1	Ischemic heart disease	2280 [1900-2499]	-35.8	-2.0
	2	Alzheimer and other	1927.7 [887.7-3940.1]	-16.3	-1.4
	3	Low back pain	1611.3 [1146.2-2152.2]	2.2	2.2
	4	Stroke	1602.6 [1352.6-1776.3]	25.8	-2.3
	5	Falls	1292.1 [985-1671.6]	3.9	21.0
	6	Lung cancer	1091.6 [969.7-1206.9]	-10.7	18.9
	7	Diabetes mellitus	908.9 [753.4-1105.4]	-6.6	14.8
	8	Headache disorders	848.3 [173.6-1777.4]	-3.8	9.4
	9	COPD	834.5 [715.5-924.2]	-5.0	2.0
	10	Age-related hearing loss	762.1 [542-1037.1]	-12.7	8.2

Region	Rank (2019)	Cause	DALYs per 100000 [95% UI]	Change 2000 (%)	Change 2021 (%)
Marche – Centre	1	Ischemic heart disease	2155.5 [1798.2-2380.5]	-26.2	1.6
	2	Alzheimer and other	1869.5 [876.4-3820.7]	-11.4	1.3
	3	Low back pain	1455.1 [1012.9-2035.1]	4.1	18.7
	4	Stroke	1449.9 [1216.3-1624.2]	12.4	5.8
	5	Falls	1122.3 [870.6-1418.5]	-9.6	31.5
	6	Lung cancer	1013.7 [890.6-1123.3]	-11.1	12.1
	7	Headache disorders	852.2 [174.7-1785.1]	-18.0	17.5
	8	Diabetes mellitus	845 [693.3-1046.3]	-4.6	4.7
	9	COPD	772.7 [658.8-858.3]	-7.0	10.7
	10	Age-related hearing loss	755.5 [540.3-1030.2]	-6.4	7.2
Lazio - Centre	1	Ischemic heart disease	2059.6 [1791-2237]	-28.7	2.5
	2	Low back pain	1560 [1114.7-2087.1]	-7.6	9.4
	3	Alzheimer and other	1492.1 [718-3060.3]	-0.1	7.2
	4	Falls	1328.8 [1011.7-1717.5]	-9.5	14.1
	5	Lung cancer	1326.2 [1193.7-1435.4]	0.4	1.9
	6	Stroke	1258.9 [1093.5-1375.9]	27.3	5.5
	7	Diabetes mellitus	1048.8 [858.5-1288.6]	6.3	22.4
	8	Headache disorders	862.9 [174.8-1815.8]	-5.3	28.3
	9	COPD	748.6 [654.6-820.6]	-16.1	16.3
	10	Depressive disorders	735.5 [503.7-1016.3]	-9.2	12.2
Abruzzo - South	1	Ischemic heart disease	2453.1 [2112.3-2670.1]	-6.4	1.3
	2	Alzheimer and other	1726.1 [821.9-3530.2]	-11.6	3.5
	3	Low back pain	1581.8 [1124.9-2106]	6.8	10.3
	4	Stroke	1442.5 [1231.9-1589.6]	5.3	12.2
	5	Falls	1352.2 [1045.5-1726.3]	21.8	7.6
	6	Diabetes mellitus	1110.6 [914-1355.8]	1.8	23.2
	7	Lung cancer	986.1 [885.1-1079]	-1.3	18.5
	8	COPD	828.5 [720.2-908.1]	-14.7	18.6
	9	Headache disorders	804.9 [158.6-1705.1]	-5.3	4.8
	10	Hypertensive heart disease	778.8 [632.5-866.8]	-1.9	6.7

Region	Rank (2019)	Cause	DALYs per 100000 [95% UI]	Change 2000 (%)	Change 2021 (%)
Molise – South	1	Ischemic heart disease	2883.9 [2516.2-3168.4]	-13.3	-2.3
	2	Alzheimer and other	1849.2 [870-3876]	-21.6	20.6
	3	Stroke	1792.1 [1567.1-1965.9]	21.6	3.3
	4	Low back pain	1590.7 [1128.4-2109.6]	12.6	10.3
	5	Falls	1472.4 [1134.2-1891.1]	29.0	10.4
	6	Diabetes mellitus	1347.4 [1120.4-1645.7]	21.4	9.5
	7	Lung cancer	1057.6 [944.5-1165.6]	9.2	31.7
	8	Hypertensive heart disease	928 [779.1-1035]	-1.6	9.5
	9	COPD	888.4 [779.5-983.5]	1.3	2.7
	10	Headache disorders	851.5 [173.1-1778.6]	8.1	3.0
Campania - South	1	Ischemic heart disease	2226.1 [1985-2390.4]	-17.4	3.6
	2	Stroke	1486.4 [1305.5-1612.5]	-25.7	29.4
	3	Low back pain	1462.1 [1028.8-1959.4]	12.6	4.9
	4	Diabetes mellitus	1296.9 [1092.7-1563.7]	3.6	15.8
	5	Lung cancer	1266.6 [1160.6-1366.4]	14.7	8.3
	6	Alzheimer and other	1212.5 [570.7-2523.8]	34.9	7.7
	7	Falls	1037.7 [782.7-1341.2]	15.5	20.9
	8	Headache disorders	877.7 [171.2-1837.2]	2.5	20.4
	9	COPD	852.1 [762.6-928]	12.5	3.3
	10	Depressive disorders	718.3 [492.1-986.2]	-2.0	18.7
Puglia - South	1	Ischemic heart disease	1820.2 [1579-1976.9]	-14.4	27.2
	2	Low back pain	1517.3 [1073.6-2035.8]	2.8	25.9
	3	Alzheimer and other	1484.5 [690.1-3075.2]	10.0	4.7
	4	Falls	1151.8 [880.9-1485.6]	6.9	34.0
	5	Stroke	1132.9 [970.3-1244.9]	10.1	7.9
	6	Diabetes mellitus	1129.4 [936.2-1365.7]	15.2	4.9
	7	Lung cancer	1002.9 [906-1086.3]	11.0	17.6
	8	Headache disorders	868.4 [172.4-1829.5]	-3.1	19.2
	9	COPD	859.2 [749.1-936]	4.3	4.3
	10	Hypertensive heart disease	830.6 [677.4-924.1]	7.2	4.5

Region	Rank (2019)	Cause	DALYs per 100000 [95% UI]	Change 2000 (%)	Change 2021 (%)
Basilicata – South	1	Ischemic heart disease	2242.9 [1962.5-2448.2]	-12.3	2.2
	2	Alzheimer and other	1706.4 [807-3519.9]	-16.9	1.0
	3	Stroke	1593.4 [1386.7-1763.2]	13.1	1.3
	4	Low back pain	1569.5 [1114.3-2089.8]	47.1	2.6
	5	Diabetes mellitus	1357.4 [1126.4-1645.2]	32.1	5.5
	6	Hypertensive heart disease	1156 [957.4-1290]	23.9	4.2
	7	Falls	1153.7 [896.9-1469.3]	27.4	1.6
	8	COPD	1032.5 [898.2-1138.8]	15.6	13.5
	9	Lung cancer	950.6 [862.7-1043.8]	6.8	9.9
	10	Headache disorders	860.4 [178.2-1815]	9.1	12.9
Calabria - South	1	Ischemic heart disease	2180.1 [1926.1-2350]	-12.1	2.0
	2	Stroke	1541.1 [1339.1-1676.8]	-20.5	8.5
	3	Low back pain	1496.3 [1051.1-2009.7]	10.8	4.6
	4	Alzheimer and other	1477.4 [687.8-3044.2]	39.8	3.7
	5	Diabetes mellitus	1355.8 [1126.2-1650.1]	29.4	9.4
	6	Falls	1131.7 [876.7-1437.7]	26.7	26.2
	7	Hypertensive heart disease	1024.4 [858.4-1133.8]	16.2	12.0
	8	Lung cancer	967.9 [888.2-1045.8]	15.1	7.3
	9	Headache disorders	867 [171.3-1826.4]	12.5	12.7
	10	COPD	821.3 [728.3-898]	9.7	3.0
Sicilia - Islands	1	Ischemic heart disease	2036.7 [1784.8-2194.2]	-20.7	3.6
	2	Diabetes mellitus	1712.4 [1381.7-2108]	-26.1	20.2
	3	Stroke	1711.1 [1469.7-1872.8]	21.8	5.6
	4	Alzheimer and other	1631.5 [771.8-3333.1]	20.6	8.3
	5	Low back pain	1484.4 [1048.1-1999.7]	40.3	11.6
	6	Lung cancer	1108.4 [1008.2-1198.4]	10.3	37.1
	7	Falls	905.6 [713.8-1140.2]	-4.0	25.4
	8	COPD	849.7 [741.3-926.8]	2.9	8.5
	9	Hypertensive heart disease	834.1 [694.9-930.7]	8.4	5.1
	10	Headache disorders	804.8 [171.6-1689]	6.5	7.9

Region	Rank (2019)	Cause	DALYs per 100000 [95% UI]	Change 2000 (%)	Change 2021 (%)
Sardegna – Islands	1	Ischemic heart disease	1983.9 [1728.1-2139.6]	-23.4	5.4
	2	Stroke	1771.4 [1511.6-1939.3]	-3.4	3.1
	3	Alzheimer and other	1745 [821.5-3565.7]	14.5	2.7
	4	Low back pain	1616.3 [1144-2163.9]	19.2	5.6
	5	Lung cancer	1271.8 [1156.9-1389.4]	22.5	10.1
	6	Falls	1127.7 [885-1433.4]	24.7	3.2
	7	Diabetes mellitus	1044.6 [858.6-1294.5]	13.8	12.4
	8	Depressive disorders	1011 [690.3-1381.7]	-6.8	34.0
	9	Headache disorders	843.1 [174.2-1769.2]	-1.4	12.7
	10	COPD	840.6 [738.9-922.3]	-1.5	3.7

Change 2000: DALY rate change (%) from 2000 to 2019; Change 2021: DALY rate change (%) from 2019 to 2021.
Abbreviations: COPD: Chronic obstructive pulmonary disease; UI: Uncertainty Intevral.

Table S6. Ten leading causes of all-age Disability-Adjusted Life-Years (DALYs) in 2019 by region.

Region	Rank (2019)	Cause	DALYs per 100000 [95% UI]	Change 2000 (%)	Change 2021 (%)
Piemonte - North-West	1	Low back pain	1101.6 [776-1471.5]	-18.1	0.6
	2	Headache disorders	826.5 [135.8-1773.9]	-26.2	0.6
	3	Ischemic heart disease	720.4 [643.7-772]	-35.5	9.1
	4	Anxiety disorders	658.3 [453-896.4]	-39.1	16.0
	5	Depressive disorders	608 [414.2-845.3]	-29.5	21.1
	6	Stroke	598 [523.3-654.1]	-28.8	17.0
	7	Falls	578.6 [432.7-756.2]	-24.6	0.5
	8	Lung cancer	568.1 [517.4-613.7]	-15.3	0.8
	9	Alzheimer and other	508.8 [244.5-1034.4]	-18.9	9.5
	10	Neonatal disorders	437.3 [369.4-511.6]	-25.9	12.1
Valle d'Aosta - North-West	1	Low back pain	1091 [784.4-1460.6]	-30.2	0.6
	2	Headache disorders	825.6 [136.1-1771]	-25.7	-2.8
	3	Falls	793.3 [599-1028.4]	-26.9	-1.0
	4	Ischemic heart disease	765.5 [682.8-837.6]	-28.2	2.6
	5	Anxiety disorders	657.5 [452-903.1]	-34.6	13.2
	6	Depressive disorders	606.3 [414.8-828.4]	-27.9	21.8
	7	Stroke	534.2 [459.2-596.5]	-35.9	32.9
	8	Lung cancer	529.6 [463.9-602.5]	-20.5	-0.7
	9	Alzheimer and other	497.5 [236.4-1034.1]	-15.2	2.2
	10	Diabetes mellitus	448.3 [349.1-572.7]	-19.0	7.1

Region	Rank (2019)	Cause	DALYs per 100000 [95% UI]	Change 2000 (%)	Change 2021 (%)
Lombardia - North-West	1	Low back pain	1075.2 [764-1447.2]	-26.8	0.7
	2	Headache disorders	850.9 [142.5-1830.1]	-22.4	-2.4
	3	Falls	775.7 [558.4-1049.1]	-17.8	5.8
	4	Ischemic heart disease	692.2 [611.7-742.1]	-23.2	18.2
	5	Anxiety disorders	656.9 [454.1-893]	-26.7	16.7
	6	Depressive disorders	606.2 [418.5-832.5]	-30.2	23.9
	7	Lung cancer	564.8 [516.8-609]	-34.9	16.0
	8	Alzheimer and other	539.1 [257.7-1089.9]	-19.6	0.1
	9	Stroke	486.3 [422.1-532.7]	-17.6	8.1
	10	Other musculoskeletal	395.6 [270.1-553.4]	-27.4	17.3
AP Bolzano - North-East	1	Low back pain	1096.3 [789.9-1470.3]	-27.1	15.8
	2	Falls	932.2 [679.2-1246.2]	-16.5	18.2
	3	Headache disorders	819.7 [134.9-1765.3]	-23.4	13.4
	4	Ischemic heart disease	656.6 [575.4-712.8]	-35.6	19.6
	5	Anxiety disorders	655.1 [455.9-889.2]	-21.4	19.0
	6	Depressive disorders	602.8 [410.6-825.7]	-26.3	17.9
	7	Alzheimer and other	494.7 [243.5-989.1]	-25.8	28.3
	8	Lung cancer	444.9 [400.5-491.4]	-29.4	8.5
	9	Stroke	433.7 [377.5-477.6]	-26.1	-1.1
	10	Gynaecological diseases	405.7 [285-570.7]	-28.9	-0.7
AP Trento - North-East	1	Low back pain	1087.7 [782.7-1465.1]	-34.9	0.8
	2	Falls	908.6 [649.3-1223.4]	-18.0	9.0
	3	Headache disorders	823.9 [137.5-1767.9]	-10.9	9.4
	4	Ischemic heart disease	659.2 [567.3-717.7]	-23.3	18.9
	5	Anxiety disorders	656.3 [459.8-894.6]	-21.5	19.0
	6	Depressive disorders	605.8 [408.9-833.7]	-21.0	19.6
	7	Alzheimer and other	506.2 [245.7-1024]	-32.3	25.9
	8	Lung cancer	474.2 [425.9-520.3]	-29.2	3.4
	9	Diabetes mellitus	420.8 [326.6-550.1]	-28.6	3.4
	10	Gynaecological diseases	378.4 [267.1-535.3]	-22.7	13.0

Region	Rank (2019)	Cause	DALYs per 100000 [95% UI]	Change 2000 (%)	Change 2021 (%)
Veneto - North East	1	Low back pain	1083.3 [767.5-1452.2]	-19.5	0.8
	2	Headache disorders	823.5 [135.7-1760.2]	-25.6	16.8
	3	Depressive disorders	762.4 [518-1063]	-29.3	20.1
	4	Falls	677.3 [493-901.4]	-20.6	17.5
	5	Ischemic heart disease	666 [589.4-717.2]	-20.3	17.5
	6	Anxiety disorders	656 [456.8-895.5]	-17.6	1.9
	7	Alzheimer and other	516.1 [245.2-1068.5]	-35.2	21.6
	8	Lung cancer	483.3 [437.6-523.8]	-38.5	4.8
	9	Diabetes mellitus	479 [375.2-610.4]	-18.4	4.3
	10	Stroke	443.7 [388.3-484]	-12.9	1.2
Friuli-Venezia Giulia - North-East	1	Low back pain	1087.5 [773-1465.5]	-28.3	30.1
	2	Headache disorders	822.6 [136.5-1768.3]	-25.8	33.0
	3	Ischemic heart disease	719.2 [637.9-779.8]	-31.6	15.0
	4	Falls	680.2 [504-893.7]	-19.1	15.0
	5	Anxiety disorders	656.3 [453.5-901.6]	-21.4	15.0
	6	Depressive disorders	604.8 [413.6-829.7]	-27.2	11.9
	7	Lung cancer	515.8 [465.9-560.1]	-36.6	30.5
	8	Alzheimer and other	502 [239.1-1029.1]	-24.7	-4.0
	9	Stroke	469.9 [406.9-515.4]	-20.1	2.5
	10	Diabetes mellitus	463.4 [363.8-586]	-5.9	3.3
Liguria - North-West	1	Low back pain	1082.7 [781.1-1445.1]	-27.7	0.6
	2	Headache disorders	825.9 [134.9-1771.6]	-25.0	-0.5
	3	Falls	774.6 [569.9-1017.4]	-17.6	1.4
	4	Ischemic heart disease	706.9 [626.5-760.3]	-24.4	7.8
	5	Anxiety disorders	658 [452.7-886.3]	-25.9	13.7
	6	Depressive disorders	606.6 [414.6-831.5]	-27.7	20.3
	7	Lung cancer	571 [520.6-622.6]	-31.2	17.5
	8	Stroke	549.8 [485.9-602.5]	-18.0	-1.8
	9	Alzheimer and other	508.9 [241.2-1031.9]	-20.5	3.7
	10	Diabetes mellitus	451.3 [361.9-567.6]	-23.7	10.2

Region	Rank (2019)	Cause	DALYs per 100000 [95% UI]	Change 2000 (%)	Change 2021 (%)
Emilia-Romagna - North-East	1	Low back pain	1092.8 [780.2-1474]	-25.9	7.6
	2	Depressive disorders	950 [644.9-1304.4]	-20.7	15.7
	3	Headache disorders	868.1 [136.1-1879.7]	-22.1	16.4
	4	Falls	744.3 [539.4-1003.2]	-23.8	11.9
	5	Anxiety disorders	655.1 [451.8-892.7]	-25.9	24.3
	6	Ischemic heart disease	648.5 [572.9-704.1]	-22.0	14.1
	7	Alzheimer and other	573.5 [283.4-1152.1]	-30.1	7.2
	8	Lung cancer	541.9 [488.4-589.2]	-33.4	3.2
	9	Diabetes mellitus	467.6 [366.9-595]	-20.3	7.0
	10	Stroke	455.2 [392.1-501]	-21.7	5.4
Toscana - Centre	1	Low back pain	1082 [777.3-1443.1]	-21.6	0.6
	2	Depressive disorders	837.6 [566.4-1145.2]	-23.9	22.0
	3	Headache disorders	825.6 [134.7-1763]	-19.8	11.8
	4	Anxiety disorders	758.2 [527.8-1042.2]	-19.7	3.6
	5	Ischemic heart disease	663.4 [595.3-708.8]	-25.0	16.4
	6	Stroke	560.6 [491.3-608.8]	-33.0	14.1
	7	Falls	554.6 [416.1-718.7]	-27.4	-0.8
	8	Lung cancer	546.1 [502.4-593.5]	-25.2	-0.9
	9	Alzheimer and other	470 [225.2-963.2]	-32.8	10.8
	10	Diabetes mellitus	456.1 [361.2-580]	-21.2	2.7
Umbria - Centre	1	Low back pain	1090.7 [781.9-1468.8]	-31.1	0.8
	2	Headache disorders	827.8 [137.2-1774.2]	-25.4	-2.0
	3	Ischemic heart disease	754.7 [661.6-816.2]	-24.8	4.4
	4	Falls	708.7 [517.3-945.9]	-23.2	4.3
	5	Anxiety disorders	659.1 [455.4-904.3]	-21.4	8.2
	6	Depressive disorders	608.8 [411.8-837.3]	-23.2	15.8
	7	Stroke	511.7 [447.2-561.3]	-23.7	36.1
	8	Alzheimer and other	508.8 [239.1-1042.8]	-17.8	-4.3
	9	Lung cancer	482.4 [434.6-531.6]	-18.1	0.6
	10	Diabetes mellitus	402.1 [322.7-506.4]	-26.7	11.4

Region	Rank (2019)	Cause	DALYs per 100000 [95% UI]	Change 2000 (%)	Change 2021 (%)
Marche - Centre	1	Low back pain	980.8 [680.3-1363.9]	-26.9	3.9
	2	Headache disorders	829.6 [137-1778.7]	-16.9	-4.9
	3	Ischemic heart disease	725.2 [631.7-791.5]	-26.3	4.2
	4	Depressive disorders	609.6 [418-839.7]	-35.3	22.3
	5	Falls	589.4 [434.3-772.2]	-30.0	19.4
	6	Alzheimer and other	507.9 [240.7-1045.2]	-28.2	15.0
	7	Stroke	471 [408.6-520]	-30.0	21.6
	8	Anxiety disorders	456.5 [305.7-636.5]	-31.9	9.2
	9	Lung cancer	448.3 [400.5-493.8]	-10.5	3.0
	10	Other musculoskeletal	410.8 [279.5-578.9]	-12.7	4.2
Lazio - Centre	1	Low back pain	1082.2 [773.7-1451.7]	-34.0	0.9
	2	Ischemic heart disease	839.8 [754.3-903.3]	-23.8	-0.4
	3	Headache disorders	824.2 [136.9-1767.6]	-13.6	0.2
	4	Falls	777.5 [568.9-1027.3]	-15.8	5.8
	5	Anxiety disorders	657.5 [452.2-904.7]	-28.6	19.1
	6	Lung cancer	628.7 [573.5-677.4]	-27.9	22.7
	7	Depressive disorders	611.8 [418.8-839.7]	-27.5	24.5
	8	Diabetes mellitus	511.8 [411.9-640.2]	-24.8	19.0
	9	Stroke	506.3 [450.1-547.6]	-22.7	4.2
	10	Alzheimer and other	501.6 [241.8-1026.4]	-15.3	-1.5
Abruzzo - South	1	Low back pain	1076.4 [771-1435.5]	-19.1	0.8
	2	Ischemic heart disease	888.6 [793.7-958]	-19.0	-3.2
	3	Headache disorders	780.8 [125.3-1709.7]	-18.4	0.1
	4	Falls	745.9 [552.7-982.4]	-19.0	4.7
	5	Anxiety disorders	656.7 [454.3-901]	-27.2	13.5
	6	Depressive disorders	605.9 [411.7-834.5]	-25.2	21.8
	7	Stroke	514.3 [452.2-562.6]	-23.3	38.8
	8	Diabetes mellitus	511 [408.9-639.7]	-21.9	2.9
	9	Alzheimer and other	501.9 [241.4-1016.2]	-17.4	-0.8
	10	Lung cancer	451.6 [410.2-492.2]	-23.5	8.6

Region	Rank (2019)	Cause	DALYs per 100000 [95% UI]	Change 2000 (%)	Change 2021 (%)
Molise - South	1	Low back pain	1070.7 [760.9-1434.4]	-35.3	0.6
	2	Ischemic heart disease	1009.9 [905.9-1098.7]	-10.3	-4.2
	3	Headache disorders	820 [136.4-1746.1]	-25.0	14.6
	4	Falls	806.9 [594.8-1066.9]	-12.6	1.9
	5	Anxiety disorders	654.4 [454.1-903.8]	-29.1	20.9
	6	Stroke	624.5 [555-682.2]	-25.4	24.8
	7	Diabetes mellitus	606.9 [491.2-758.7]	-9.1	23.3
	8	Depressive disorders	602.5 [412.7-827.4]	0.4	1.9
	9	Alzheimer and other	503 [238.8-1029.4]	-14.6	16.4
	10	Lung cancer	485.5 [435.5-533.4]	-13.1	-0.4
Campania - South	1	Ischemic heart disease	1087.6 [983.2-1162.9]	-43.8	-0.8
	2	Low back pain	1065.4 [759.8-1426.4]	-26.0	0.7
	3	Headache disorders	825.2 [135.6-1758.7]	-23.8	28.3
	4	Stroke	715.5 [639.8-772.3]	-20.7	16.0
	5	Diabetes mellitus	693.2 [575.1-850.3]	-17.5	13.2
	6	Falls	688.5 [505.4-918.7]	-13.2	8.4
	7	Lung cancer	684.6 [629.9-737.3]	-5.8	3.6
	8	Anxiety disorders	659 [461.2-907.3]	-5.8	7.2
	9	Depressive disorders	606.2 [413.1-830.7]	-11.5	12.8
	10	Alzheimer and other	508.2 [237.8-1061.1]	-24.4	33.6
Puglia - South	1	Low back pain	1058.4 [753.2-1424]	-22.0	7.5
	2	Headache disorders	825.7 [135.7-1761]	-23.7	28.8
	3	Ischemic heart disease	748.7 [661.5-804.5]	-20.2	11.1
	4	Falls	700.1 [511.7-939.3]	-17.0	12.2
	5	Anxiety disorders	657.1 [457.5-894.5]	-20.8	15.0
	6	Depressive disorders	607.6 [412.4-832.5]	-22.8	22.8
	7	Diabetes mellitus	549.6 [445.1-681.7]	-23.5	27.0
	8	Alzheimer and other	510.8 [239.4-1060.5]	-27.7	13.0
	9	Lung cancer	481.5 [441.7-519]	-28.5	4.6
	10	Stroke	465.9 [410.6-507.8]	-27.9	2.4

Region	Rank (2019)	Cause	DALYs per 100000 [95% UI]	Change 2000 (%)	Change 2021 (%)
Basilicata - South	1	Low back pain	1075.6 [767.1-1451.9]	-26.6	0.7
	2	Ischemic heart disease	856.1 [773.4-925.3]	-25.9	-1.7
	3	Headache disorders	821.1 [136.9-1759.8]	-24.8	-3.4
	4	Anxiety disorders	653.6 [456.1-897]	-25.5	19.5
	5	Falls	632.8 [476.7-825.4]	-24.5	12.4
	6	Diabetes mellitus	627.8 [510.4-777.1]	-14.1	2.7
	7	Depressive disorders	604.8 [415.2-839.1]	-14.7	2.7
	8	Stroke	583.6 [519.2-640]	-12.9	-0.7
	9	Neonatal disorders	515.9 [443.7-592.2]	-19.6	9.5
	10	Alzheimer and other	506.1 [241.3-1054.2]	-14.0	-2.7
Calabria - South	1	Low back pain	1054.1 [751.7-1405.7]	-30.4	0.6
	2	Ischemic heart disease	919.6 [833.9-982.4]	-20.6	-1.9
	3	Headache disorders	826 [136.6-1769.8]	-23.2	1.8
	4	Falls	674.6 [500.1-882.5]	-19.8	20.7
	5	Diabetes mellitus	666.4 [546-823.5]	-16.1	17.8
	6	Anxiety disorders	659.1 [458.9-908.5]	-15.0	11.6
	7	Stroke	623 [555.1-674.2]	-9.8	10.0
	8	Depressive disorders	607.4 [416.7-838.1]	-10.1	10.1
	9	Neonatal disorders	585.2 [530.4-641.1]	-12.8	4.3
	10	Alzheimer and other	497.5 [235.1-1017.9]	-16.0	18.7
Sicilia - Islands	1	Low back pain	1054.8 [742.2-1418.2]	-33.1	0.6
	2	Ischemic heart disease	898.4 [808.9-960.7]	-35.8	14.9
	3	Diabetes mellitus	867.4 [682.1-1080.8]	-19.5	3.0
	4	Headache disorders	768.7 [137.7-1622.4]	-14.4	15.9
	5	Stroke	718.7 [631.2-781.1]	-14.4	16.7
	6	Anxiety disorders	655.8 [457.7-903.6]	-16.3	16.0
	7	Depressive disorders	605.7 [410.3-834.2]	-15.3	20.8
	8	Alzheimer and other	585.5 [277.9-1191]	-15.7	21.5
	9	Lung cancer	555.9 [509.3-597.3]	-18.8	2.5
	10	Falls	527.2 [400.7-675.2]	-21.7	4.4

Region	Rank (2019)	Cause	DALYs per 100000 [95% UI]	Change 2000 (%)	Change 2021 (%)
Sardegna - Islands	1	Low back pain	1071.6 [756.2-1429.4]	-22.6	0.6
	2	Headache disorders	821.7 [136.6-1759]	-24.7	-2.1
	3	Depressive disorders	684.1 [468.3-942.6]	-32.5	14.3
	4	Ischemic heart disease	679.7 [608.8-733.4]	-23.7	-0.3
	5	Diabetes mellitus	613.4 [474.8-785.7]	-27.0	6.5
	6	Falls	612.8 [460.8-795.2]	-19.8	-1.0
	7	Lung cancer	552 [504.5-599.9]	-25.7	5.7
	8	Stroke	515 [455-562.6]	-27.4	11.8
	9	Alzheimer and other	506.9 [245-1031]	-21.6	7.7
	10	Anxiety disorders	488.4 [330-673]	-6.5	5.0

Change 2000: DALY rate change (%) from 2000 to 2019; Change 2021: DALY rate change (%) from 2019 to 2021.
Abbreviations: UI: Uncertainty Interval.

Figure S24. Percentage of family health expenditure over total health expenditure in Italy by region and macro-region from 2000 to 2019.

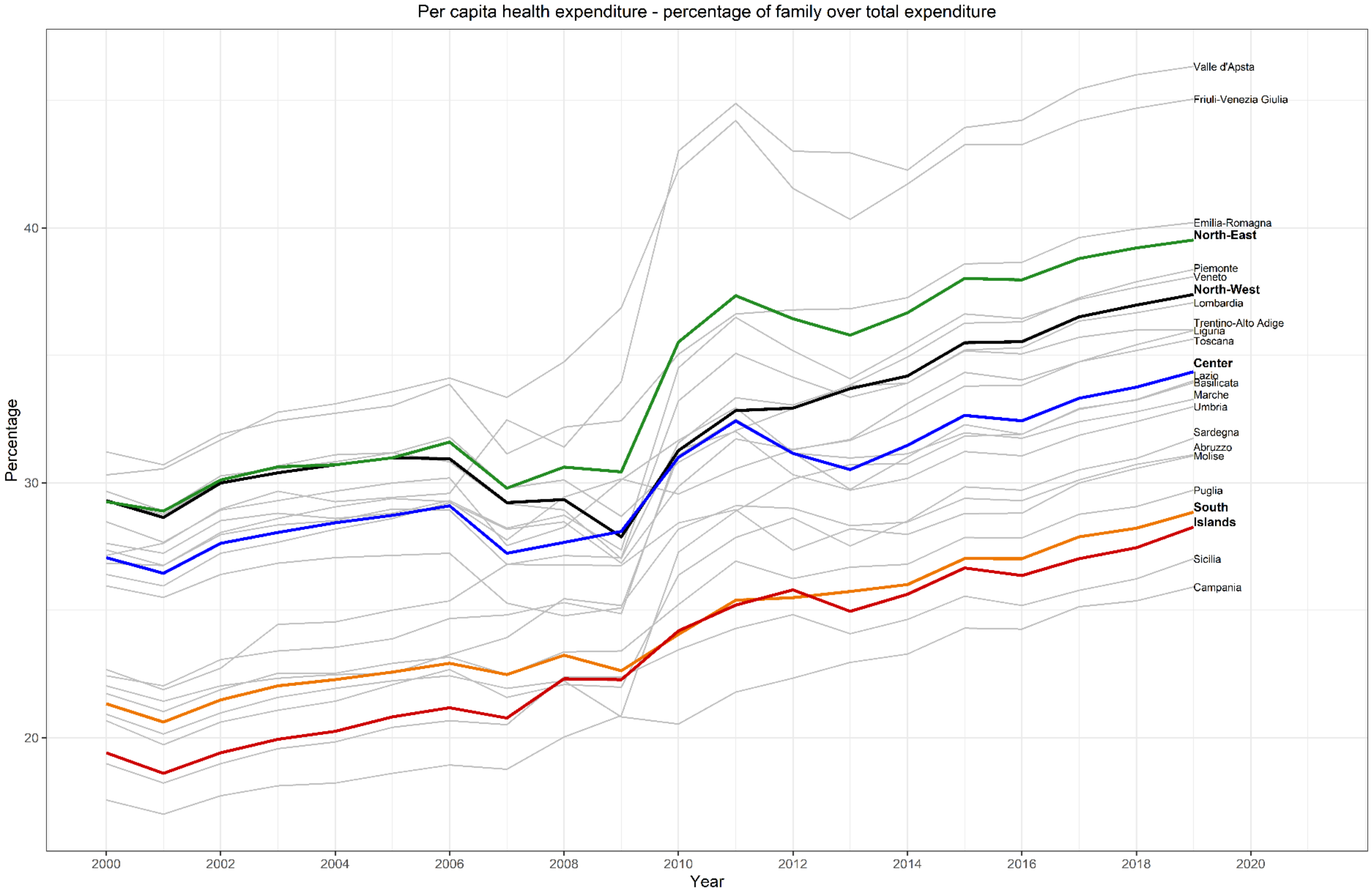

Supplement: Supplementary appendix 2 [file mmc2.pdf]
